# Nickel-Catalyzed Removal of Alkene Protecting Group of Phenols, Alcohols via Chain Walking Process

**DOI:** 10.3390/molecules25030602

**Published:** 2020-01-30

**Authors:** Chenkai Meng, Haolin Niu, Juehan Ning, Wengang Wu, Jun Yi

**Affiliations:** Jiangsu Laboratory of Advanced Functional Material, School of Materials Engineering, Changshu Institute of Technology, Changshu 215500, China; mengchenkai@163.com (C.M.); niuhaolin1@126.com (H.N.); juehanning@126.com (J.N.); 505609053@139.com (W.W.)

**Keywords:** deprotection, nickel, chain walking, phenol, alcohol

## Abstract

An efficient nickel-catalyzed removal of alkene protection group under mild condition with high functional group tolerance through chain walking process has been established. Not only phenolic ethers, but also alcoholic ethers can be tolerated with the retention of stereocenter adjacent to hydroxyl group. The new reaction brings the homoallyl group into a start of new type of protecting group.

## 1. Introduction

**Scheme 1:**
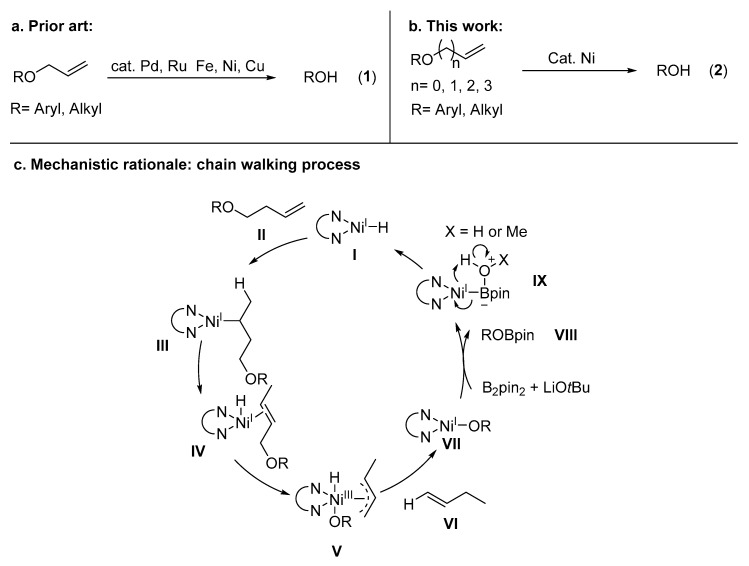
Different removal of alkene protecting group and new mechanistic rationale.

Functional group protecting and deprotecting is a common strategy widely used in organic chemistry especially in total synthesis [1]. The most conventional masking of phenols and alcohols are allylethers derived from facile base-promoted substitution of allyl halides [2]. Meanwhile, allylethers are easily removed under acidic, basic, reductive, or oxidative conditions with or without the aid of transition metals (Scheme 1a) [3,4,5,6,7,8,9,10,11]. On the other hand, it means allylethers are not stable in these conditions which diminished its usage in application. Long-chain alkene ethers such as homoallylethers are significantly steadier than allylethers which have been serving as a protecting group for decades [12,13,14,15,16,17,18], but lack of utility in practice due to the few deprotection methods. Ozonolysis of homoallylic carboxylic esters was reported by Barrett [19]. Cossy [20] and Lipshutz [21] reported removal of homoallyl group by Grubbs-Hoveyda catalyst through isomerization or metathesis. These reports are encountered from harsh reaction conditions. Meanwhile, chain walking strategy in bond construction has been emerging as a powerful tool utilizing alkene as feedstock in synthetic chemistry [22,23,24,25]. Hu [26,27], Martin [28,29,30], Zhu [31,32,33,34,35,36], and Hartwig [37] recently demonstrated that nickel was a prominent catalyst in chain walking chemistry. Streuff reported a zirconium catalyzed deallylation through chain walking mechanism [38]. Inspired by these reports and our interest in group transfer reactions [39] and nickel-catalyzed reactions [40], we envisaged that the nickel-hydride complex **I** formed through en route nickel-boryl [41,42,43] water or methanol addiction intermediate σ-bond metathesis would trigger the chain walking process (**III**, Scheme 1c). Low valent Nickel(I) was reported to go through oxidative addition to allyl C-O bond [44,45,46], which made the formation of intermediate **V** possible. The alkene (**VI**) was released after reductive elimination and regenerated the catalyst. Free phenols or alcohols would be retrieved by simple acid workup of **VIII**. Herein, we report the nickel-catalyzed removal of alkene protection group of phenols and alcohols under mild condition with high functional group tolerance through chain walking process.

## 2. Results

In order to testify our proposal, we began our study by optimizing the reaction conditions using 4-(but-3-enyloxy)biphenyl (**1a**) as the model substrate (Table 1). Investigation of a range of parameters, we found that the combination of nickel(II) chloride ethylene glycol dimethyl ether complex (NiCl_2_∙DME, 5 mol%), 6,6′-dimethyl-2,2′-bipyridine (L1) as ligand, Bis(pinacolato)diboron (B_2_pin_2_, 1.5 equiv) as the reductant, Lithium *tert*-butoxide as the base, in *N,N*-dimethylacetamide (DMA) (0.1M) with methanol and water as additives gave excellent isolated yield (92%) (entry 1, Table 1). Other nickel sources such as zero valent Bis(1,5-cyclooctadiene)nickel (Ni(COD)_2_, entry 2, Table 1) or less soluble NiCl_2_ (entry 3, Table 1) diminished the yield. Similar to the previous reports [31,32,33,34,35,36], more bulky ligand was critical for the reaction and probably facilitate the chain walking process (entry 4–5, Table 1). Other common phosphine ligands such as triphenylphosphine (entry 6, Table 1) and tricyclohexylphosphine (entry 7, Table 1) were all low efficient. Nickel(I) was proposed as the active spices in the chain walking reactions [31,32,33,34,35,36], so we tested some reductant to generate of nickel(I) spices. B_2_pin_2_ in combination of base was reported as efficient reductant in nickel-catalyzed reductive reactions [47], to our pleased moderated yield was observed (entry 8, Table 1). Other widely used metal reductants in nickel-catalyzed cross reductive reaction such as Zn and Mn gave very few product (entry 9–10, Table 1) [48,49,50,51,52]. Next, we surveyed some additives to accelerate the reaction. We found that a certain amount of methanol greatly prompted the yield (entry 11, Table 1). Water was added to further boost the efficiency of the reaction. Both water and methanol might serve as a hydride donor in σ-bond metathesis. Considering both Lithium *tert*-butoxide and water were added, in situ lithium hydroxide might be formed. Directly using lithium hydroxide as base did not have a similar result (entry 13, Table 1). Switching reaction solvent from DMA to less polar solvent such as tetrahydrofuran (THF) resulted in lower yields (entries 15, Table 1). Finally, control experiments showed that both nickel and B_2_pin_2_ were indispensable for the reaction. In order to demonstrate its possibility in large scale synthesis, a 20 mmol scale reaction was done (entry 18, Table 1). The yield was lower but still outstanding. The attempt to lower the loading of nickel catalyst and ligand greatly diminished the yield with substantial starting material left (entry 19, Table 1).

With the optimized condition in hand, the scope of substrate of the reaction was examined (Table 2). First, we tested the phenol scope. *Ortho* methyl group of phenol (**1d**) gave good yields, although *ortho* position was more hindered. Electron effect of the substitution groups did not play great roll on the yield. Strong electron-donating (benzyloxy, **1m**) as well as strong electron-withdrawing (trifluoromethyl, **1p**) groups were all tolerated. More strikingly, fragile groups in basic conditions such as aldehyde (**1o**), ester (**1i**), even free carboxylic acid (**1l**) were all tolerated in the reaction conditions, although the yield of aldehyde (**1o**) was low Furthermore, different halides (**1e**–**1h**) even iodide were untouched during the reactions which could be used in the further elaborating reactions, for instance, Suzuki reactions. It is worth highlighting that heteroaromatics (**1q**, **1r**), which are prevalent structure motifs in medicinal molecular, did not corrupt the efficiency of the reaction.

Next, different alkene protecting groups of phenol were tested (Table 3). Interestingly, vinyl (**3a**–**3c**) was removed at the comparable yield without nickel catalyst indicated another mechanism might be involved. Except for the homoallyl group, the allyl group also could be easily removed under existing conditions (**3d**–**3i**). Interestingly, not only the acyclic alkene protecting groups, but also the cyclic alkene was smoothly detached in outstanding yield (**3i**). Longer alkene protecting group (pentenyl) afforded products albeit in relatively lower yields (**3j**–**3m**). Control experiments showed that nickel catalyst was indispensable for alkene protecting groups longer than vinyl.

**Scheme 2:**
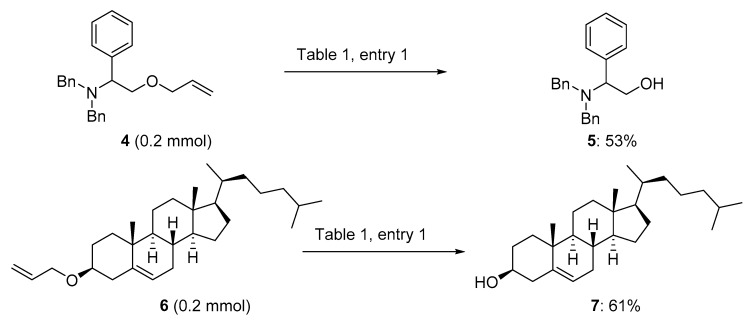
Removal of alcohol protecting groups. Conditions: See Supplementary Materials for details.

We tried to expand the scope from phenols to aliphatic alcohols (Scheme 2). To our delight, allyl protected alcohols were easily removed to give both aminoalcohol (**5**) and cholesterol (**7**) in moderate yield. More importantly, the stereocenter adjacent to hydroxyl of **7** was preserved after the reaction. For the homoallyl ether analogs the yield dropped to around 40% which was not practical in synthetic view.

**Scheme 3:**
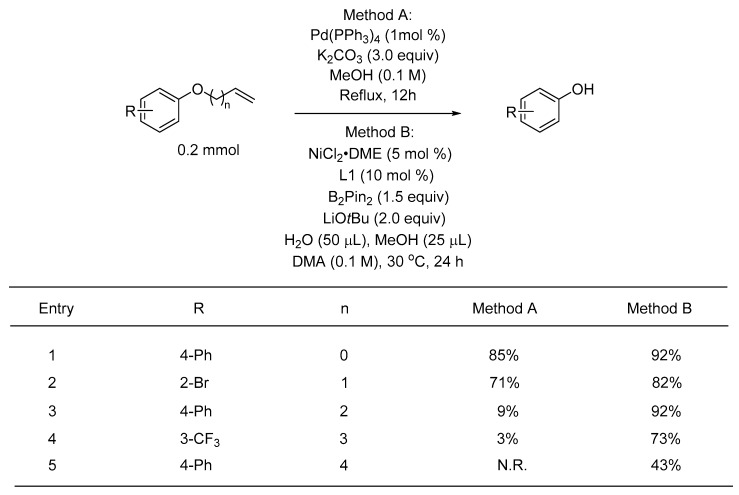
Comparison with previous method.

Taking further advantage of our new reaction (Scheme 3, Method B), we compared it with the classic palladium deprotection protocol (Scheme 3, Method A). These two methods were about the same efficiency when the alkene protecting groups were closed to the oxygen atom (n < 1). In contrast, Method A drastically lost its activity when the interval between the alkene and oxygen is prolonged (n > 2), meanwhile, Method B still maintained medium yield even at n = 4. Through this comparison, it is possible to remove allyl and homoallyl protecting groups sequentially by utilizing these methods.

**Scheme 4:**
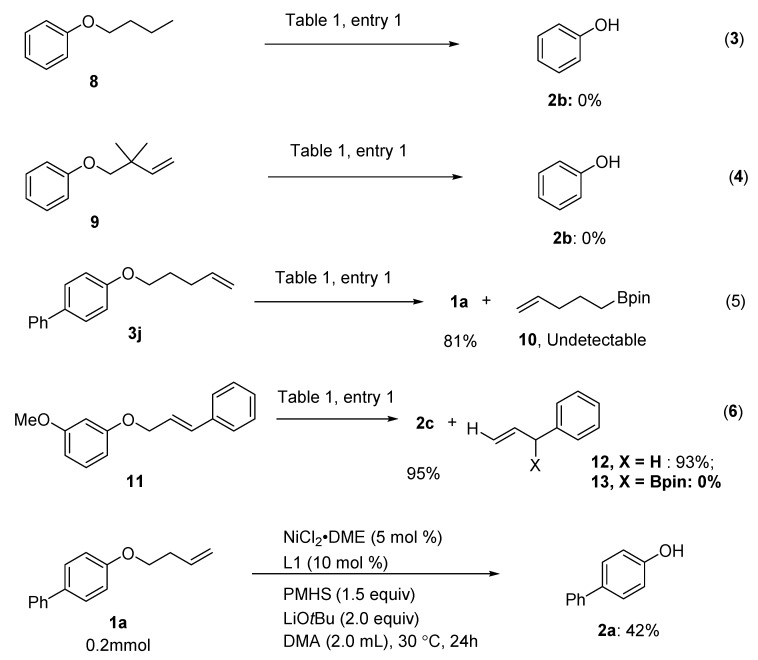
Mechanistic studies. Conditions: See Supplementary Materials for details.

Although the clear panorama of the catalytic cycle was still under pursuing, the control experiments were designed to unveil the reaction mechanism (Scheme 4). First, butoxybenzene (**8**) was put into the optimized condition, no product was detected. It means the coordination of the nickel catalyst of the substrate was necessary. Second, ((2,2-dimethylbut-3-en-1-yl)oxy)benzene (**9**) was subjected to optimal condition, similarly no desire product was observed (**3**), which means the chain walking was intercepted with the quaternary carbon to stop the formation of key allyl ether. When we analyzed the byproduct of the reaction **3j** by GC-MS, the borylated alkene **10** was not detected which means the reaction might not be initiated by Ni-Bpin. Due to the low boiling point of pentene (30 °C), we did not see pentene signal on the GC-MS. Substrate (**11**) was synthesized to get more information of the fate of deprotected group. Allylbenzene (**12**) was detected as equivalent to the product instead of the corresponding borylation product (**13**) indicating the alkene was formed after the deprotection. The combination of B_2_pin_2_ and water/methanol might serve as a hydride donor to form the Ni-H species. A preliminary survey with silane to generate Ni-H in situ [11] was done and a great amount of product was also found which supported the possibility of Ni-H as the true catalyst in the reaction.

## 3. Discussion

To summarize, we reported the nickel-catalyzed removal of alkene protecting groups of phenols and alcohols through chain walking process. The facile and mild reaction condition as well as high functional tolerance highlight its utilization in the future. More importantly, the nonconventional homoallyl may rival as protecting group with this report that is complementary to existing protocols. Future efforts are directed toward expanding this type of new transformation in practice as well as understanding the mechanism of the reaction.

## 4. Experimental Section

### 4.1. Representative General Procedure for Nickel-Catalyzed Deprotecting Reaction

In glovebox, B_2_pin_2_ (76.17 mg, 0.3 mmol, 1.5 equiv), NiCl_2_(DME) (2.20 mg, 0.01 mmol, 0.05 equiv), 6,6-dimethyl-2,2-dipyridyl (3.68 mg, 0.02 mmol, 0.10 equiv), LiO*t*Bu (32 mg, 0.4 mmol, 2.0 equiv), and protected phenol (if solid) (0.2 mmol) were added to an oven-dried tube. The reaction tube was equipped with a magnetic stir bar and sealed with Teflon-lined cap, refilled the system with argon and performed two more evacuation-backfill cycles, protected phenol was added by syringe under argon flow (if liquid). DMA (2.00 mL), H_2_O (50 µL), MeOH (25 µL) were added to the tube sequentially. The reaction mixture turned dark and was stirred at 30 °C for 24 h. When the reaction was finished, 10 mL aqueous HCl (0.1 M) was added to the mixture and extracted with CH_2_Cl_2_ (2 × 10 mL), dried over anhydrous MgSO_4_, and concentrated in vacuo. The resulting residue was purified by silica gel flash chromatography to give the product.

### 4.2. Characterization Data for Products ***2a**–**2s***, ***5*** and ***7*** (Tables 2, 3 and Scheme 2)

*4-Phenylphenol* (**2a**): White solid. IR (cm^−1^, KBr): 3434, 3367, 3130, 1662, 1611, 1522, 1477, 1459, 1401, 1257, 1139, 1069, 998, 954, 862, 833, 758, 686, 555, 517. ^1^H NMR (400 MHz, CDCl_3_) δ 7.57 (d, *J* = 7.4 Hz, 2H), 7.51 (d, *J* = 8.1 Hz, 2H), 7.45 (t, *J* = 7.3 Hz, 2H), 7.35 (d, *J* = 7.1 Hz, 1H), 6.94 (d, *J* = 8.1 Hz, 2H), 4.95 (s, 1H). ^13^C NMR (101 MHz, CDCl_3_) δ 155.0, 140.7, 134.0, 128.7, 128.4, 126.7, 115.6. m.p.: 163–164 °C.

*Phenol* (**2b**): White solid. IR (cm^−1^, KBr): 3440, 3134, 1659, 1624, 1401, 1075, 989, 951, 858, 538. ^1^H NMR (400 MHz, CDCl_3_) δ 7.31 (t, *J* = 7.7 Hz, 2H), 7.02 (t, *J* = 7.3 Hz, 1H), 6.93 (d, *J* = 8.2 Hz, 2H), 6.04 (s, 1H). ^13^C NMR (101 MHz, CDCl_3_) δ 155.2, 129.9, 121.2, 115.6. m.p.: 103–104 °C.

*3-Methoxyphenol* (**2c**): Brown liquid. IR (cm^−1^, KBr): 3439, 3133, 1671, 1609, 1401, 1163, 1073, 991, 946, 856, 769, 689, 541. ^1^H NMR (400 MHz, CDCl_3_) δ 7.16 (t, *J* = 8.0 Hz, 1H), 6.54 (d, *J* = 7.8 Hz, 1H), 6.47 (d, *J* = 7.9 Hz, 2H), 5.65 (s, 1H), 3.80 (s, 3H). ^13^C NMR (101 MHz, CDCl_3_) δ 160.8, 156.7, 130.3, 108.1, 106.6, 101.7, 55.3.

*o-Cresol* (**2d**): Brown liquid. IR (cm^−1^, KBr): 3439, 3134, 1661, 1401, 1075, 993, 952, 858, 536. ^1^H NMR (400 MHz, CDCl_3_) δ 7.28 (d, *J* = 7.2 Hz, 1H), 7.22 (t, *J* = 7.6 Hz, 1H), 7.02 (t, *J* = 7.4 Hz, 1H), 6.90 (d, *J* = 8.0 Hz, 1H), 5.70 (s, 1H), 2.40 (s, 3H). ^13^C NMR (101 MHz, CDCl_3_) δ 153.7, 131.3, 127.3, 124.4, 121.1, 115.3, 15.9.

*3-Fluorophenol* (**2e**): Light yellow liquid. IR (cm^−1^, KBr): 3441, 3134, 1666, 1627, 1131, 1074, 993, 952, 857, 774, 531. ^1^H NMR (400 MHz, CDCl_3_) δ 7.22 (dd, *J* = 15.2, 7.9 Hz, 1H), 6.74–6.57 (m, 3H), 5.76 (s, 1H). ^13^C NMR (101 MHz, Chloroform-*d*) δ 163.7 (d, *J* = 245.6 Hz), 156.3 (d, *J* = 11.3 Hz), 130.7 (d, *J* = 10.0 Hz), 111.3 (d, *J* = 2.9 Hz), 108.2 (d, *J* = 21.3 Hz), 103.3 (d, *J* = 24.7 Hz).

*3-Chlorophenol* (**2f**): Yellow liquid. IR (cm^−1^, KBr): 3439, 3134, 1664, 1623, 1401, 1250, 1138, 1074, 996, 952, 857, 771, 682, 530. ^1^H NMR (400 MHz, CDCl_3_) δ 7.19 (t, *J* = 8.1 Hz, 1H), 6.96 (d, *J* = 8.0 Hz, 1H), 6.89 (s, 1H), 6.75 (dd, *J* = 7.2, 0.9 Hz, 1H), 5.35 (s, 1H). ^13^C NMR (101 MHz, CDCl_3_) δ 155.9, 135.0, 130.6, 121.3, 116.0, 113.8.

*2-Iodophenol* (**2g**): Brown solid. IR (cm^−1^, KBr): 3440, 3133, 1658, 1624, 1401, 1050, 1075, 991, 951, 858, 535. ^1^H NMR (400 MHz, CDCl_3_) δ 7.69 (d, *J* = 7.9 Hz, 1H), 7.27 (t, *J* = 6.4 Hz, 1H), 7.03 (d, *J* = 8.1 Hz, 1H), 6.75–6.63 (m, 1H), 5.36 (s, 1H). ^13^C NMR (101 MHz, CDCl_3_) δ 154.8, 138.3, 130.2, 122.5, 115.2, 85.7. m.p.: 41–42 °C.

*2-Bromophenol* (**2h**): Brown liquid. IR (cm^−1^, KBr): 3439, 3132, 1663. 1619, 1401, 1160, 1077, 993, 946, 858, 744, 539. ^1^H NMR (400 MHz, CDCl_3_) δ 7.49 (d, *J* = 8.0 Hz, 1H), 7.25 (t, *J* = 7.7 Hz, 1H), 7.06 (d, *J* = 8.1 Hz, 1H), 6.84 (t, *J* = 7.7 Hz, 1H), 5.59 (s, 1H). ^13^C NMR (101 MHz, CDCl_3_) δ 152.3, 132.1, 129.2, 121.8, 116.2, 110.3.

*Methyl 4-hydroxybenzoate* (**2i**): White solid. IR (cm^−1^, KBr): 3305, 2963, 2846, 2806, 1919, 1684, 1372, 1114, 954, 851, 691. ^1^H NMR (400 MHz, CDCl_3_) δ 7.98 (d, *J* = 8.5 Hz, 2H), 6.91 (d, *J* = 8.6 Hz, 2H), 6.33 (s, 1H), 3.92 (s, 2H). ^13^C NMR (101 MHz, CDCl_3_) δ 167.3, 160.2, 131.9, 122.3, 115.2, 52.0. m.p.: 130–131 °C.

*Ethyl 3-hydroxybenzoate* (**2j**): Colorless liquid. IR (cm^−1^, KBr): 3350, 2385, 2581, 1692, 1597, 1512, 1459, 1415, 1311, 1232, 1163, 1108, 1074, 926, 881, 760, 670, 565, 522. ^1^H NMR (400 MHz, DMSO) δ 9.83 (s, 1H), 7.43–7.35 (m, 2H), 7.31 (t, J = 7.8 Hz, 2H), 7.03 (d, J = 7.9 Hz, 1H), 4.28 (q, J = 7.0 Hz, 2H), 1.30 (t, J = 7.0 Hz, 3H). ^13^C NMR (101 MHz, CDCl_3_) δ 167.6, 156.3, 131.3, 129.7, 121.6, 120.5, 116.4, 61.6, 14.1.

*4-tert-Butylphenol* (**2k**): White solid. IR (cm^−1^, KBr): 3144, 2962, 2868, 1608, 1513, 1447, 1399, 1269, 1175, 822, 543. ^1^H NMR (400 MHz, CDCl_3_) δ 7.32 (d, *J* = 8.5 Hz, 2H), 6.85 (d, *J* = 8.5 Hz, 2H), 5.53 (s, 1H), 1.36 (s, 9H). ^13^C NMR (101 MHz, CDCl_3_) δ 152.9, 143.7, 126.5, 115.0, 34.1, 31.6. m.p.: 97–99 °C.

*4-Hydroxybenzoic acid* (**2l**): White solid. IR (cm^−1^, KBr): 3382, 3130, 3013, 1676, 1600, 1402, 1321, 1286, 1238, 1160, 1096, 1000, 929, 848, 611, 541. ^1^H NMR (400 MHz, DMSO) δ 12.41 (s, 1H), 10.21 (s, 1H), 7.79 (d, *J* = 8.5 Hz, 2H), 6.82 (d, *J* = 8.5 Hz, 2H). ^13^C NMR (101 MHz, DMSO) δ 167.6, 162.0, 132.0, 121.8, 115.5. m.p.: 213–215 °C.

*4-Benzyloxyphenol* (**2m**): White solid. IR (cm^−1^, KBr): 3429, 3132, 1659, 1614, 1510, 1401, 1236, 1164, 1086, 1012, 818, 734, 520. ^1^H NMR (400 MHz, CDCl_3_) δ 7.48–7.39 (m, 4H), 7.38–7.33 (m, 1H), 6.89 (d, *J* = 8.8 Hz, 2H), 6.78 (d, *J* = 8.8 Hz, 2H), 5.04 (s, 1H). ^13^C NMR (101 MHz, CDCl_3_) δ 153.0, 149.7, 137.2, 128.5, 127.9, 127.5, 116.1, 116.1, 70.8. m.p.: 122–124 °C.

*2-Naphthol* (**2n**): White solid. IR (cm^−1^, KBr): 3501, 3190, 1628, 1590, 1510, 1459, 1401, 1277, 1215, 1168, 955, 845, 813, 741, 476. ^1^H NMR (400 MHz, CDCl_3_) δ 7.84–7.77 (m, 2H), 7.71 (d, *J* = 8.2 Hz, 1H), 7.48 (t, *J* = 7.5 Hz, 1H), 7.39 (t, *J* = 7.4 Hz, 1H), 7.21–7.11 (m, 2H), 5.52 (s, 1H). ^13^C NMR (101 MHz, CDCl_3_) δ 153.3, 134.6, 129.9, 129.0, 127.8, 126.6, 126.4, 123.7, 117.8, 109.6. m.p.: 120–122 °C.

*p-Hydroxybenzaldehyde* (**2o**): White solid. IR (cm^−1^, KBr): 3144, 2962, 1608, 1513, 1399, 1239, 1176, 821, 543. ^1^H NMR (400 MHz, DMSO) δ 10.59 (s, 1H), 9.79 (s, 1H), 7.76 (d, *J* = 8.4 Hz, 2H), 6.93 (d, *J* = 8.4 Hz, 2H). ^13^C NMR (101 MHz, DMSO) δ 191.3, 163.7, 132.5, 128.9, 116.3. m.p.: 116–118 °C.

*3-Trifluoromethylphenol* (**2p**): Yellow liquid. IR (cm^−1^, KBr): 3440, 3132, 1662, 1622, 1401, 1160, 1076, 946, 858, 538. ^1^H NMR (400 MHz, CDCl_3_) δ 7.38 (t, *J* = 7.9 Hz, 1H), 7.23 (d, *J* = 7.7 Hz, 1H), 7.12 (s, 1H), 7.04 (d, *J* = 8.1 Hz, 1H), 5.36 (s, 1H). ^13^C NMR (101 MHz, CDCl_3_) δ 155.3, 132.4 (q, *J* = 32.7 Hz), 130.3, 123.8 (q, *J* = 271.3 Hz), 117.9 (q, *J* = 4.1 Hz), 112.3 (q, *J* = 4.1 Hz).

*tert-Butyl 4-hydroxy-1H-indole-1-carboxylate* (**2q**): Corlorless liquid. IR (cm^−1^, KBr): 3449, 3135, 1664, 1626, 1400, 1136, 1074, 962, 855. ^1^H NMR (400 MHz, CDCl_3_) δ 7.76 (d, *J* = 8.1 Hz, 1H), 7.55 (d, *J* = 3.5 Hz, 1H), 7.18 (t, *J* = 8.1 Hz, 1H), 6.68 (dd, *J* = 10.2, 5.8 Hz, 2H), 5.40 (s, 1H), 1.70 (s, 9H). ^13^C NMR (101 MHz, CDCl_3_) δ 149.8, 148.7, 136.9, 125.1, 124.7, 119.6, 108.3, 107.7, 103.5, 83.8, 28.2.

*6-Hydroxyflavone* (**2r**): White solid. IR (cm^−1^, KBr): 3437, 3128, 3013, 1621, 1569, 1402, 1255, 1077, 835. ^1^H NMR (400 MHz, DMSO) δ 10.02 (s, 1H), 8.08 (d, *J* = 6.9 Hz, 2H), 7.66 (d, *J* = 9.0 Hz, 1H), 7.59 (d, *J* = 6.5 Hz, 3H), 7.34 (d, *J* = 2.2 Hz, 1H), 7.27 (dd, *J* = 9.1, 2.3 Hz, 1H), 6.95 (s, 1H). ^13^C NMR (101 MHz, DMSO) δ 177.4, 162.6, 155.3, 149.8, 132.0, 131.8, 129.5, 126.7, 124.7, 123.5, 120.3, 107.9, 106.4. m.p.: 238–240 °C.

*4-Cyanophenol* (**2s**): White solid. IR (cm^−1^, KBr): 3283, 2229, 1603, 1505, 1441, 1391, 1283, 1221, 1159, 834, 696, 534. ^1^H NMR (400 MHz, DMSO) δ 10.63 (s, 1H), 7.61 (s, 2H), 7.13–6.68 (m, 2H). ^13^C NMR (101 MHz, CDCl_3_) δ 160.6, 134.3, 119.3, 116.5, 102.5. m.p.: 112–114 °C.

*2-(N,N-Dibenzylamino)-2-phenylethan-1-ol* (**2t**): White solid. IR (cm^−1^, KBr): 3440, 3134, 1661, 1624, 1401, 1070. ^1^H NMR (400 MHz, CDCl_3_) δ 7.53–7.19 (m, 15H), 4.17 (t, *J* = 10.6 Hz, 1H), 4.00–3.91 (m, 3H), 3.64 (dd, *J* = 10.6, 5.1 Hz, 1H), 3.18 (d, *J* = 13.4 Hz, 2H), 3.04 (s, 1H). ^13^C NMR (101 MHz, CDCl_3_) δ 139.1, 135.1, 129.3, 129.0, 128.5, 128.4, 128.0, 127.2, 63.0, 60.4, 53.5. m.p.: decomposed.

*Cholesterol* (**7**): White solid. IR (cm^−1^, KBr): 3435, 3132, 2943, 1666, 1624, 1459, 1399, 1057. ^1^H NMR (400 MHz, CDCl_3_) δ 5.37 (d, *J* = 4.3 Hz, 1H), 3.69–3.35 (m, 1H), 2.35–2.19 (m, 2H), 2.01 (t, *J* = 14.8 Hz, 2H), 1.86 (d, *J* = 9.9 Hz, 3H), 1.60–0.85 (m, 34H), 0.70 (s, 3H). ^13^C NMR (101 MHz, CDCl_3_) δ 140.7, 121.7, 71.7, 56.7, 56.1, 50.1, 42.3, 42.3, 39.8, 39.5, 37.2, 36.5, 36.2, 35.8, 31.9, 31.6, 28.2, 28.0, 24.3, 23.8, 22.8, 22.5, 21.1, 19.4, 18.7, 11.8. m.p.: 146–148 °C.

## Figures and Tables

**Table 1:**
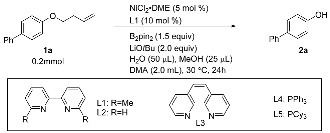
Optimization of removal homoallyl group.

entry	Deviation from standard conditions	Yield of **2** (%) ^a^
1	None	96(92) ^b^
2	Ni(COD)_2_	84
3	NiCl_2_ instead of NiCl_2_(DME)	53
4	L2 instead of L1	37
5	L3	9
6	L4	42
7	L5	45
8	B_2_pin_2_ w/o additives	61
9	Zn instead of B_2_pin_2_	5<
10	Mn instead of B_2_pin_2_	5<
11	No CH_3_OH	73
12	No H_2_O	78
13	LiOH instead of LiO*t*Bu	71
14	KOH instead of LiO*t*Bu	68
15	THF instead of DMA	56
16	No Ni	0
17	No B_2_pin_2_	0
18	20 mmol scale	86
19	1% loading of nickel, 2% L1	52(42) ^c^

^a^ Conditions: 1a (0.2 mmol), Amine (1.5 equiv), Determined by dibromomethane as an internal standard by ^1^H NMR. ^b^ Isolated yield. ^c^ 1a remained in reaction.

**Table 2:**
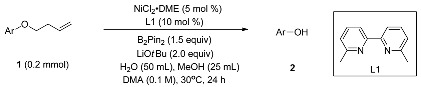
Scope of phenol. Conditions: See Supplementary Materials for details.

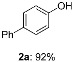	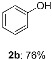	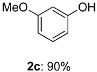	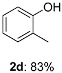
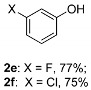	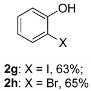	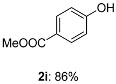	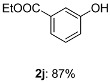
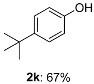	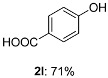	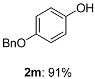	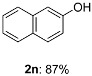
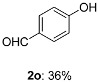	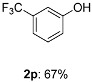	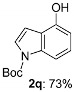	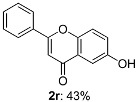

**Table 3:**
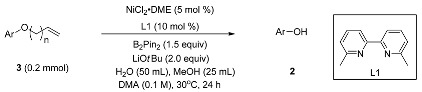
Scope of alkene protecting groups. Conditions: ^a^ Without Ni catalyst and ligand. See Supplementary Materials for details.

Substrate	Product	Substrate	Product
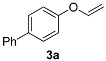	**2a**: 92%, 88% ^a^	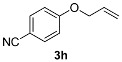	**2s**: 96%
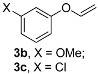	**2c**: 87%, 90% ^a^; **2f**: 92%,84% ^a^	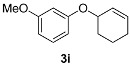	**2c**: 93%
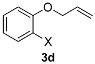	**2g**: X = I, 63%; **2h**: X = Br, 65%	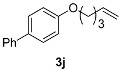	**2a**: 81%
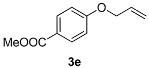	**2i**: 95%	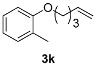	**2d**: 74%
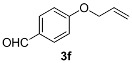	**2o**: 83%	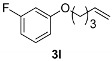	**2e**: 72%
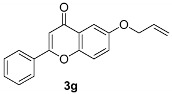	**2r**: 53%	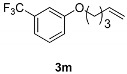	**2p**: 63%

## Supplementary Materials

The followings are available online.
