# Supplementary material for: Nickel-Catalyzed Removal of Alkene Protecting Group of Phenols, Alcohols via Chain Walking Process
Source: Molecules. 2020 Jan 30;25(3):602. doi: 10.3390/molecules25030602 (PMC7037277; doi:10.3390/molecules25030602)
Supplement: Supplementary file 1 [file molecules-25-00602-s001.pdf]

Supporting Information

1. General Information

All the reagents are commercially available, weighted out under ambient conditions and used without further purification except catalyst. All reactions were carried out under argon atmosphere in oven-dried glassware with magnetic stirring. The reactions were monitored through layer chromatography (TLC) on precoated silica gel F254 plates. Visualization on TLC was achieved with UV light (254 nm) and $KMnO_4$ stain as the visualization method. Purification of the product was performed by flash column chromatography using silica gel (300-400 mesh). ^1H NMR spectra were recorded on a Bruker AVANCE Ⅲ HD 400 (400 MH_Z). ^1H NMR chemical shifts (δ) are expressed in ppm referenced internally to residual solvent signal (CH_3Cl_3, δ = 7.26 ppm); Data for ^1H NMR are reported as follows: Signal splitting were septet as following abbreviations:singlet (s), doublet (d), triplet (t), quartet (q), quintet (quintet), sextet (sex), septet (sept), doublet of doublets (dd), doublet of triplets (dt), doublet of doublet of doublets (ddd), multiplet (m), broad singlet (bs), broad doublet (bd). ^{13}C NMR are reported in trems of chemcial shift. Infrared spectra were obtained either neat or by thin-film, on KBr plates and reported as cm^{-1}. Melting point was measured by Shanghai Jiahang JHX-5 Plus. High-resolution mass spectral analysis (HRMS) data were acquired on Water XEVO G2 Q-TOF (Waters Corporation).

2. Synthetic Procedure

2.1. Preparation of Starting Ethers

2.2.1. General Procedure A

To a solution of phenol (10.00 mmol) and K_2CO_3 (30.00 mmol) in acetone (30.00 mL) was added 4-bromo-but-1-ene (1.22 mL, 12.00 mmol). The mixture was refluxed for 12 h. It was then cooled to RT and the solvent was removed in vacuo. The residue was partitioned between CH_2Cl_2 and saturated brine and the aqueous layer was extracted with CH_2Cl_2 (2 x 10 mL). The combined organic extracts were washed with brine (2 x 10 mL), dried and concentrated in vacuo. The resulting residue was purified by silica gel flash chromatography and concentrated in vacuo to provide the title compounds.

2.2.2. General Procedure B

To a solution of phenol (10.00 mmol) and, K_2CO_3 (4.15 g, 30.00 mmol) and 1,2-Dibromoethane (2.59 mL, 30 mmol) in acetone (30.00 mL) and the mixture was refluxed for 12 h. Column chromatography using silica gel (eluting hexane) afforded the crude product. Then, the crude product reacted with potassium *tert*-butoxide (1.12g, 10mmol) in THF (20.00 mL) at RT for 10 min. The resulting residue was purified by silica gel flash chromatography and concentrated in vacuo to provide the title compounds.

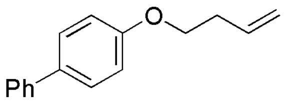

4-(But-3-enyloxy)biphenyl. The titled product was prepared by following General Procedure A using biphenyl-4-ol (1.70 g, 10 mmol), K_2CO_3 (4.15 g, 30.00 mmol) and 4-bromo-but-1-ene (1.22 mL, 12mmol) in acetone (30.00 mL) and the mixture was refluxed for 12 h. Column chromatography using silica gel (eluting with hexane) afforded the product **1a** (1.35 g, 60%) as colorless crystal. The compound has been reported.[1]

^{1}H NMR (400 MHz, $CDCl_3$) δ 7.58 (d, *J* = 7.7 Hz, 2H), 7.55 (d, *J* = 8.6 Hz, 2H), 7.44 (t, J = 7.6 Hz, 2H), 7.33 (t, J = 7.3 Hz, 1H), 7.01 (d, J = 8.5 Hz, 2H), 5.96 (dq, J = 10.1, 6.7 Hz, 1H), 5.19 (dd, J = 26.4, 13.7 Hz, 2H), 4.10 (t, J = 6.7 Hz, 2H), 2.61 (q, J = 6.6 Hz, 2H).

^{13}C NMR (101 MHz, $CDCl_3$) δ 158.5, 140.8, 134.4, 133.8, 128.7, 128.1, 126.7, 126.6, 117.0, 114.9, 67.3, 33.7.

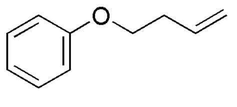

(But-3-enyloxy)benzene. The titled product was prepared by following General Procedure A using phenol (0.94 g, 10 mmol), K_2CO_3 (4.15 g, 30.00 mmol) and 4-bromo-but-1-ene (1.22 mL, 12mmol) in acetone (30.00 mL) and the mixture was refluxed for 12 h. Column chromatography using silica gel (eluting with hexane) afforded the product **1b** (1.41 g, 95%) as a colorless liquid. The compound has been reported.[2]

^{1}H NMR (400 MHz, $CDCl_3$) δ 7.32 (dd, *J* = 8.5, 7.5 Hz, 2H), 7.07–6.86 (m, 3H), 5.95 (dd, *J* = 17.0, 10.3, , 1H), 5.18 (dd, *J* = 13.7, 11.4, , 2H), 4.06 (t, *J* = 6.7 Hz, 2H), 2.59 (q, *J* = 6.7 Hz, 2H).
^{13}C NMR (101 MHz, $CDCl_3$) δ 158.9, 134.5, 129.4, 120.7, 116.9, 114.6, 67.1, 33.6.

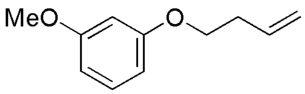

1-(But-3-enyloxy)-3-methoxybenzene. The titled product was prepared by following General Procedure A using 3-Methoxyphenol (1.24 g, 10 mmol), K_2CO_3 (4.15 g, 30.00 mmol) and 4-bromo-but-1-ene (1.22 mL, 12 mmol) in acetone (30.00 mL) and the mixture was refluxed for 12 h. Column chromatography using silica gel (eluting with 3% EtOAc/hexane) afforded the product **1c** (1.23 g, 69%) as a colorless liquid. The compound has been reported.[3]
^{1}H NMR (400 MHz, $CDCl_3$) δ 7.20 (t, *J* = 8.2 Hz, 1H), 6.53 (dd, *J* = 13.5, 5.3 Hz, 3H), 6.02–5.78 (m, 1H), 5.16 (dd, *J* = 24.6, 13.7 Hz, 2H), 4.03 (t, *J* = 6.7 Hz, 2H), 3.81 (s, 3H), 2.57 (q, *J* = 6.6 Hz, 2H).
^{13}C NMR (101 MHz, $CDCl_3$) δ 160.8, 160.1, 134.4, 129.8, 116.9, 106.7, 106.3, 101.0, 67.2, 55.2, 33.6.

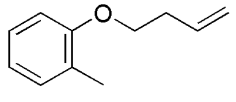

1-(But-3-enyloxy)-2-methylbenzene. The titled product was prepared by following General Procedure A using 2-methylphenol (1.08 g, 10 mmol), K_2CO_3 (4.15 g, 30.00 mmol) and 4-bromo-but-1-ene (1.22 mL, 12 mmol) in acetone (30.00 mL) and the mixture was refluxed for 12 h. Column chromatography using silica gel (eluting with 3% EtOAc/hexane) afforded the product **1d** (1.38 g, 85%) as a colorless liquid. The compound has been reported.[4]
^{1}H NMR (400 MHz, $CDCl_3$) δ 7.20 (t, *J* = 6.9 Hz, 2H), 6.96–6.80 (m, 2H), 6.00 (ddt, *J* = 17.0, 10.3, 6.7 Hz, 1H), 5.28–5.11 (m, 2H), 4.08 (t, *J* = 6.6 Hz, 2H), 2.62 (dd, *J* = 13.2, 6.6 Hz, 2H), 2.29 (s, 3H).
^{13}C NMR (101 MHz, $CDCl_3$) δ 157.0, 134.7, 130.6, 126.9, 126.7, 120.3, 116.8, 111.1, 67.2, 33.9, 16.1.

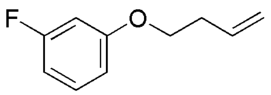

1-(But-3-enyloxy)-3-fluorobenzene. The titled product was prepared by following General Procedure A using 3-fluorophenol (1.12 g, 10 mmol), K_2CO_3 (4.15 g, 30.00 mmol) and 4-bromo-but-1-ene (1.22 mL, 12 mmol) in acetone (30.00 mL) and the mixture was refluxed for 12 h. Column chromatography using silica gel (eluting with hexane) afforded the product **1e** (1.51 g, 91%) as a colorless liquid. The compound has been reported.[5]
^{1}H NMR (400 MHz, $CDCl_3$) δ 7.24 (dd, *J* = 15.4, 7.9 Hz, 1H), 6.77–6.51 (m, 3H), 6.00–5.82 (m, 1H), 5.17 (dd, *J* = 22.3, 13.7 Hz, 2H), 4.02 (t, *J* = 6.7 Hz, 2H), 2.57 (q, *J* = 6.6 Hz, 2H).
^{13}C NMR (101 MHz, $CDCl_3$) δ 163.6 (d, *J* = 244.7 Hz), 160.3 (d, *J* = 10.9 Hz), 134.1, 130.1 (d, *J* = 10.2 Hz), 117.1, 110.3 (d, *J* = 2.9 Hz), 107.4 (d, *J* = 21.3 Hz), 102.2 (d, *J* = 24.5 Hz), 67.4, 33.4.

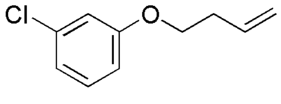

1-(But-3-enyloxy)-3-chlorobenzene. The titled product was prepared by following General Procedure A using 3-chlorophenol (1.29 g, 10 mmol), K_2CO_3 (4.15 g, 30.00 mmol) and 4-bromo-but-1-ene (1.22 mL, 12 mmol) in acetone (30.00 mL) and the mixture was refluxed for 12 h. Column chromatography using silica gel (eluting with hexane) afforded the product **1f** (1.37 g, 75%) as a colorless liquid. The compound has been reported.[6]

^{1}H NMR (400 MHz, $CDCl_3$) δ 7.22 (t, *J* = 8.1 Hz, 1H), 7.00–6.91 (m, 2H), 6.85–6.76 (m, 1H), 5.93 (ddt, *J* = 17.0, 10.3, 6.7 Hz, 1H), 5.32–5.00 (m, 2H), 4.03 (t, *J* = 6.7 Hz, 2H), 2.57 (tt, *J* = 3.9, 2.5 Hz, 2H).

^{13}C NMR (101 MHz, $CDCl_3$) δ 159.7, 134.8, 134.1, 130.1, 120.8, 117.2, 115.0, 113.1, 67.4, 33.5.

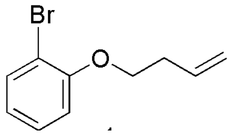

1-(But-3-enyloxy)-2-bromobenzene. The titled product was prepared by following General Procedure A using 2-bromophenol (1.73 g, 10 mmol), K_2CO_3 (4.15 g, 30.00 mmol) and 4-bromo-but-1-ene (1.22 mL, 12 mmol) in acetone (30.00 mL) and the mixture was refluxed for 12 h. Column chromatography using silica gel (eluting with hexane) afforded the product 1**g** (1.93 g, 87%) as a colorless liquid. The compound has been reported.[7]

^{1}H NMR (400 MHz, $CDCl_3$) δ 7.59–7.50 (m, 1H), 7.26 (d, *J* = 7.4 Hz, 1H), 6.92 (d, *J* = 8.1 Hz, 1H), 6.87–6.81 (m, 1H), 5.99 (ddd, *J* = 14.7, 10.1, 7.2 Hz, 1H), 5.19 (dd, *J* = 28.5, 13.7 Hz, 2H), 4.14–4.04 (m, 2H), 2.63 (dd, *J* = 3.8, 2.6 Hz, 2H).

^{13}C NMR (101 MHz, $CDCl_3$) δ 155.3, 134.2, 133.4, 128.4, 121.9, 117.3, 113.4, 112.4, 68.5, 33.6.

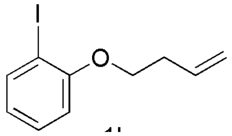

1-(But-3-enyloxy)-2-iodobenzene. The titled product was prepared by following General Procedure A using 2-iodophenol (1.73 g, 10 mmol), K_2CO_3 (4.15 g, 30.00 mmol) and 4-bromo-but-1-ene (1.22 mL, 12 mmol) in acetone (30.00 mL) and the mixture was refluxed for 12 h. Column chromatography using silica gel (eluting with hexane) afforded the product **1h** (2.02 g, 74%) as a colorless liquid. The compound has been reported.[8]

^{1}H NMR (400 MHz, $CDCl_3$) δ 7.80 (d, *J* = 7.7 Hz, 1H), 7.30 (d, *J* = 8.7 Hz, 1H), 6.83 (d, *J* = 8.2 Hz, 1H), 6.73 (t, *J* = 7.5 Hz, 1H), 6.14–5.85 (m, 1H), 5.21 (dd, *J* = 30.3, 13.7 Hz, 2H), 4.09 (t, *J* = 6.6 Hz, 2H), 2.64 (q, *J* = 6.6 Hz, 2H).

^{13}C NMR δ 157.4, 139.5, 134.3, 129.4, 122.5, 117.3, 112.2, 86.8, 68.6, 33.6.

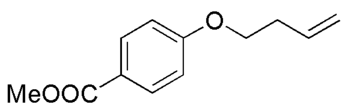

4-(4-methoxycarbonylphenoxy)but-1-ene. The titled product was prepared by following General Procedure A using 4-hydroxy-benzoicacimethylester (1.52 g, 10 mmol), K_2CO_3 (4.15 g, 30.00 mmol) and 4-bromo-but-1-ene (1.22 mL, 12mmol) in acetone (30.00 mL) and the mixture was refluxed for 12 h. Column chromatography using silica gel (eluting with 5% EtOAc/hexane) afforded the product **1i** (1.67 g, 81%) as a colorless liquid. The compound has been reported.[9]

^{1}H NMR (400 MHz, $CDCl_3$) δ 7.99 (d, *J* = 8.7 Hz, 2H), 6.92 (d, *J* = 8.7 Hz, 2H), 5.91 (ddt, *J* = 17.0, 10.1, 6.7 Hz, 1H), 5.25–5.06 (m, 2H), 4.07 (t, *J* = 6.7 Hz, 2H), 3.89 (s, 3H), 2.57 (q, *J* = 6.6 Hz, 2H).

^{13}C NMR (101 MHz, $CDCl_3$) δ 166.8, 162.7, 134.0, 131.5, 122.5, 117.2, 114.1, 67.3, 51.8, 33.4.

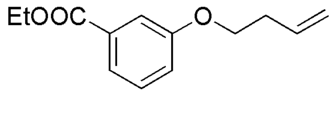

ethyl 3-(but-3-en-1-yloxy)benzoate. The titled product was prepared by following General Procedure A using 4-hydroxy-benzoicacimethylester (1.52 g, 10 mmol), K_2CO_3 (4.15 g, 30.00 mmol) and 4-bromo-but-1-ene (1.22 mL, 12 mmol) in acetone (30.00 mL) and the mixture was refluxed for 12 h. Column chromatography using silica gel (eluting with 5% EtOAc/hexane) afforded the product **1j** (1.76 g, 80%) as a colorless liquid. The compound has been reported.[10]

^{1}H NMR (400 MHz, $CDCl_3$) δ 7.66 (d, *J* = 7.7 Hz, 1H), 7.59 (s, 1H), 7.35 (t, *J* = 7.9 Hz, 1H), 7.11 (dd, *J* = 8.2, 1.6 Hz, 1H), 5.94 (ddt, *J* = 17.0, 10.2, 6.7 Hz, 1H), 5.17 (dd, *J* = 24.0, 13.7 Hz, 2H), 4.40 (q, *J* = 7.1 Hz, 2H), 4.09 (t, *J* = 6.7 Hz, 2H), 2.58 (q, *J* = 6.6 Hz, 2H), 1.42 (t, *J* = 7.1 Hz, 3H).

^{13}C NMR (101 MHz, $CDCl_3$) δ 166.5, 158.8, 134.2, 131.8, 129.3, 121.9, 119.7, 117.1, 114.8, 67.4, 61.0, 33.5, 14.3.

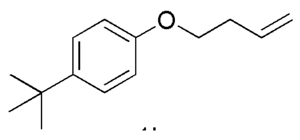

1-(but-3-enyloxy)-4-tert-butylbenzene. The titled product was prepared by following General Procedure A using 4-tert-Butylphenol (1.50 g, 10 mmol), K_2CO_3 (4.15 g, 30.00 mmol) and 4-bromo-but-1-ene (1.22 mL, 12 mmol) in acetone (30.00 mL) and the mixture was refluxed for 12 h. Column chromatography using silica gel (eluting hexane) afforded the product **1k** (1.55 g, 76%) as a colorless liquid. The compound has been reported.[1]

^{1}H NMR (400 MHz, $CDCl_3$) δ 7.33 (d, *J* = 8.7 Hz, 2H), 6.88 (d, *J* = 8.7 Hz, 2H), 5.95 (ddt, *J* = 17.0, 10.3, 6.7 Hz, 1H), 5.17 (dd, *J* = 26.8, 13.7 Hz, 2H), 4.04 (t, *J* = 6.7 Hz, 2H), 2.57 (q, *J* = 6.7 Hz, 2H), 1.34 (s, 9H).

^{13}C NMR (101 MHz, $CDCl_3$) δ 156.6, 143.3, 134.6, 126.2, 116.8, 114.0, 67.2, 34.0, 33.7, 31.5.

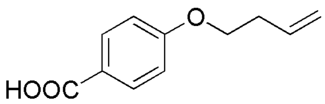

4-(But-3-enyloxy)benzoic acid. The titled product was prepared from 4-(4-methoxycarbonylphenoxy)but-1-ene (1i). 4-(4-methoxycarbonylphenoxy)but-1-ene (2.06 g 10 mmol), potassium *tert*-butoxide (1.68 g 15 mmol), was added to the THF (30 mL), The mixture stired at RT for 24h, give the product **1l** (1.82 g, 95%) as a wihte solid. The compound has been reported.[9]

^{1}H NMR (400 MHz, DMSO) δ 12.60 (s, 1H), 7.88 (d, *J* = 8.5 Hz, 2H), 7.01 (d, *J* = 8.6 Hz, 2H), 5.89 (dq, *J* = 10.0, 6.7 Hz, 1H), 5.14 (dd, *J* = 33.0, 13.7 Hz, 2H), 4.10 (t, *J* = 6.5 Hz, 2H), 2.49 (d, *J* = 7.0 Hz, 2H).

^{13}C NMR (101 MHz, DMSO) δ 167.4, 162.5, 135.1, 131.8, 123.4, 117.6, 114.7, 67.4, 33.3.

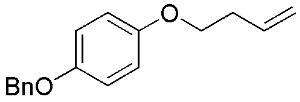

1-(Benzyloxy)-4-(But-3-enyloxy)-benzene. The titled product was prepared by following General Procedure A using 4-(but-3-enyloxy)phenol (2.00g, 10 mmol), K_2CO_3 (4.15 g, 30.00 mmol) and 4-bromo-but-1-ene (1.22 mL, 12 mmol) in acetone (30.00 mL) and the mixture was refluxed for 12 h. Column chromatography using silica gel (eluting with 10% EtOAc/hexane) afforded the product **1m** (1.93 g, 76%) as a colorless liquid. HRMS (ESI) calcd for $C_{17}H_{19}O_2^+[(M+H)^+]$ 255.1380, found 255.1378.

^{1}H NMR (400 MHz, $CDCl_3$) δ 7.45 (d, *J* = 7.3 Hz, 2H), 7.40 (t, *J* = 7.3 Hz, 2H), 7.37–7.32 (m, 1H), 6.93 (d, *J* = 9.0 Hz, 2H), 6.86 (d, *J* = 9.0 Hz, 2H), 6.00–5.86 (m, 1H), 5.16 (dd, *J* = 24.1, 13.7 Hz, 2H), 5.04 (s, 2H), 3.99 (t, *J* = 6.7 Hz, 2H), 2.54 (q, *J* = 6.6 Hz, 2H).

^{13}C NMR (101 MHz, $CDCl_3$) δ 153.3, 153.0, 137.3, 134.6, 128.5, 127.9, 127.5, 116.9, 115.8, 115.6, 70.7, 67.9, 33.8.

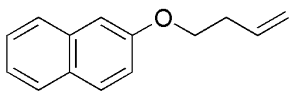

2-(But-3-enyloxy)naphthalene. The titled product was prepared by following General Procedure A using 2-naphthol (1.44 g, 10 mmol), K_2CO_3 (4.15 g, 30.00 mmol) and 4-bromo-but-1-ene (1.22 mL, 12mmol) in acetone (30.00 mL) and the mixture was refluxed for 12 h. Column chromatography using silica gel (eluting hexane) afforded the product **1n** (1.55 g, 61%) as a colorless liquid. The compound has been reported.[1]

^{1}H NMR (400 MHz, $CDCl_3$) δ 7.82-7.68 (m, 3H), 7.49 (t, *J* = 7.4 Hz, 1H), 7.38 (t, *J* = 7.4 Hz, 1H), 7.24–7.13 (m, 2H), 6.01 (ddt, *J* = 17.0, 10.3, 6.7 Hz, 1H), 5.23 (dd, *J* = 27.4, 13.7 Hz, 2H), 4.18 (t, *J* = 6.7 Hz, 2H), 2.67 (q, *J* = 6.7 Hz, 2H).

^{13}C NMR (101 MHz, $CDCl_3$) δ 156.9, 134.6, 134.5, 129.4, 129.0, 127.6, 126.7, 126.3, 123.6, 119.0, 117.1, 106.7, 67.2, 33.6.

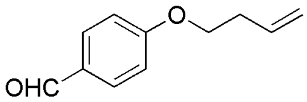

4-(But-3-enyloxy)benzaldehyde. The titled product was prepared by following General Procedure A using 4-hydroxybenzaldehyde (1.22 g, 10 mmol), K_2CO_3 (4.15 g, 30.00 mmol) and 4-bromo-but-1-ene (1.22 mL, 12mmol) in acetone (30.00 mL) and the mixture was refluxed for 12 h. Column chromatography using silica gel (eluting with 10% EtOAc/hexane) afforded the product **1o** (1.29 g, 73%) as a colorless liquid. The compound has been reported.[11]

^{1}H NMR (400 MHz, $CDCl_3$) δ 9.90 (s, 1H), 7.85 (d, J = 8.6 Hz, 2H), 7.02 (d, J = 8.6 Hz, 2H), 5.92 (dq, J = 10.1, 6.7 Hz, 1H), 5.18 (dd, J = 21.5, 13.7 Hz, 2H), 4.12 (t, J = 6.7 Hz, 2H), 2.60 (q, J = 6.6 Hz, 2H).

^{13}C NMR (101 MHz, $CDCl_3$) δ 190.7, 163.9, 133.8, 131.9, 129.9, 117.4, 114.7, 67.5, 33.3.

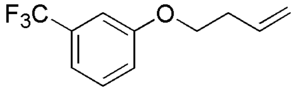

1-but-3-enoxy-3-(trifluoromethyl)benzene. The titled product was prepared by following General Procedure A using 4-Trifluoromethylphenol (1.62 g, 10 mmol), K_2CO_3 (4.15 g, 30.00 mmol) and 4-bromo-but-1-ene (1.22 mL, 12 mmol) in acetone (30.00 mL) and the mixture was refluxed for 12 h. Column chromatography using silica gel (eluting hexane) afforded the product **1p** (1.54 g, 71%) as a colorless liquid. HRMS (ESI) calcd for $C_{11}H_{12}F_3O^+[(M+H)^+]$ 217.0835, found 217.0841.

^{1}H NMR (400 MHz, $CDCl_3$) δ 7.41 (t, J = 8.0 Hz, 1H), 7.24 (d, J = 7.7 Hz, 1H), 7.18 (s, 1H), 7.10 (dd, J = 8.3, 2.2 Hz, 1H), 5.95 (ddt, J = 17.0, 10.3, 6.7 Hz, 1H), 5.26–5.13 (m, 2H), 4.08 (t, J = 6.7 Hz, 2H), 2.60 (q, J = 6.7 Hz, 2H).

^{13}C NMR (101 MHz, $CDCl_3$) δ 159.0, 134.0, 131.8 (q, J = 32.3 Hz), 129.9, 124.0 (q, J = 272.3 Hz), 118.0 (d, J = 1.5 Hz), 117.3 (q, J = 3.9 Hz), 117.2, 111.3 (q, J = 3.9 Hz), 67.5, 33.5.

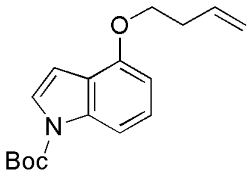

5-But-3-enyloxy-indole-1-carboxylic acid tert-butyl ester. The titled product was prepared by General Procedure A using 4-Hydroxyindole (1.33 g, 10 mmol), K_2CO_3 (4.15 g, 30.00 mmol) and 4-bromo-but-1-ene (1.22 mL, 12 mmol) in acetone (30.00 mL) and the mixture was refluxed for 12 h. Column chromatography using silica gel (eluting 10% EtOAc/Hexane) afforded the crude product, without further purified.

Then, Di-tert-butyl dicarbonate (4.37 g, 20 mmol), Et_3N (3.04g, 30 mmol) in acetonitrile (30.00 mL) and the mixture reacted at RT for 12 h, Column chromatography using silica gel (eluting 5% EtOAc/hexane) afforded the product **1q** (2.41 g, 84%) as a colorless liquid. HRMS (ESI) calcd for $C_{17}H_{22}NO_3^+[(M+H)^+]$ 288.1594, found 288.1603.

¹H NMR (400 MHz, $CDCl_3$) δ 7.77 (d, *J* = 8.2 Hz, 1H), 7.51 (d, *J* = 3.3 Hz, 1H), 7.23 (t, *J* = 8.1 Hz, 1H), 6.70 (dd, *J* = 14.0, 5.8 Hz, 2H), 6.05–5.91 (m, 1H), δ 5.18 (dd, *J* = 31.2, 13.7 Hz, 2H), 4.18 (t, *J* = 6.7 Hz, 2H), 2.64 (q, *J* = 6.6 Hz, 2H), 1.69 (s, 9H).
¹³C NMR (101 MHz, $CDCl_3$) δ 152.2, 149.9, 136.6, 134.5, 125.0, 124.3, 121.1, 117.0, 108.3, 104.4, 104.1, 83.6, 67.4, 33.7, 28.2.

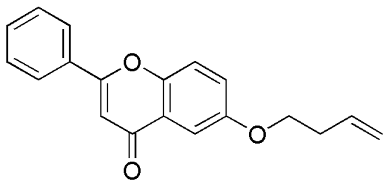

6-But-3-enyloxy-2-phenyl-chromen-4-one. The titled product was prepared by following General Procedure A using 6-Hydroxyflavone (2.38 g, 10 mmol), K_2CO_3 (4.15 g, 30.00 mmol) and 4-bromo-but-1-ene (1.22 mL, 12 mmol) in acetone (30.00 mL) and the mixture was refluxed for 12 h. Column chromatography using silica gel (eluting hexane) afforded the product **1r** (2.81 g, 96%) as a colorless crystals. HRMS (ESI) calcd for $C_{19}H_{17}O_3^+$[$(M+H)^+$] 293.1172, found 293.1177.
¹H NMR (400 MHz, DMSO) δ 8.09 (dd, *J* = 6.1, 1.6 Hz, 2H), 7.74 (d, *J* = 10.0 Hz, 1H), 7.59 (m, 3H), 7.47–7.35 (m, 2H), 7.01 (s, 1H), 6.00–5.83 (m, 1H), 5.15 (dd, *J* = 34.4, 13.7 Hz, 2H), 4.14 (t, *J* = 6.4 Hz, 2H), 2.54 (d, *J* = 6.2 Hz, 2H).
¹³C NMR (101 MHz, DMSO) δ 177.3, 162.7, 156.3, 150.8, 135.1, 132.1, 131.6, 129.5, 126.7, 124.4, 123.9, 120.5, 117.5, 106.5, 106.0, 67.8, 33.3.

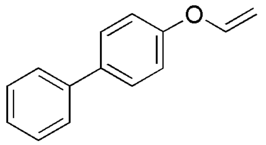

4-Vinyloxy-biphenyl. The titled product was prepared by following General Procedure B using 4-Phenylphenol (1.70 g, 10 mmol), K_2CO_3 (4.15 g, 30.00 mmol) and 1,2-Dibromoethane (2.59 mL, 30 mmol) in acetone (30.00 mL) and the mixture was refluxed for 12 h. Column chromatography using silica gel (eluting hexane) afforded the crude product. Then, the crude product reacted with potassium *te*rt-butoxide (1.12g, 10 mmol) in THF (20.00 mL) at RT for 10 min, Column chromatography using silica gel (eluting hexane) afforded the product **2a** (1.79 g, 91%) as colorless crystal. The compound has been reported.[12]
¹H NMR (400 MHz, $CDCl_3$) δ 7.61–7.56 (m, 4H), 7.46 (t, *J* = 7.6 Hz, 2H), 7.36 (t, *J* = 7.3 Hz, 1H), 7.11 (d, *J* = 8.6 Hz, 2H), 6.72 (dd, *J* = 13.7, 6.1 Hz, 1H), 4.84 (dd, *J* = 13.7, 1.3 Hz, 1H), 4.50 (dd, *J* = 6.0, 1.3 Hz, 1H).
¹³C NMR (101 MHz, $CDCl_3$) δ 156.3, 148.1, 140.5, 136.2, 128.7, 128.3, 127.0, 126.9, 117.3, 95.3.

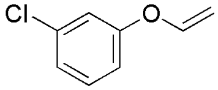

1-Chloro-3-vinyloxy-benzene. The titled product was prepared by following General Procedure B using 3-Chlorophenol (1.29 g, 10 mmol), K_2CO_3 (4.15 g, 30.00 mmol) and 1,2-Dibromoethane (2.59 mL, 30 mmol) in acetone (30.00 mL) and the mixture was refluxed for 12 h. Column chromatography using silica gel (eluting hexane) afforded the crude product. Then, the crude product reacted with potassium *tert*-butoxide (1.12g, 10 mmol) in THF (20.00 mL) at RT for 10 min, Column chromatography using silica gel (eluting hexane) afforded the product **2b** (1.29 g, 84%) as a colorless liquid. The compound has been reported.[13]

^{1}H NMR (400 MHz, $CDCl_3$) δ 7.27 (t, *J* = 7.6 Hz, 1H), 7.09 (d, *J* = 8.0 Hz, 1H), 7.04 (d, *J* = 1.4 Hz, 1H), 6.93 (d, *J* = 8.3 Hz, 1H), 6.63 (dd, *J* = 13.7, 6.0 Hz, 1H), 4.84 (d, *J* = 13.7 Hz, 1H), 4.53 (d, *J* = 6.0 Hz, 1H).

^{13}C NMR (101 MHz, $CDCl_3$) δ 157.4, 147.4, 135.0, 130.4, 123.2, 117.5, 115.3, 96.3.

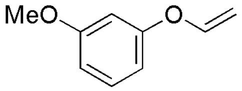

1-Methoxy-3-vinyloxy-benzene. The titled product was prepared by following General Procedure B using 3-Methoxyphenol (1.24 g, 10 mmol), K_2CO_3 (4.15 g, 30.00 mmol) and 1,2-Dibromoethane (3.05 mL, 30mmol) in acetone (30.00 mL) and the mixture was refluxed for 12 h. Column chromatography using silica gel (eluting hexane) afforded the crude product. Then, the crude product reacted with pmin, Column chromatography using silica gel (eluting with 5% EtOAc/hexane) afforded the product **2c** (1.29 g, 86%) as a colorless liquid. The compound has been reported.[13]

^{1}H NMR (400 MHz, $CDCl_3$) δ 7.24 (t, *J* = 8.2 Hz, 1H), 6.71–6.61 (m, 3H), 6.59 (s, 1H), 4.81 (dd, *J* = 13.7, 1.2 Hz, 1H), 4.46 (dd, *J* = 6.0, 1.2 Hz, 1H), 3.84 (d, *J* = 14.7 Hz, 3H).

^{13}C NMR (101 MHz, $CDCl_3$) δ 160.8, 158.0, 147.9, 130.0, 109.0, 108.8, 103.2, 95.2, 55.3.

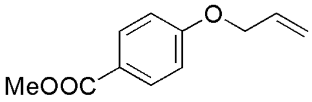

Methyl 4-allyloxybenzoate. The titled product was prepared by following General Procedure A using methyl 4-hydroxy-benzoate (1.52 g, 10 mmol), K_2CO_3 (4.15 g, 30.00 mmol) and Allyl bromide (1.04 mL, 12mmol) in acetone (30.00 mL) and the mixture was refluxed for 12 h. Column chromatography using silica gel (eluting with 5% EtOAc/hexane) afforded the product **2d** (1.90 g, 99%) as a colorless liquid. The compound has been reported.[14]

^{1}H NMR (400 MHz, $CDCl_3$) δ 7.99 (d, *J* = 8.7 Hz, 2H), 6.93 (d, *J* = 8.7 Hz, 2H), 6.05 (ddd, *J* = 22.3, 10.5, 5.2 Hz, 1H), 5.43 (d, *J* = 17.3 Hz, 1H), 5.31 (d, *J* = 10.5 Hz, 1H), 4.58 (d, *J* = 5.1 Hz, 2H), 3.88 (s, 3H).

^{13}C NMR (101 MHz, $CDCl_3$) δ 166.8, 162.3, 132.5, 131.5, 122.7, 118.0, 114.3, 68.8, 51.8.

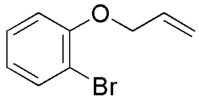

2-(allyloxy)bromobenzene. The titled product was prepared by following General Procedure A using 2-Bromophenol (1.73 g, 10 mmol), K_2CO_3 (4.15 g, 30.00 mmol) and Allyl bromide (1.04 mL, 12mmol) in acetone (30.00 mL) and the mixture was refluxed for 12 h. Column

chromatography using silica gel (eluting with hexane) afforded the product **2e** (1.98 g, 93%) as a colorless liquid. The compound has been reported.[15]

^{1}H NMR (400 MHz, $CDCl_3$) δ 7.57 (d, *J* = 7.8 Hz, 1H), 7.26 (d, *J* = 7.6 Hz, 1H), 6.92 (d, *J* = 8.2 Hz, 1H), 6.86 (t, *J* = 7.6 Hz, 1H), 6.09 (ddd, *J* = 15.9, 10.2, 4.9 Hz, 1H), 5.51 (d, *J* = 17.2 Hz, 1H), 5.34 (d, *J* = 10.6 Hz, 1H), 4.16–4.01 (m, 2H), 2.63 (dd, *J* = 3.8, 2.6 Hz, 2H).

^{13}C NMR (101 MHz, $CDCl_3$) δ 154.9, 133.4, 132.6, 128.3, 122.0, 117.7, 113.6, 112.3, 69.6.

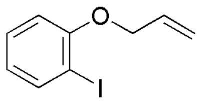

1-allyloxy-2-iodobenzene. The titled product was prepared by following General Procedure A using 2-iodophenol (2.20 g, 10 mmol), K_2CO_3 (4.15 g, 30.00 mmol) and Allyl bromide (1.04 mL, 12mmol) in acetone (30.00 mL) and the mixture was refluxed for 12 h. Column chromatography using silica gel (eluting with hexane) afforded the product **2f** (2.24 g, 86%) as a colorless liquid. The compound has been reported.[16]

^{1}H NMR (400 MHz, $CDCl_3$) δ 7.81 (d, *J* = 7.7 Hz, 1H), 7.31 (t, *J* = 8.3 Hz, 1H), 6.84 (d, *J* = 8.2 Hz, 1H), 6.74 (t, *J* = 7.5 Hz, 1H), 6.31–5.84 (m, 1H), 5.55 (d, *J* = 17.2 Hz, 1H), 5.34 (d, *J* = 10.6 Hz, 1H), 4.63 (d, *J* = 4.2 Hz, 2H).

^{13}C NMR (101 MHz, $CDCl_3$) δ 157.1, 139.5, 132.6, 129.3, 122.6, 117.6, 112.5, 86.7, 69.7.

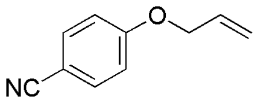

4-allyloxy-benzonitrile. The titled product was prepared by following General Procedure A using 4-Cyanophenol (1.19 g, 10 mmol), K_2CO_3 (4.15 g, 30.00 mmol) and Allyl bromide (1.04 mL, 12mmol) in acetone (30.00 mL) and the mixture was refluxed for 12 h. Column chromatography using silica gel (eluting with 5% EtOAc/hexane) afforded the product **2g** (1.58 g, 99%) as a colorless liquid. The compound has been reported.[17]

^{1}H NMR (400 MHz, $CDCl_3$) δ 7.60 (d, *J* = 8.5 Hz, 2H), 6.98 (d, *J* = 8.6 Hz, 2H), 6.05 (ddd, *J* = 16.2, 10.4, 5.2 Hz, 1H), 5.40 (dd, *J* = 34.7, 13.9 Hz, 2H), 4.61 (d, *J* = 5.0 Hz, 2H).

^{13}C NMR (101 MHz, $CDCl_3$) δ 161.8, 133.9, 132.0, 119.1, 118.4, 115.4, 104.1, 69.0.

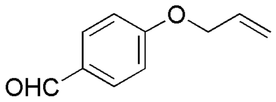

4-allyloxybenzaldehyde. The titled product was prepared by following General Procedure A using p-Hydroxybenzaldehyde (1.22 g, 10 mmol), K_2CO_3 (4.15 g, 30.00 mmol) and Allyl bromide (1.04 mL, 12mmol) in acetone (30.00 mL) and the mixture was refluxed for 12 h. Column chromatography using silica gel (eluting with 5% EtOAc/hexane) afforded the product **2h** (1.52 g, 94%) as a colorless liquid. The compound has been reported.[18]

^{1}H NMR (400 MHz, $CDCl_3$) δ 9.90 (s, 1H), 7.85 (d, *J* = 8.5 Hz, 2H), 7.04 (d, *J* = 8.5 Hz, 2H), 6.07 (ddd, *J* = 16.4, 10.5, 5.2 Hz, 1H), 5.45 (d, *J* = 17.3 Hz, 1H), 5.35 (d, *J* = 10.5 Hz, 1H), 4.65 (d, *J* = 5.0 Hz, 2H).

^{13}C NMR (101 MHz, $CDCl_3$) δ 190.7, 163.6, 132.3, 131.9, 130.0, 118.3, 115.0, 69.0.

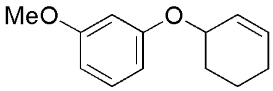

4-allyloxybenzaldehyde. The titled product was prepared by following General Procedure A using 3-Methoxyphenol (1.24 g, 10 mmol), K_2CO_3 (4.15 g, 30.00 mmol) and 3-Bromocyclohexene (1.15 mL, 12 mmol) in acetone (30.00 mL) and the mixture was refluxed for 12 h. Column chromatography using silica gel (eluting with 5% EtOAc/hexane) afforded the product **2i** (1.84 g, 91%) as a colorless liquid. The compound has been reported.[19]

^{1}H NMR (400 MHz, $CDCl_3$) δ 7.20 (t, *J* = 8.5 Hz, 1H), 6.55 (m, 3H), 6.00 (d, *J* = 10.1 Hz, 1H), 5.90 (d, *J* = 10.3 Hz, 1H), 4.81 (s, 1H), 3.81 (s, 3H), 2.13 (dd, *J* = 23.4, 21.1 Hz, 2H), 1.98–1.81 (m, 3H), 1.68 (dt, *J* = 9.6, 7.8 Hz, 1H).

^{3}C NMR (101 MHz, $CDCl_3$) δ 160.9, 159.1, 132.1, 129.8, 126.3, 107.8, 106.3, 102.3, 70.8, 55.2, 28.3, 25.1, 19.0.

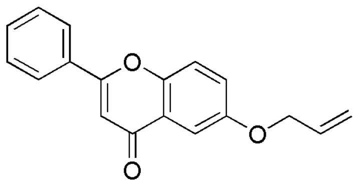

6-Allyloxy-2-phenyl-chromen-4-one. The titled product was prepared by following General Procedure A using 6-Hydroxyelavone (2.38 g, 10 mmol), K_2CO_3 (4.15 g, 30.00 mmol) and Allyl bromide (1.04 mL, 12 mmol) in acetone (30.00 mL) and the mixture was refluxed for 12 h. Column chromatography using silica gel (eluting with 5% EtOAc/hexane) afforded the product **2j** (2.42 g, 87%) as a colorless crystals. HRMS (ESI) calcd for $C_{18}H_{15}O_3^+[(M+H)^+]$ 279.1016, found 279.1023.

^{1}H NMR (400 MHz, DMSO) δ 8.10 (d, *J* = 6.4 Hz, 2H), 7.76 (d, *J* = 9.3 Hz, 1H), 7.59 (m, 3H), 7.46 (d, *J* = 7.7 Hz, 2H), 7.02 (s, 1H), 6.08 (ddd, *J* = 15.8, 10.3, 5.0 Hz, 1H), 5.44 (d, *J* = 17.2 Hz, 1H), 5.30 (d, *J* = 10.5 Hz, 1H), 4.69 (d, *J* = 4.7 Hz, 2H).

^{13}C NMR (101 MHz, DMSO) δ 177.3, 162.8, 155.9, 150.8, 133.7, 132.1, 131.6, 129.5, 126.7, 124.4, 124.0, 120.6, 118.1, 106.6, 106.4, 69.2.

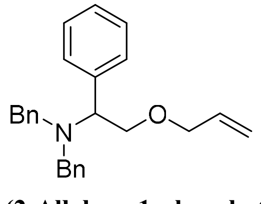

(2-Allyloxy-1-phenyl-ethyl)-diphenethyl-amine. The titled product was prepared by following General Procedure A using 2-Diphenethylamino-2-phenyl-ethanol (3.45 g, 10 mmol), NaH (0.288 g, 30.00 mmol) and Allyl bromide (1.04 mL, 12 mmol) in THF (30.00 mL) and the mixture was refluxed for 12 h. Column chromatography using silica gel (eluting with 20% EtOAc/hexane) afforded the product **2k** (3.73 g, 97%) as a colorless crystals. HRMS (ESI) calcd for $C_{25}H_{28}NO^+[(M+H)^+]$ 358.2165, found 358.2158.

^{1}H NMR (400 MHz, $CDCl_3$) δ 7.47–7.31 (m, 13H), 7.25 (t, *J* = 7.2 Hz, 2H), 5.92 (ddt, *J* = 16.2, 10.7, 5.5 Hz, 1H), 5.24 (dd, *J* = 32.1, 13.8 Hz, 2H), 4.05 (dt, *J* = 16.2, 6.4 Hz, 2H), 3.98 (d, *J* = 5.2 Hz, 2H), 3.93–3.88 (m, 1H), 3.85 (d, *J* = 14.1 Hz, 2H), 3.48 (d, *J* = 13.9 Hz, 2H).
^{13}C NMR (101 MHz, $CDCl_3$) δ 140.2, 138.7, 134.9, 128.7, 128.7, 128.2, 128.0, 127.1, 126.8, 116.8, 71.9, 70.1, 61.0, 54.4.

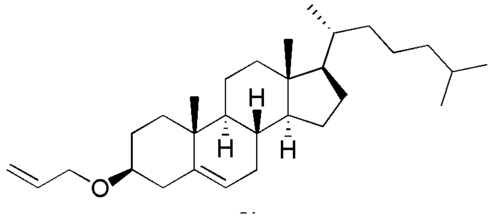

allyl cholesteryl ether. The titled product was prepared by following General Procedure A using Cholesterol (3.86 g, 10 mmol), NaH (0.288 g, 30.00 mmol) and Allyl bromide (1.04 mL, 12mmol) in THF (30.00 mL) and the mixture was refluxed for 12 h. Column chromatography using silica gel (eluting with hexane) afforded the product **2l** (3.97 g, 93%) as a colorless crystals. HRMS (ESI) calcd for $C_{30}H_{51}O^{+}[(M+H)^{+}]$ 427.3934, found 427.3928.
^{1}H NMR (400 MHz, $CDCl_3$) δ 5.95 (ddt, *J* = 16.2, 10.8, 5.6 Hz, 1H), 5.37 (d, *J* = 4.5 Hz, 1H), 5.29 (d, *J* = 17.2 Hz, 1H), 5.17 (d, *J* = 10.3 Hz, 1H), 4.04 (d, *J* = 5.5 Hz, 2H), 3.22 (dt, *J* = 15.4, 5.5 Hz, 1H), 2.39 (dd, *J* = 13.0, 2.6 Hz, 1H), 2.25 (t, *J* = 11.6 Hz, 1H), 2.09–1.78 (m, 5H), 1.60–0.82 (m, 33H), 0.70 (s, 3H).
^{13}C NMR (101 MHz, $CDCl_3$) δ 141.0, 135.5, 121.5, 116.4, 78.5, 69.0, 56.8, 56.1, 50.2, 42.3, 39.8, 39.5, 39.1, 37.2, 36.9, 36.2, 35.8, 31.9, 31.9, 28.4, 28.2, 28.0, 24.3, 23.8, 22.8, 22.5, 21.0, 19.3, 18.7, 11.8.

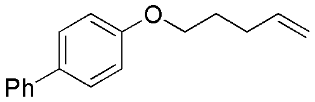

5-(4-biphenylphenoxy)-1-pentene. The titled product was prepared by following General Procedure A using 4-Phenylphenol (1.70 g, 10 mmol), K_2CO_3 (4.15 g, 30.00 mmol) and 5-Bromo-1-pentene (1.18 mL, 12mmol) in acetone (30.00 mL) and the mixture was refluxed for 12 h. Column chromatography using silica gel (eluting with hexane) afforded the product **2m** (1.84 g, 77%) as a colorless crystals. The compound has been reported.[20]
^{1}H NMR (400 MHz, $CDCl_3$) δ 7.57 (m, 4H), 7.45 (t, *J* = 7.6 Hz, 2H), 7.34 (t, *J* = 7.3 Hz, 1H), 7.01 (d, *J* = 8.7 Hz, 2H), 5.91 (ddt, *J* = 16.9, 10.2, 6.7 Hz, 1H), 5.29–4.79 (m, 2H), 4.05 (t, *J* = 6.4 Hz, 2H), 2.30 (dd, *J* = 14.3, 7.1 Hz, 2H), 2.00–1.90 (m, 2H).
^{13}C NMR (101 MHz, $CDCl_3$) δ 158.6, 140.9, 137.8, 133.6, 128.7, 128.4, 126.7, 126.6, 115.2, 114.8, 67.2, 30.1, 28.4.

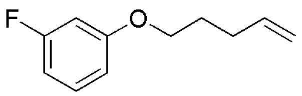

1-fluoro-4-(4-pentenyloxy)benzene. The titled product was prepared by following General Procedure A using 3-Fluorophenol (1.12 g, 10 mmol), K_2CO_3 (4.15 g, 30.00 mmol) and

5-Bromo-1-pentene (1.18 mL, 12mmol) in acetone (30.00 mL) and the mixture was refluxed for 12 h. Column chromatography using silica gel (eluting with hexane) afforded the product **2n** (1.46 g, 81%) as a colorless liquid. HRMS (ESI) calcd for $C_{11}H_{14}FO^+[(M+H)^+]$ 181.1023, found 181.1032.

^{1}H NMR (400 MHz, $CDCl_3$) δ 7.23 (dd, *J* = 15.4, 7.9 Hz, 1H), 6.79–6.56 (m, 3H), 5.95–5.82 (m, 1H), 5.07 (dd, *J* = 22.3, 13.7 Hz, 2H), 3.98 (t, *J* = 6.4 Hz, 2H), 2.27 (q, *J* = 7.1 Hz, 2H), 1.95–1.87 (m, 2H).

^{13}C NMR (101 MHz, $CDCl_3$) δ 163.6 (d, *J* = 244.8 Hz), 160.4 (d, *J* = 10.9 Hz), 137.6, 130.1 (d, *J* = 10.1 Hz), 115.3, 110.3, 107.3 (d, *J* = 21.4 Hz), 102.1 (d, *J* = 24.6 Hz), 67.4, 30.0, 28.3.

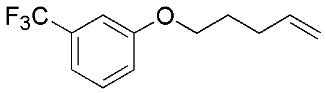

5-(4-biphenylphenoxy)-1-pentene. The titled product was prepared by following General Procedure A using 3-Trifluoromethylphenol (1.62 g, 10 mmol), K_2CO_3 (4.15 g, 30.00 mmol) and 5-Bromo-1-pentene (1.18 mL, 12mmol) in acetone (30.00 mL) and the mixture was refluxed for 12 h. Column chromatography using silica gel (eluting with hexane) afforded the product **2o** (1.57 g, 68%) as a colorless liquid. HRMS (ESI) calcd for $C_{12}H_{14}F_3O^+[(M+H)^+]$ 231.0991, found 231.0985.

^{1}H NMR (400 MHz, $CDCl_3$) δ 7.40 (t, *J* = 8.0 Hz, 1H), 7.23 (d, *J* = 7.7 Hz, 1H), 7.16 (s, 1H), 7.09 (d, *J* = 8.3 Hz, 1H), 5.89 (ddt, *J* = 16.9, 10.2, 6.7 Hz, 1H), 5.09 (dd, *J* = 22.2, 13.7 Hz, 2H), 4.04 (t, *J* = 6.4 Hz, 2H), 2.29 (dd, *J* = 14.2, 7.1 Hz, 2H), 1.95 (dd, *J* = 13.9, 6.8 Hz, 2H).

^{13}C NMR (101 MHz, Chloroform-*d*) δ 159.2, 137.5, 131.8 (q, *J* = 32.2 Hz), 129.9, 124.0 (q, *J* = 272.3 Hz), 117.9 (d, *J* = 1.5 Hz), 117.2 (q, *J* = 3.9 Hz), 115.3, 111.2 (q, *J* = 3.8 Hz), 67.4, 30.0, 28.3.

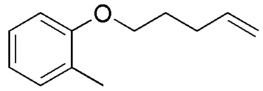

5-(2-Methylphenoxy)-1-pentene. The titled product was prepared by following General Procedure A using 4-Phenylphenol (1.70 g, 10 mmol), K_2CO_3 (4.15 g, 30.00 mmol) and 5-Bromo-1-pentene (1.18 mL, 12 mmol) in acetone (30.00 mL) and the mixture was refluxed for 12 h. Column chromatography using silica gel (eluting with hexane) afforded the product **2p** (1.22 g, 69%) as a a colorless liquid. . The compound has been reported.[20]

^{1}H NMR (400 MHz, $CDCl_3$) δ 7.19 (t, *J* = 6.6 Hz, 2H), 6.88 (dd, *J* = 19.7, 7.9 Hz, 2H), 6.03–5.80 (m, 1H), 5.09 (dd, *J* = 26.2, 13.7 Hz, 2H), 4.03 (t, *J* = 6.3 Hz, 2H), 2.37–2.26 (m, 5H), 1.99–1.91 (m, 2H).

^{13}C NMR (101 MHz, $CDCl_3$) δ 157.1, 138.0, 130.6, 126.8, 126.7, 120.1, 115.0, 110.9, 67.0, 30.2, 28.6, 16.2.

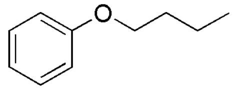

Butoxybenzene (8). The titled product was prepared by following General Procedure A using Phenol (0.94 g, 10 mmol), K_2CO_3 (4.15 g, 30.00 mmol) and 1-Bromobutane (1.61 mL, 15 mmol)

in acetone (30.00 mL) and the mixture was refluxed for 12 h. Column chromatography using silica gel (eluting with hexane) afforded the product **2p** (1.49 g, 99%) as a a colorless liquid. The compound has been reported.[20]

^{1}H NMR (400 MHz, $CDCl_3$) δ 7.37 (t, *J* = 7.8 Hz, 2H), 7.01 (dd, *J* = 14.0, 7.6 Hz, 3H), 4.04 (t, *J* = 6.5 Hz, 2H), 1.93–1.81 (m, 2H), 1.65–1.53 (m, 2H), 1.08 (t, *J* = 7.4 Hz, 3H).

^{13}C NMR (101 MHz, $CDCl_3$) δ 159.2, 129.4, 120.5, 114.5, 67.5, 31.4, 19.3, 13.9.

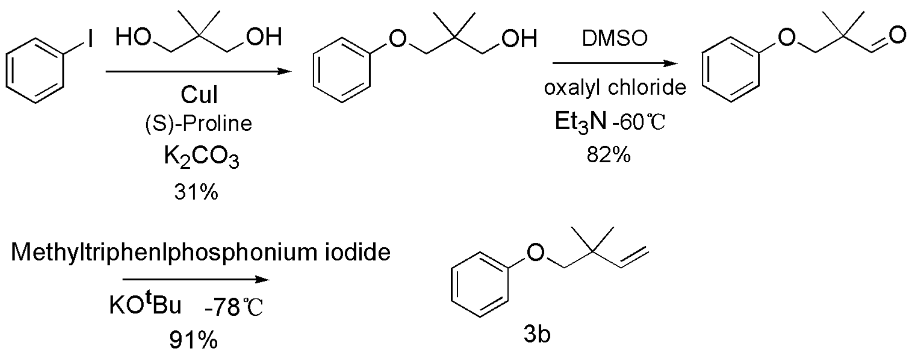

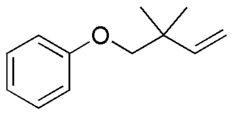

((2,2-dimethylbut-3-en-1-yl)oxy)benzene (9). HRMS (ESI) calcd for $C_{12}H_{17}O$+[(M+H)$^+$] 177.1274, found 177.1269.

^{1}H NMR (400 MHz, $CDCl_3$) δ 7.31-7.30 (m, 2H), 6.94 (dd, *J* = 13.9, 7.6 Hz, 3H), 5.98 (dd, *J* = 17.5, 10.8 Hz, 1H), 5.08 (dd, *J* = 19.1, 14.2 Hz, 2H), 3.71 (s, 2H), 1.18 (s, 6H).

^{13}C NMR (101 MHz, $CDCl_3$) δ 159.4, 145.2, 129.4, 120.6, 114.6, 112.1, 76.3, 37.8, 24.1.

3. Mechanistic research

In glovebox, B_2pin_2 (76.17 mg, 0.3 mmol, 1.5 equiv), $NiCl_2$(DME) (2.20 mg, 0.01 mmol, 0.05 equiv), 6,6-dimethyl-2,2-dipyridyl (3.68 mg, 0.02 mmol, 0.10 equiv), LiO*t*Bu (32 mg, 0.4 mmol, 2.0 equiv) were added to a oven-dried tube. The reaction tube was equipped with a magnetic stir bar and sealed with Teflon-lined cap, refilled the system with argon and performed two more evacuation-backfill cycles, butoxybenzene (0.2 mmol) was added by syringe under argon flow (if liquid). DMA (2.00 mL), H_2O (50 ul), MeOH (25 ul) were added to the tube sequentially. Aqueous HCl (0.1 M) was added to the mixture and extracted with CH_2Cl_2 (2 × 10 mL), dried over anhydrous $MgSO_4$, and determined by GC-MS, no phenol was detected.

In glovebox, B_2pin_2 (76.17 mg, 0.3 mmol, 1.5 equiv), $NiCl_2$(DME) (2.20 mg, 0.01 mmol, 0.05 equiv), 6,6-dimethyl-2,2-dipyridyl (3.68 mg, 0.02 mmol, 0.10 equiv), LiO*t*Bu (32 mg, 0.4 mmol, 2.0 equiv) were added to a oven-dried tube. The reaction tube was equipped with a magnetic stir bar and sealed with Teflon-lined cap, refilled the system with argon and performed two more evacuation-backfill cycles, ((2,2-dimethylbut-3-en-1-yl)oxy)benzene (0.2 mmol) was added by syringe under argon flow (if liquid). DMA (2.00 mL), H_2O (50 ul), MeOH (25 ul) were added to the

tube sequentially. Aqueous HCl (0.1 M) was added to the mixture and extracted with CH_2Cl_2 (2 × 10 mL), dried over anhydrous $MgSO_4$, and determined by GC-MS, no phenol was detected.

In glovebox, B_2pin_2 (76.17 mg, 0.3 mmol, 1.5 equiv), $NiCl_2$(DME) (2.20 mg, 0.01 mmol, 0.05 equiv), 6,6-dimethyl-2,2-dipyridyl (3.68 mg, 0.02 mmol, 0.10 equiv), LiO*t*Bu (32 mg, 0.4 mmol, 2.0 equiv) were added to a oven-dried tube. The reaction tube was equipped with a magnetic stir bar and sealed with Teflon-lined cap, refilled the system with argon and performed two more evacuation-backfill cycles, 1-(cinnamyloxy)-3-methoxybenzene (0.2 mmol) was added by syringe under argon flow (if liquid). DMA (2.00 mL), H_2O (50 ul), MeOH (25 ul) were added to the tube sequentially. Aqueous HCl (0.1 M) was added to the mixture and extracted with CH_2Cl_2 (2 × 10 mL), dried over anhydrous $MgSO_4$, and determined by GC-MS with mesitylene as internal standard. Allylbenzene was detected and confirmed by authentic sample.

All the products were all reported and compared with the previous reports. 4-Phenylphenol (2a),[21] Phenol (2b),[21] 3-Methoxyphenol (2c),[17] o-Cresol (2d),[21] 3-Fluorophenol (2e),[22] 3-Chlorophenol (2f),[22] 2-Iodophenol (2g),[23] 2-Bromophenol (2h),[24] Methyl 4-hydroxybenzoate (2i),[25] Ethyl 3-hydroxybenzoate (2j),[26] 4-tert-Butylphenol (2k),[27] 4-Hydroxybenzoic acid (2l),[17] 4-benzyloxyphenol (2m),[28] 2-Naphthol (2n),[21] p-Hydroxybenzaldehyde (2o),[21] 3-Trifluoromethylphenol (2p),[28] tert-butyl 4-hydroxy-1H-indole-1-carboxylate (2q),[29] 6-Hydroxyflavone (2r),[30] 4-Cyanophenol (2s),[21] 2-(N,N-dibenzylamino)-2-phenylethan-1-ol (2t),[31] Cholesterol (3u).[32]

4. Sepctrum

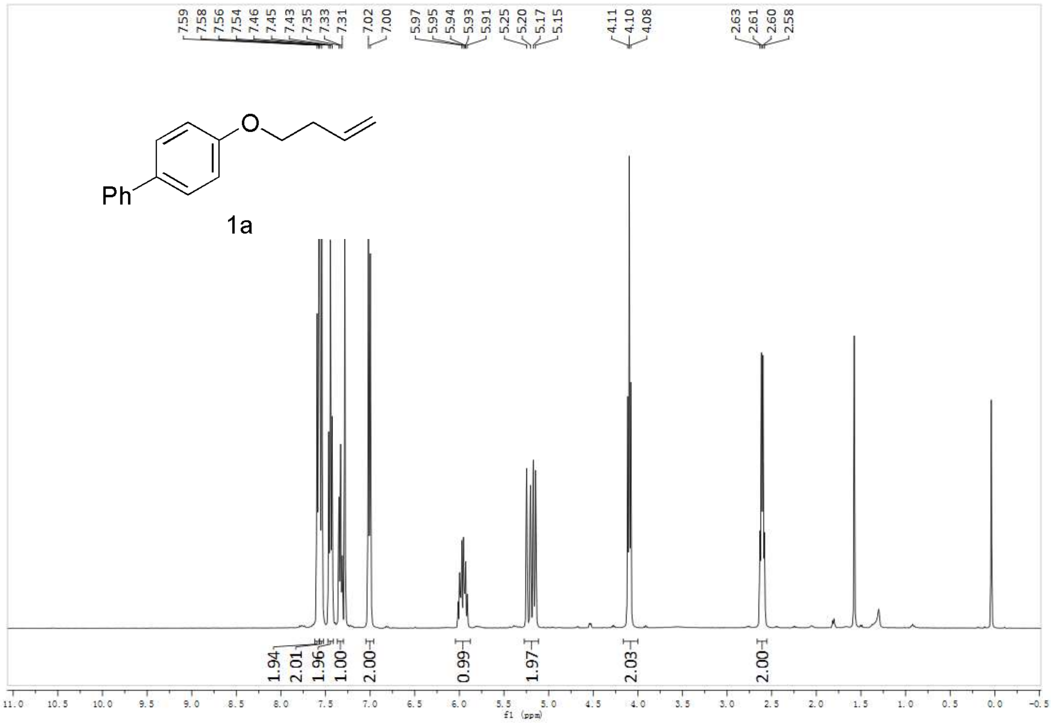
7.59
7.58
7.56
7.54
7.46
7.45
7.43
7.35
7.33
7.31
7.02
7.00
5.97
5.95
5.94
5.93
5.91
5.25
5.20
5.17
5.15
4.11
4.10
4.08
2.63
2.61
2.60
2.58
O
Ph
1a
1.94
2.01
1.96
1.00
2.00
0.99
1.97
2.03
2.00
f1 (ppm)

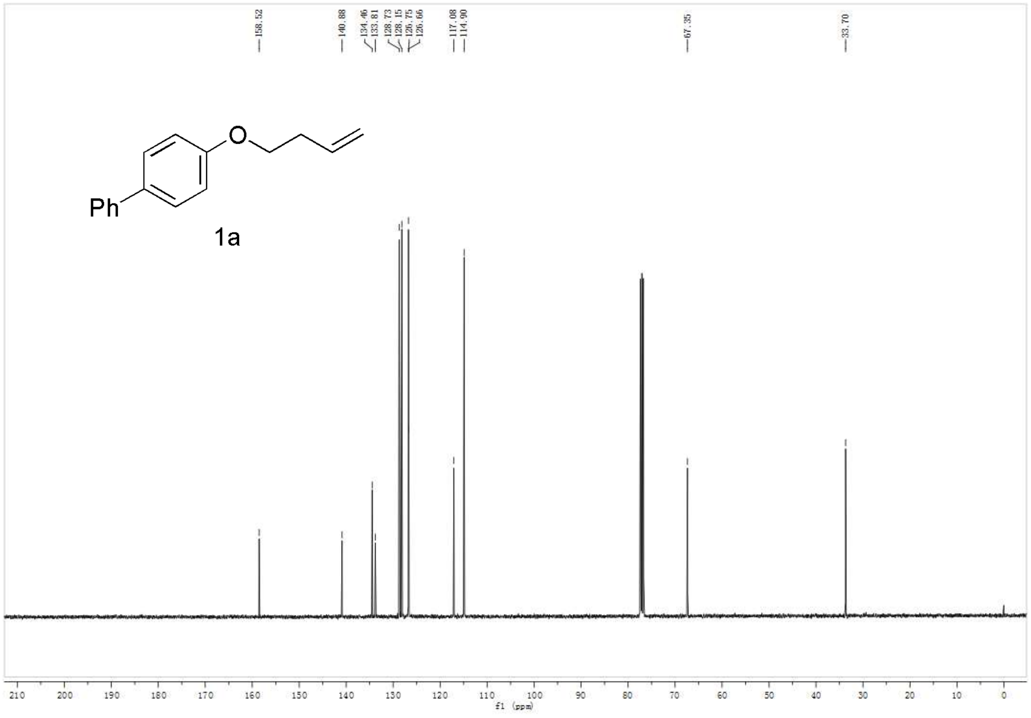
158.52
140.88
134.46
133.81
128.73
128.15
126.75
126.66
117.08
114.90
67.35
33.70
O
Ph
1a
f1 (ppm)

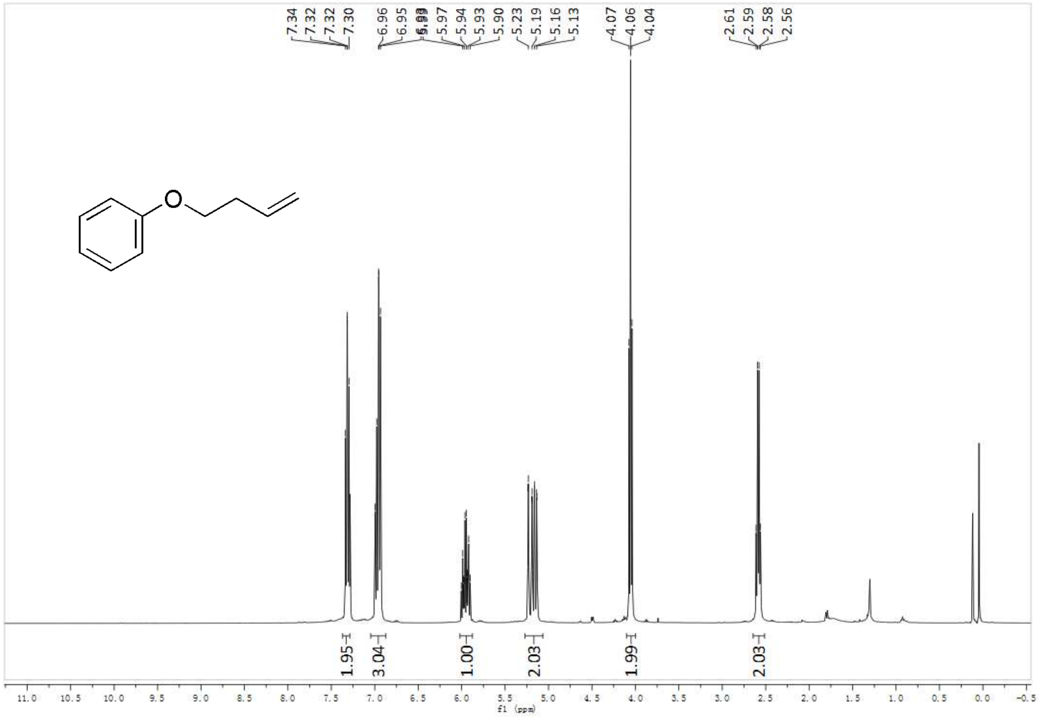

7.34
7.32
7.32
7.30
6.96
6.95
5.97
5.94
5.93
5.90
5.23
5.19
5.16
5.13
4.07
4.06
4.04
2.61
2.59
2.58
2.56
1.95
3.04
1.00
2.03
1.99
2.03
f1 (ppm)

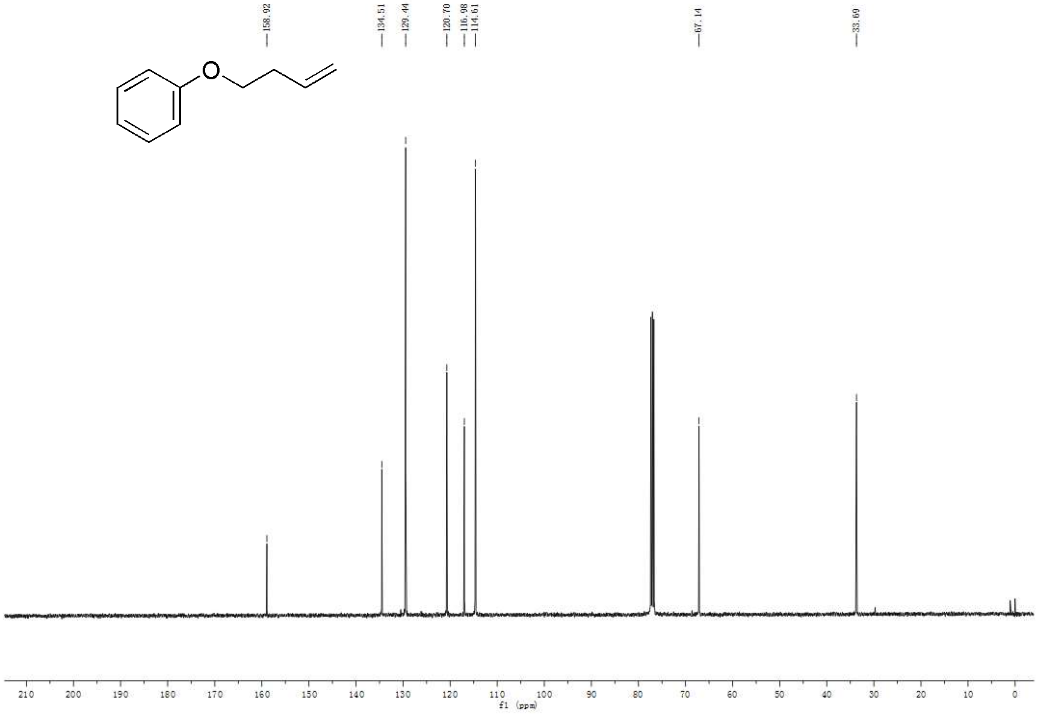

158.92
134.51
129.44
120.70
116.98
114.61
67.14
33.69
f1 (ppm)

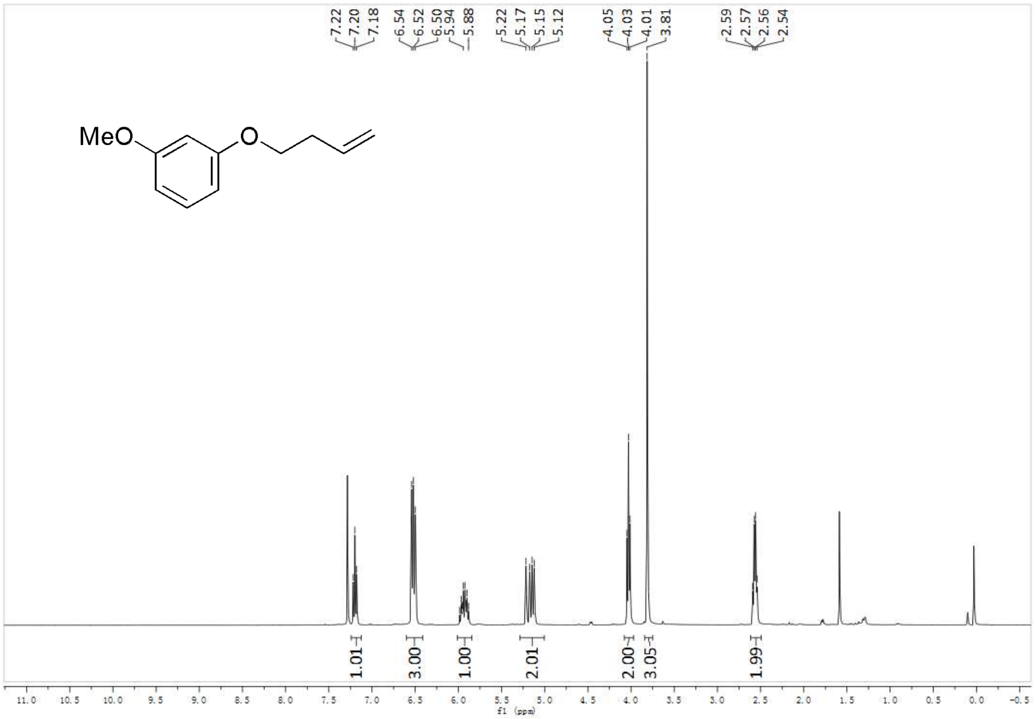

7.22
7.20
7.18
6.54
6.52
6.50
5.94
5.88
5.22
5.17
5.15
5.12
4.05
4.03
4.01
3.81
2.59
2.57
2.56
2.54
MeO
O
1.01
3.00
1.00
2.01
2.00
3.05
1.99
f1 (ppm)

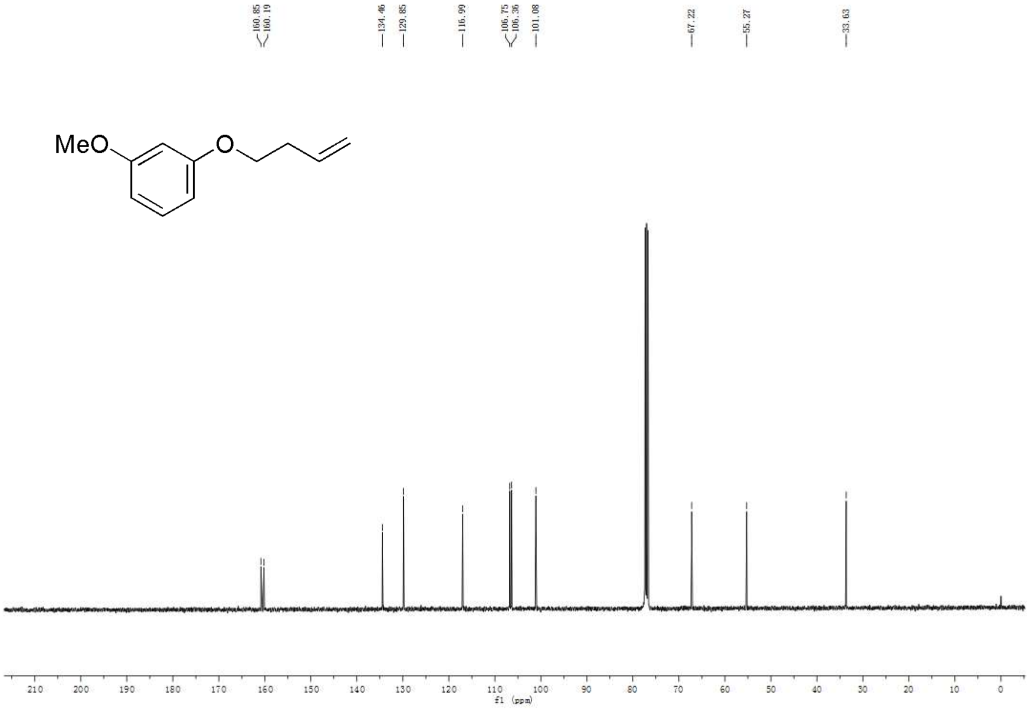

160.85
160.19
134.46
129.85
116.99
106.75
106.36
101.08
67.22
55.27
33.63
MeO
O
f1 (ppm)

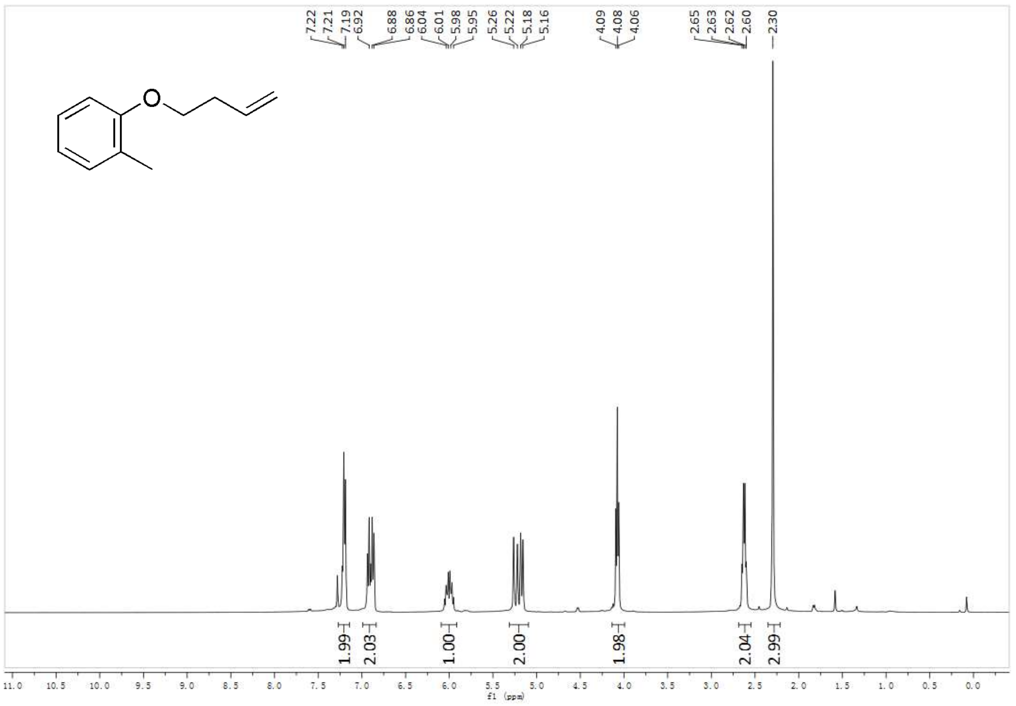
7.22
7.21
7.19
6.92
6.88
6.86
6.04
6.01
5.98
5.95
5.26
5.22
5.18
5.16
4.09
4.08
4.06
2.65
2.63
2.62
2.60
2.30
O
1.99
2.03
1.00
2.00
1.98
2.04
2.99
f1 (ppm)

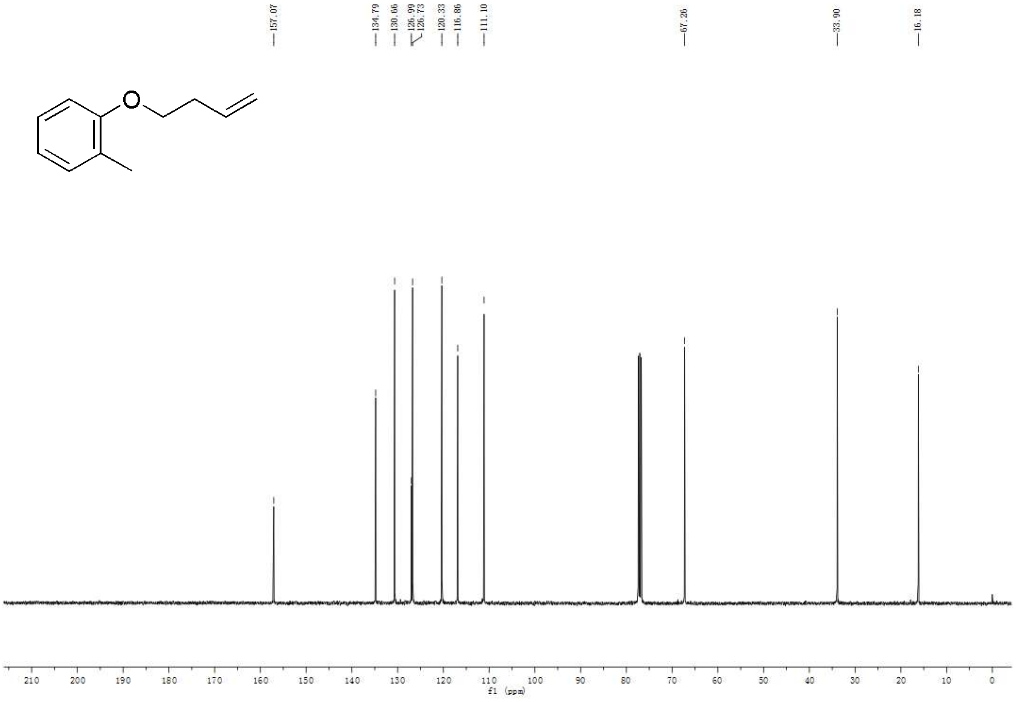
157.07
134.79
130.66
126.99
126.73
120.33
116.86
111.10
67.26
33.90
16.18
O
f1 (ppm)

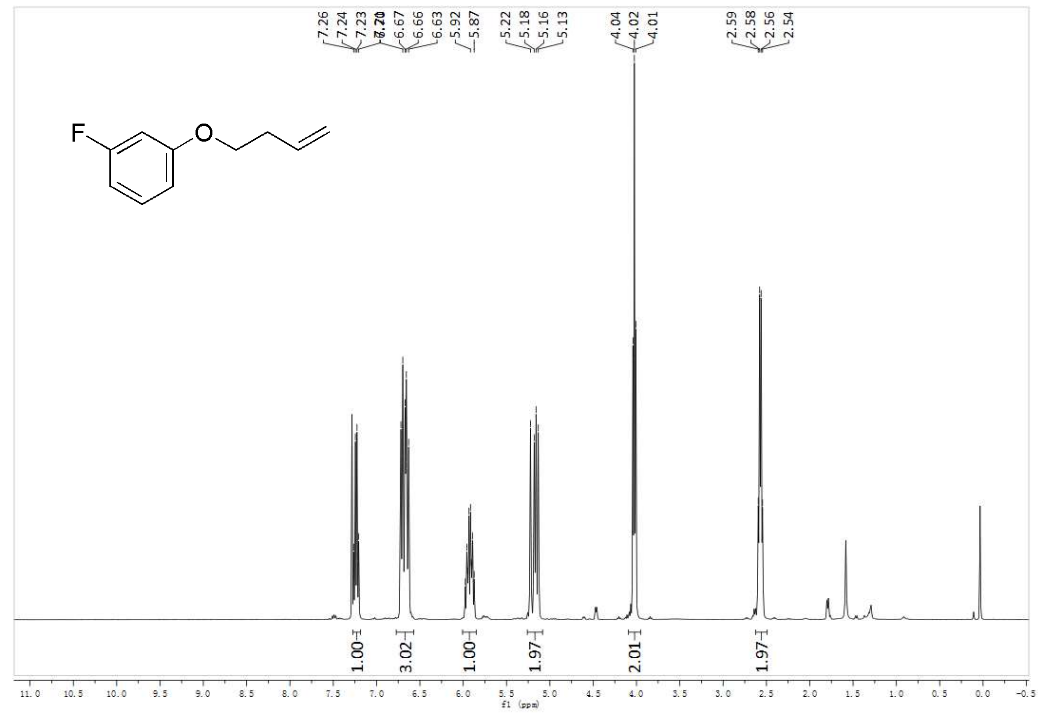
7.26
7.24
7.23
7.20
6.67
6.66
6.63
5.92
5.87
5.22
5.18
5.16
5.13
4.04
4.02
4.01
2.59
2.58
2.56
2.54
F
O
1.00
3.02
1.00
1.97
2.01
1.97
f1 (ppm)

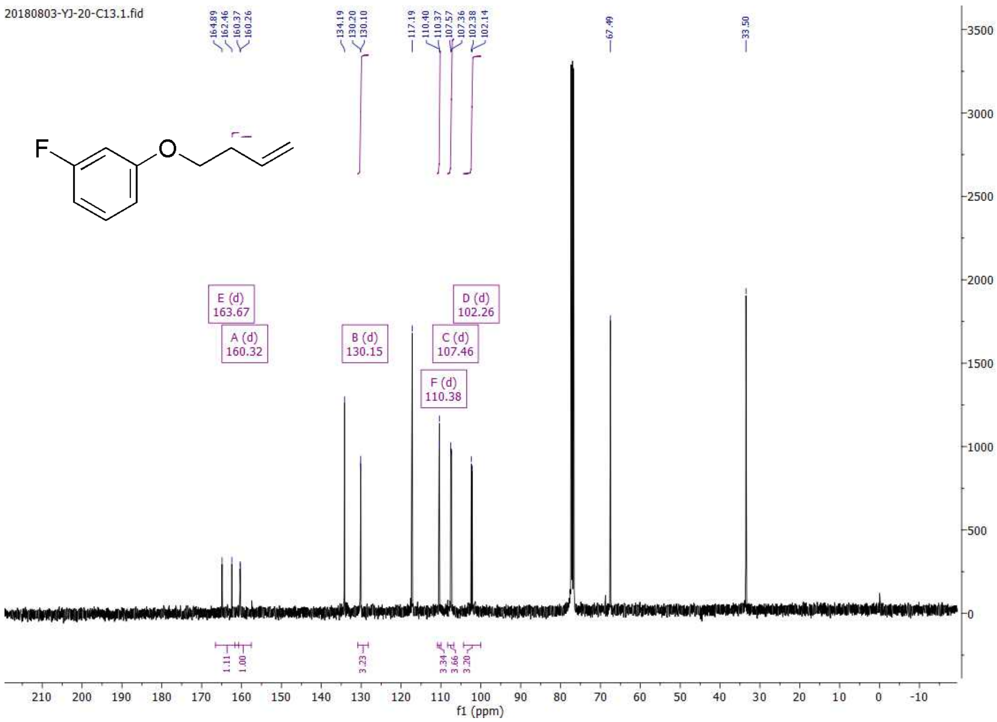
20180803-YJ-20-C13.1.fid
164.89
162.46
160.37
160.26
134.19
130.20
130.10
117.19
110.40
110.37
107.57
107.36
102.38
102.14
67.49
33.50
F
O
E (d)
163.67
A (d)
160.32
B (d)
130.15
D (d)
102.26
C (d)
107.46
F (d)
110.38
1.11
1.00
3.23
3.34
3.66
3.20
f1 (ppm)

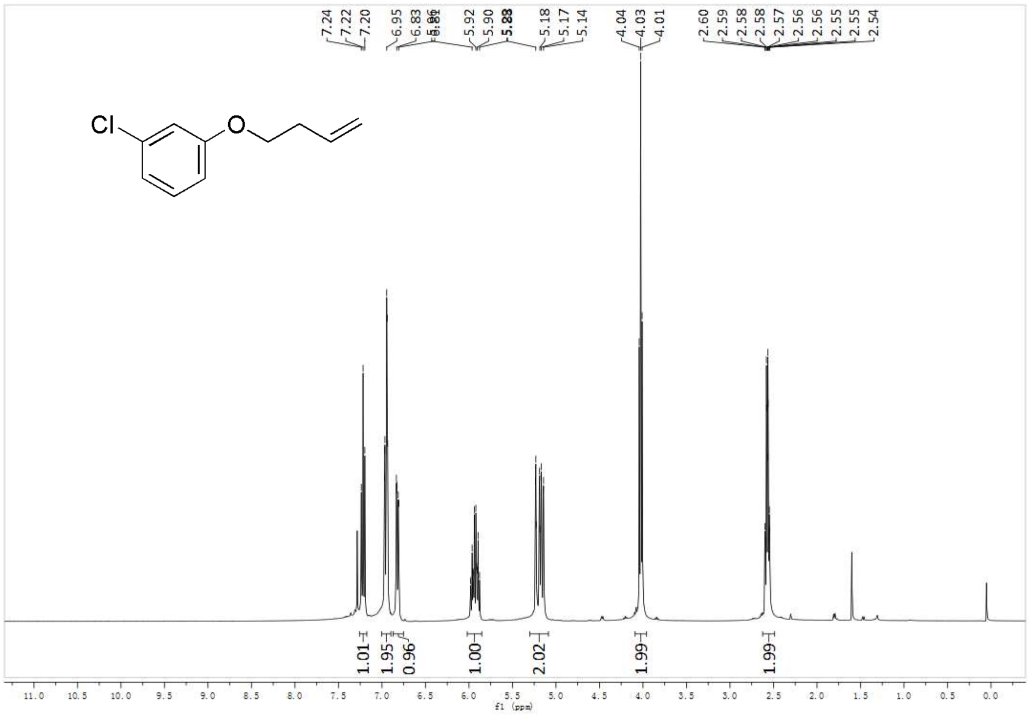

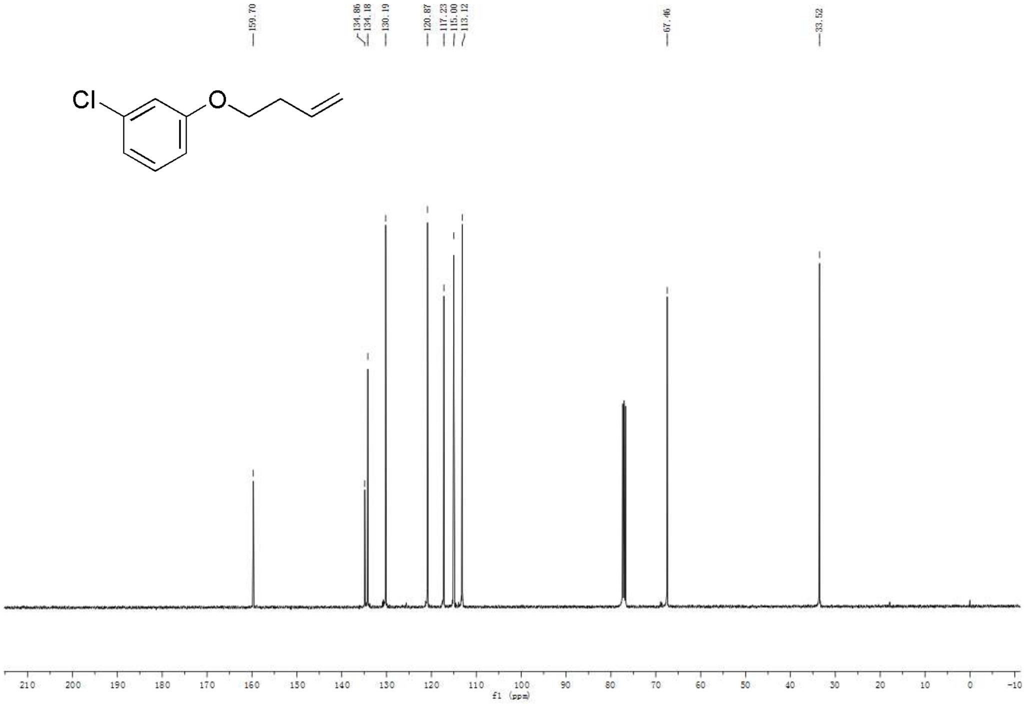

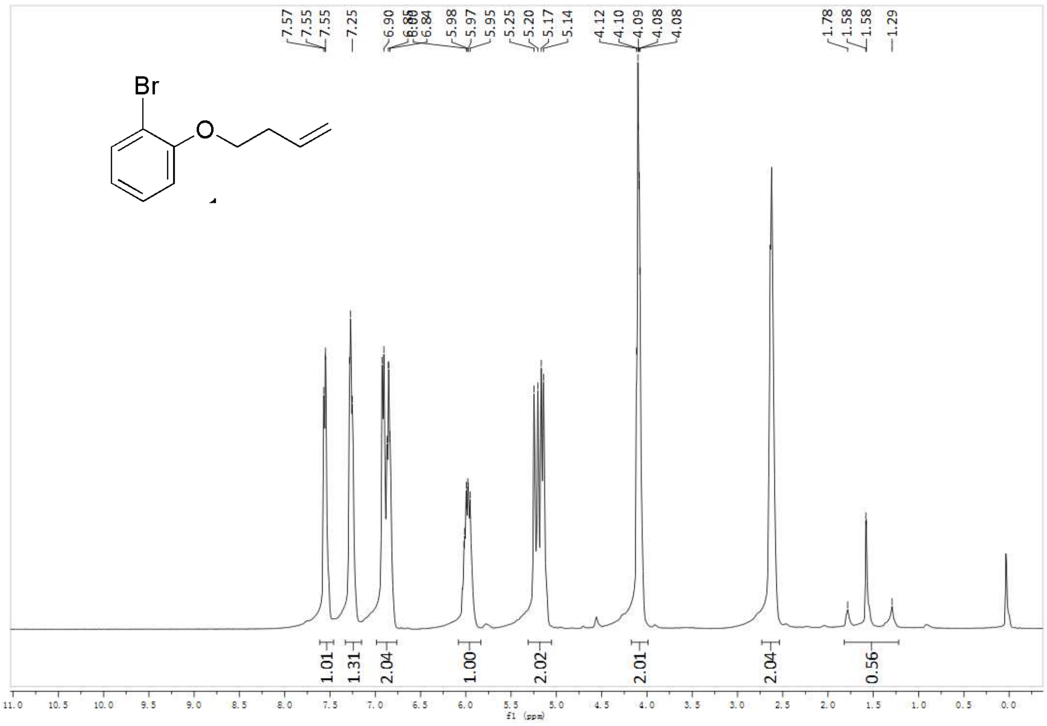
Br
O
7.57
7.55
7.55
7.25
6.90
6.84
5.98
5.97
5.95
5.25
5.20
5.17
5.14
4.12
4.10
4.09
4.08
4.08
1.78
1.58
1.58
1.29
1.01
1.31
2.04
1.00
2.02
2.01
2.04
0.56
f1 (ppm)

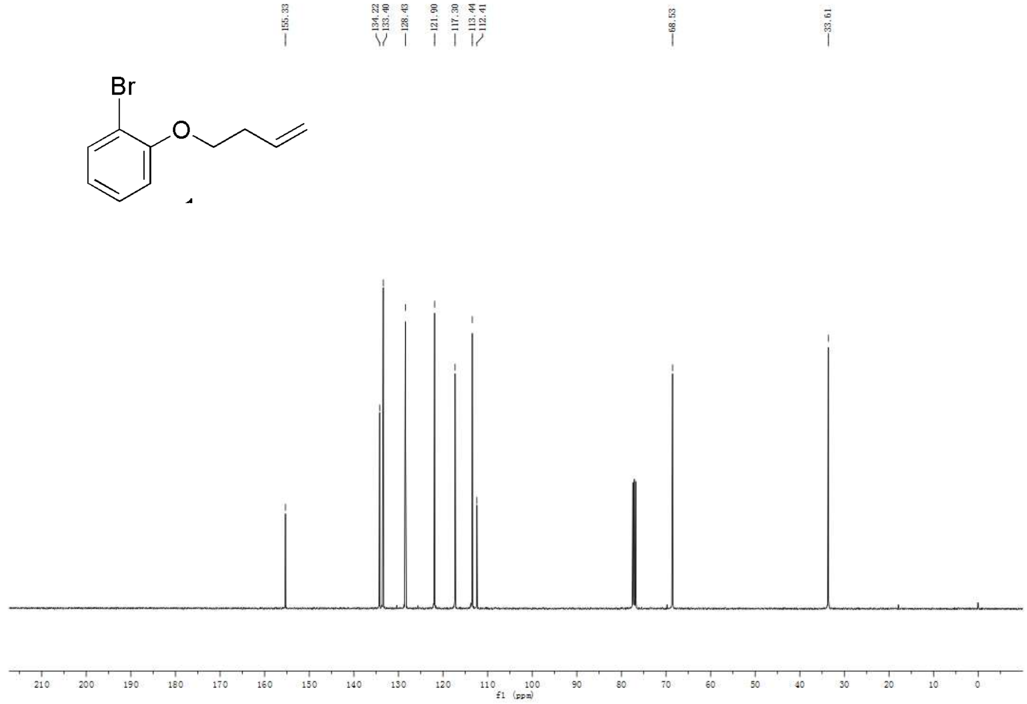
155.33
134.22
133.40
128.43
121.90
117.30
113.44
112.41
68.53
33.61
Br
O
f1 (ppm)

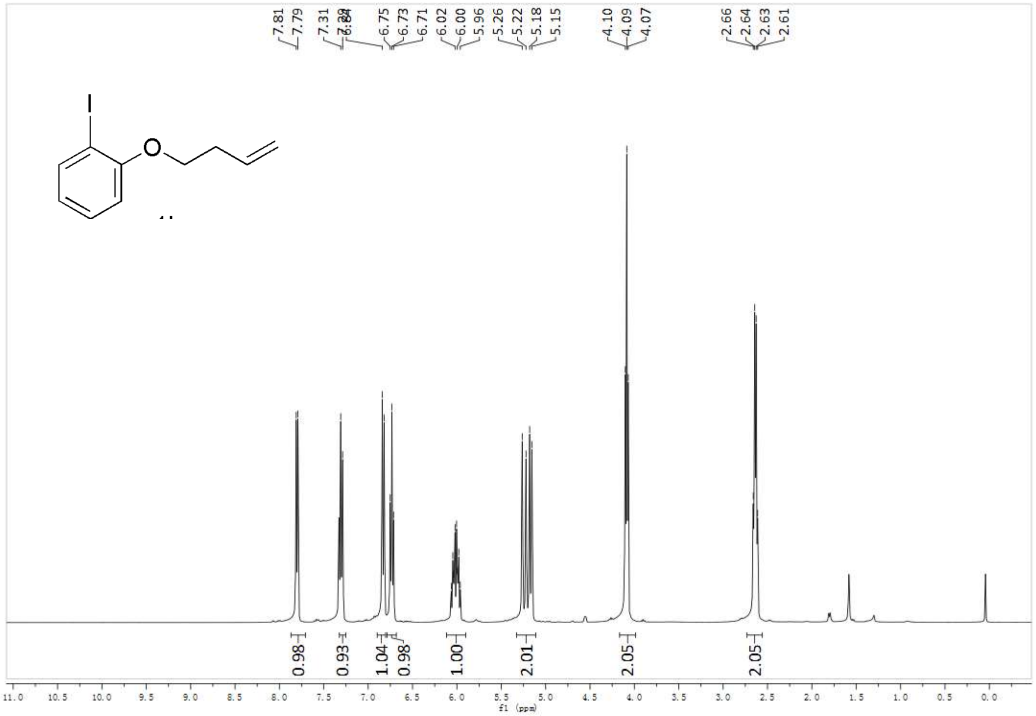

7.81
7.79
7.31
6.75
6.73
6.71
6.02
6.00
5.96
5.26
5.22
5.18
5.15
4.10
4.09
4.07
2.66
2.64
2.63
2.61
I
O
0.98
0.93
1.04
0.98
1.00
2.01
2.05
2.05
f1 (ppm)

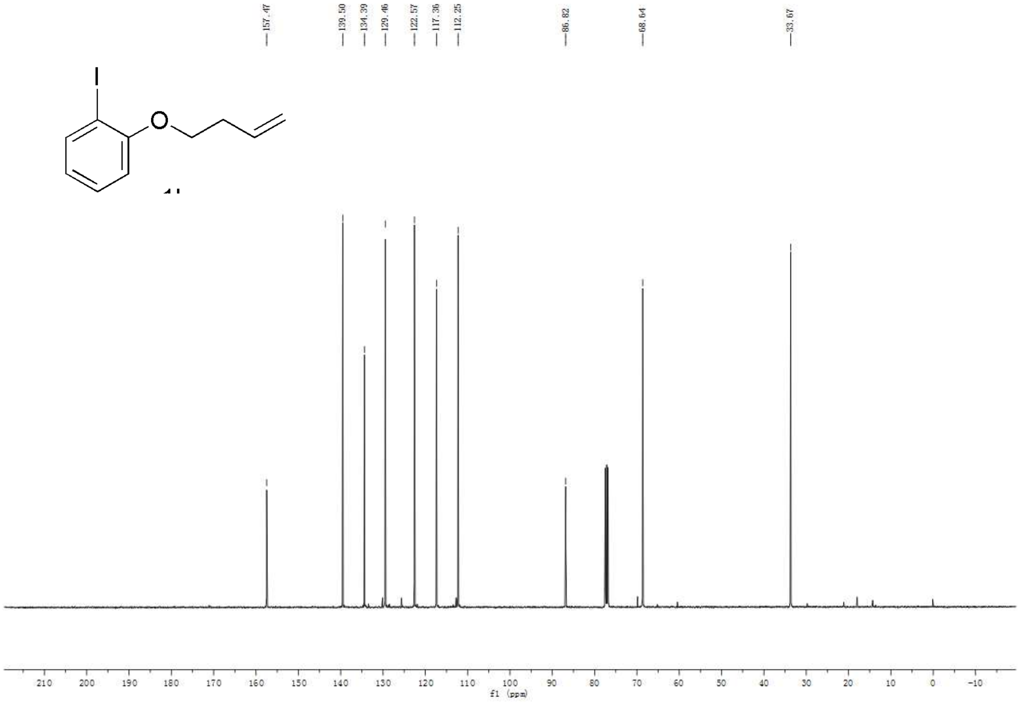

157.47
139.50
134.39
129.46
122.57
117.36
112.25
86.82
68.64
33.67
I
O
f1 (ppm)

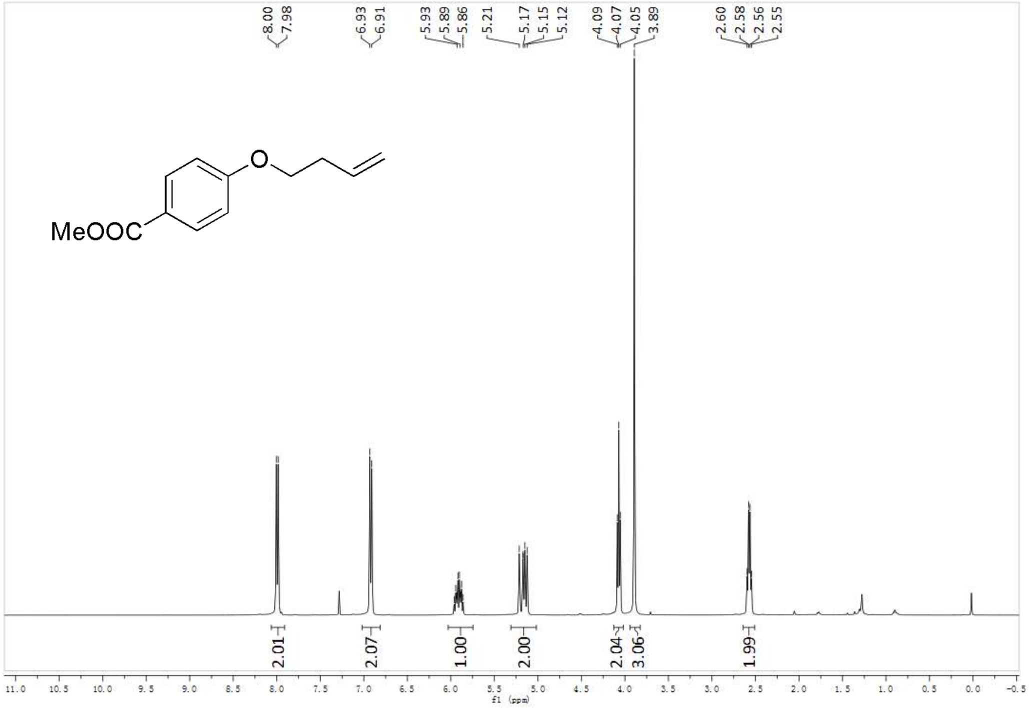

MeOOC
O
8.00
7.98
6.93
6.91
5.93
5.89
5.86
5.21
5.17
5.15
5.12
4.09
4.07
4.05
3.89
2.60
2.58
2.56
2.55
2.01
2.07
1.00
2.00
2.04
3.06
1.99
f1 (ppm)

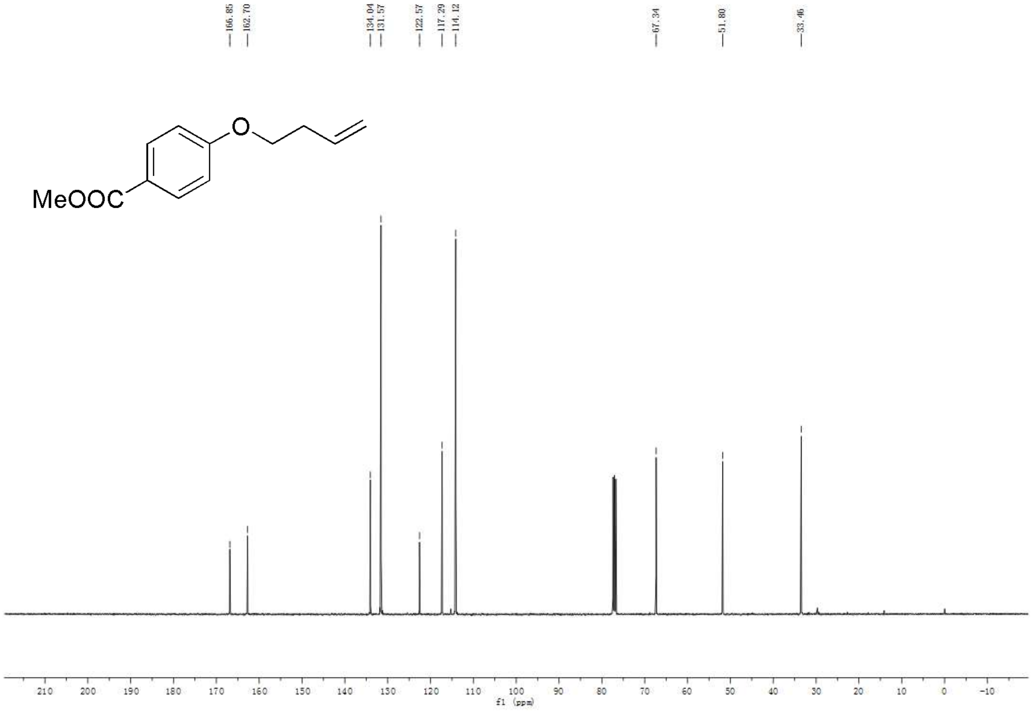

MeOOC
O
166.85
162.70
134.04
131.57
122.57
117.29
114.12
67.34
51.80
33.46
f1 (ppm)

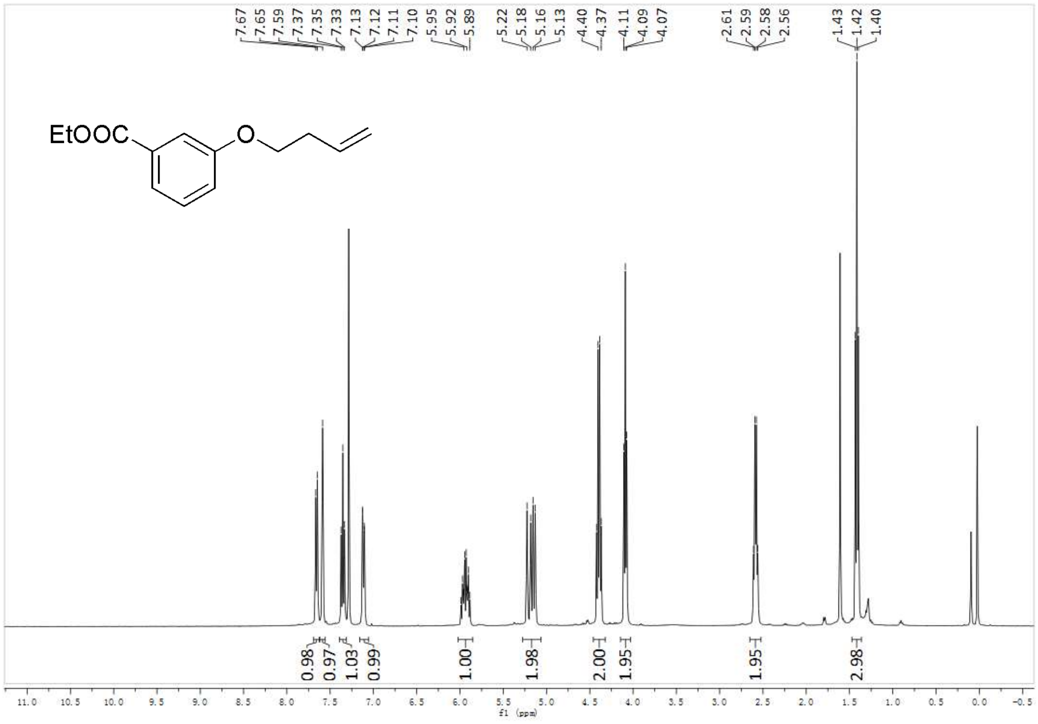
7.67
7.65
7.59
7.37
7.35
7.33
7.13
7.12
7.11
7.10
5.95
5.92
5.89
5.22
5.18
5.16
5.13
4.40
4.37
4.11
4.09
4.07
2.61
2.59
2.58
2.56
1.43
1.42
1.40
EtOOC
O
0.98
0.97
1.03
0.99
1.00
1.98
2.00
1.95
1.95
2.98
11.0 10.5 10.0 9.5 9.0 8.5 8.0 7.5 7.0 6.5 6.0 5.5 5.0 4.5 4.0 3.5 3.0 2.5 2.0 1.5 1.0 0.5 0.0 -0.5
f1 (ppm)

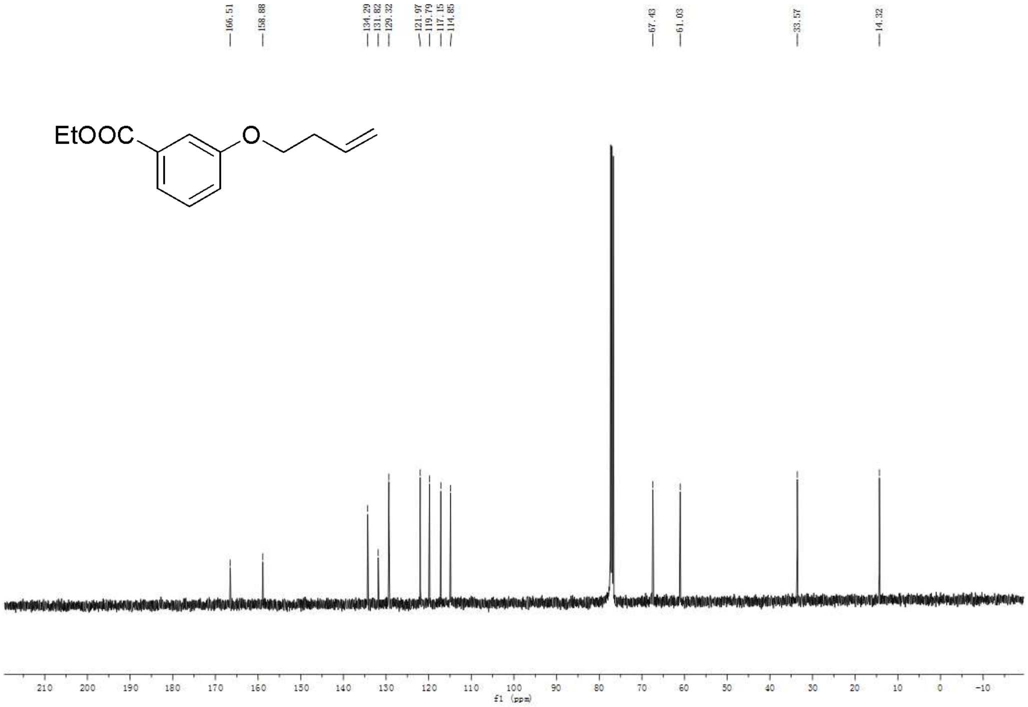
166.51
158.88
134.29
131.82
129.32
121.97
119.79
117.15
114.85
67.43
61.03
33.57
14.32
EtOOC
O
210 200 190 180 170 160 150 140 130 120 110 100 90 80 70 60 50 40 30 20 10 0 -10
f1 (ppm)

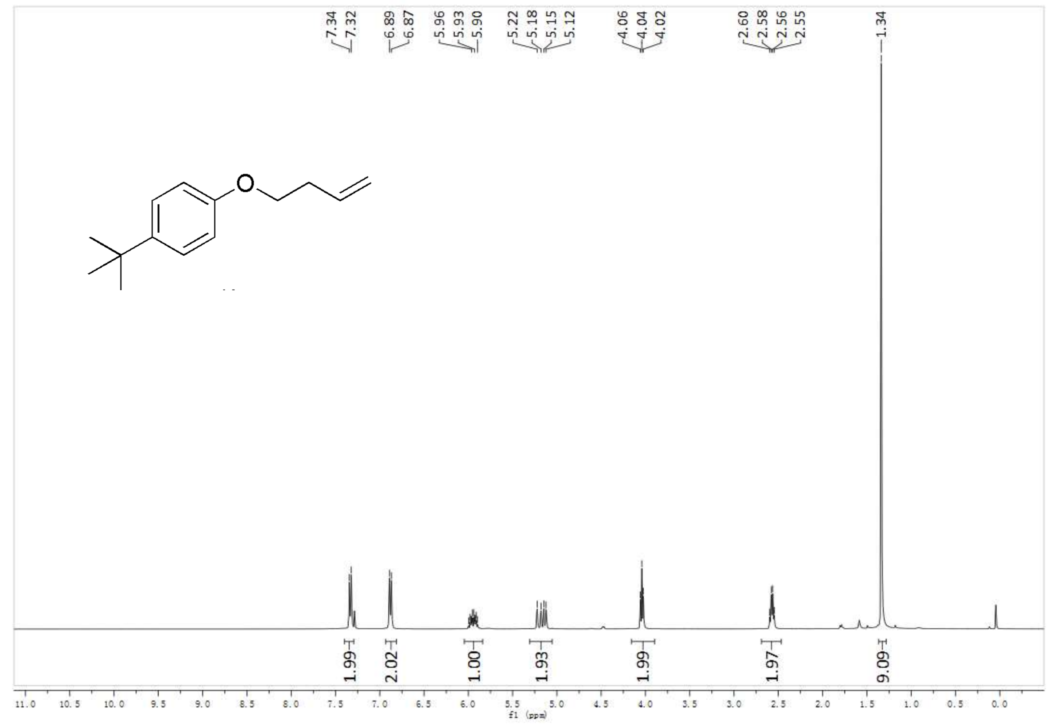
7.34
7.32
6.89
6.87
5.96
5.93
5.90
5.22
5.18
5.15
5.12
4.06
4.04
4.02
2.60
2.58
2.56
2.55
1.34
1.99
2.02
1.00
1.93
1.99
1.97
9.09
f1 (ppm)

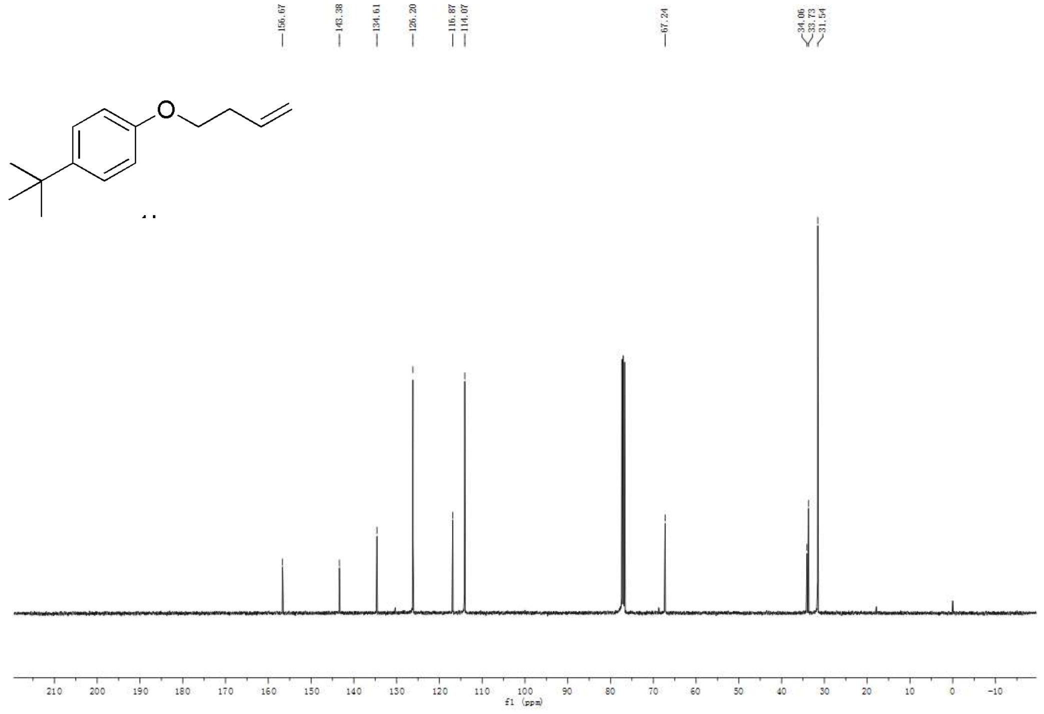
156.67
143.38
134.61
126.20
116.87
114.07
67.24
34.06
33.73
31.54
f1 (ppm)

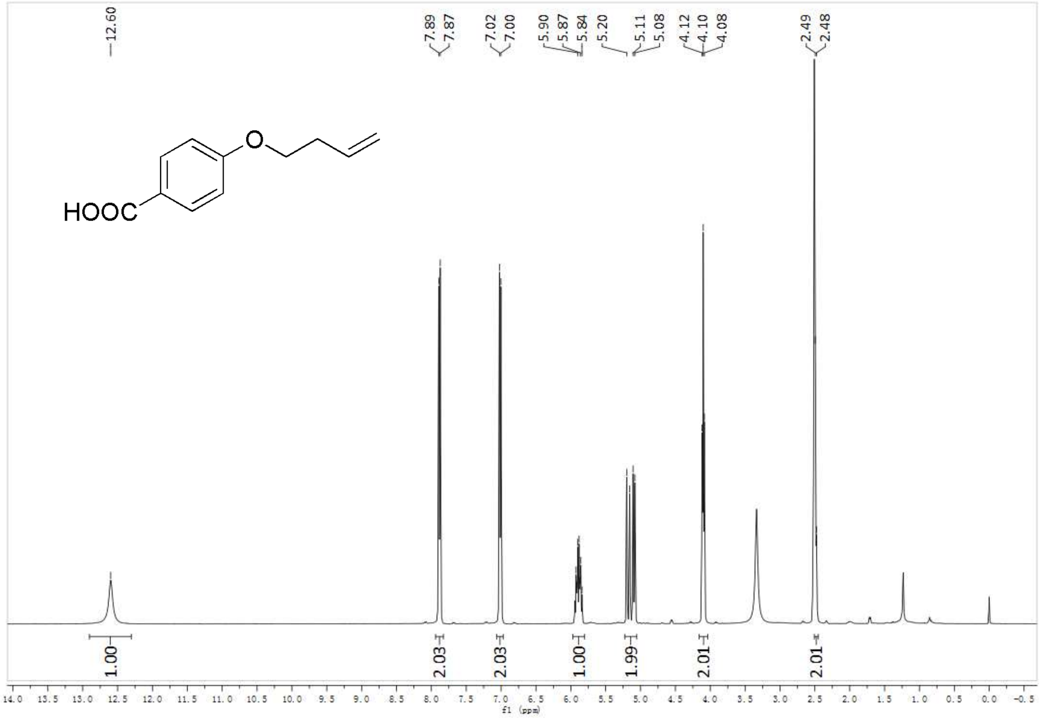

HOOC
O
12.60
7.89
7.87
7.02
7.00
5.90
5.87
5.84
5.20
5.11
5.08
4.12
4.10
4.08
2.49
2.48
1.00
2.03
2.03
1.00
1.99
2.01
2.01
f1 (ppm)

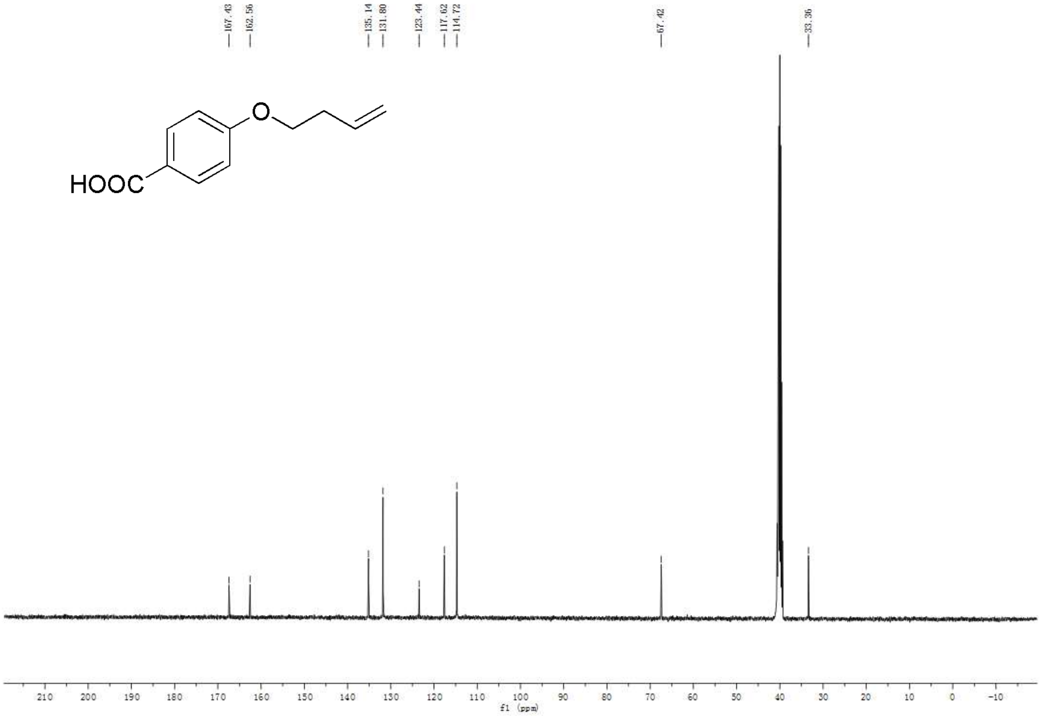

HOOC
O
167.43
162.56
135.14
131.80
123.44
117.62
114.72
67.42
33.36
f1 (ppm)

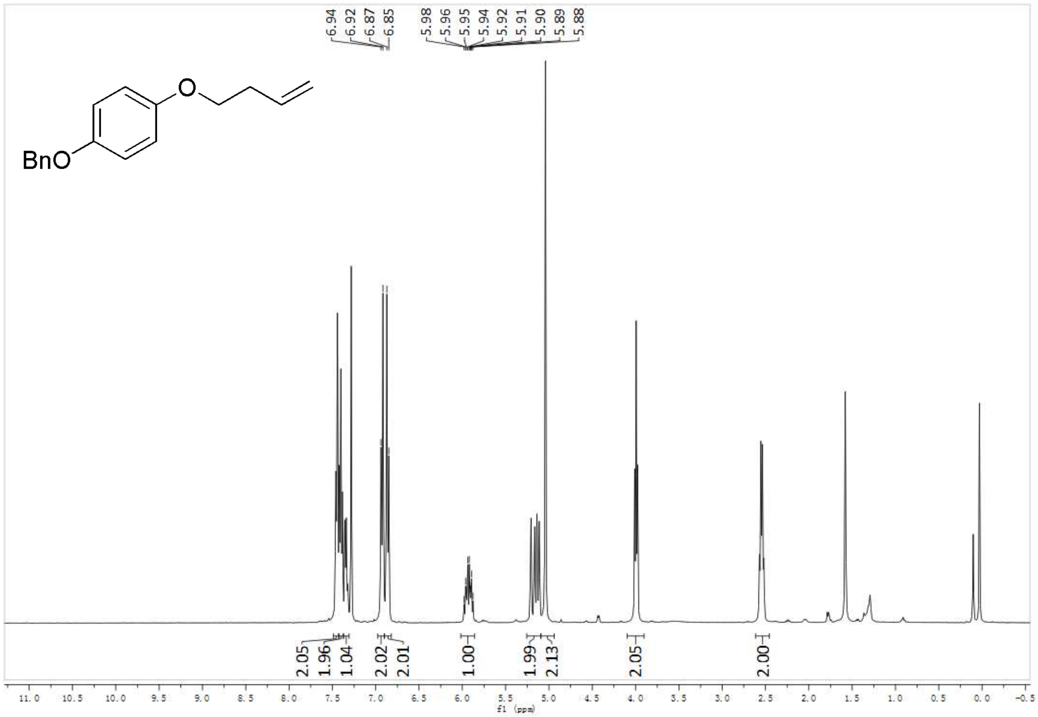
6.94
6.92
6.87
6.85
5.98
5.96
5.95
5.94
5.92
5.91
5.90
5.89
5.88
BnO
O
2.05
1.96
1.04
2.02
2.01
1.00
1.99
2.13
2.05
2.00
11.0 10.5 10.0 9.5 9.0 8.5 8.0 7.5 7.0 6.5 6.0 5.5 5.0 4.5 4.0 3.5 3.0 2.5 2.0 1.5 1.0 0.5 0.0 -0.5
f1 (ppm)

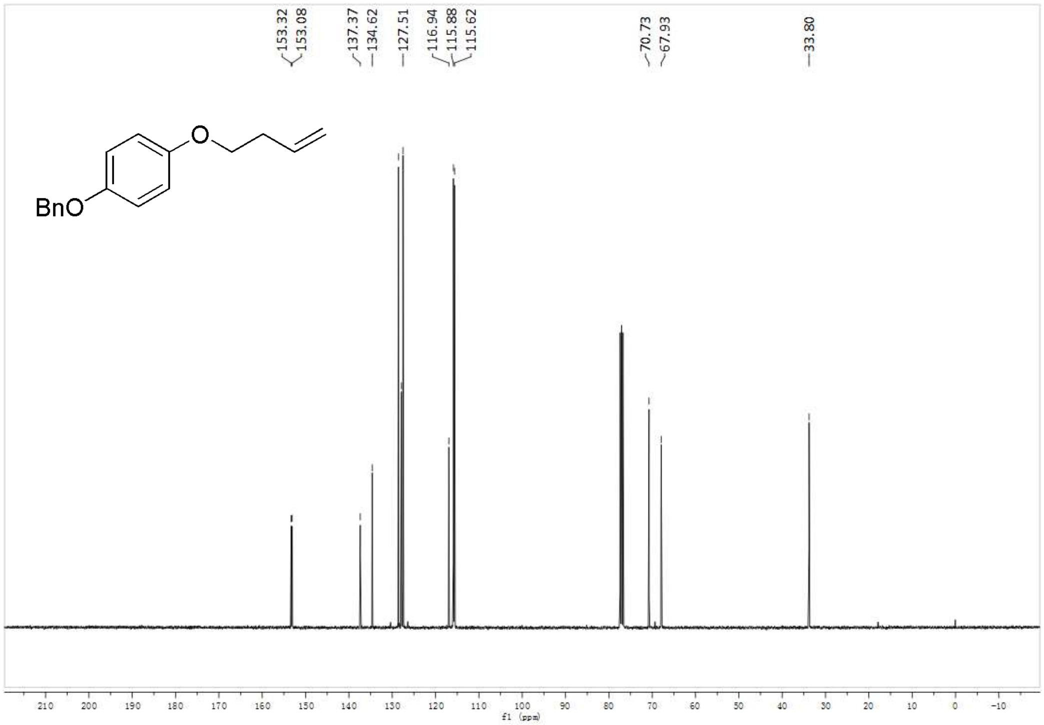
153.32
153.08
137.37
134.62
127.51
116.94
115.88
115.62
70.73
67.93
33.80
BnO
O
210 200 190 180 170 160 150 140 130 120 110 100 90 80 70 60 50 40 30 20 10 0 -10
f1 (ppm)

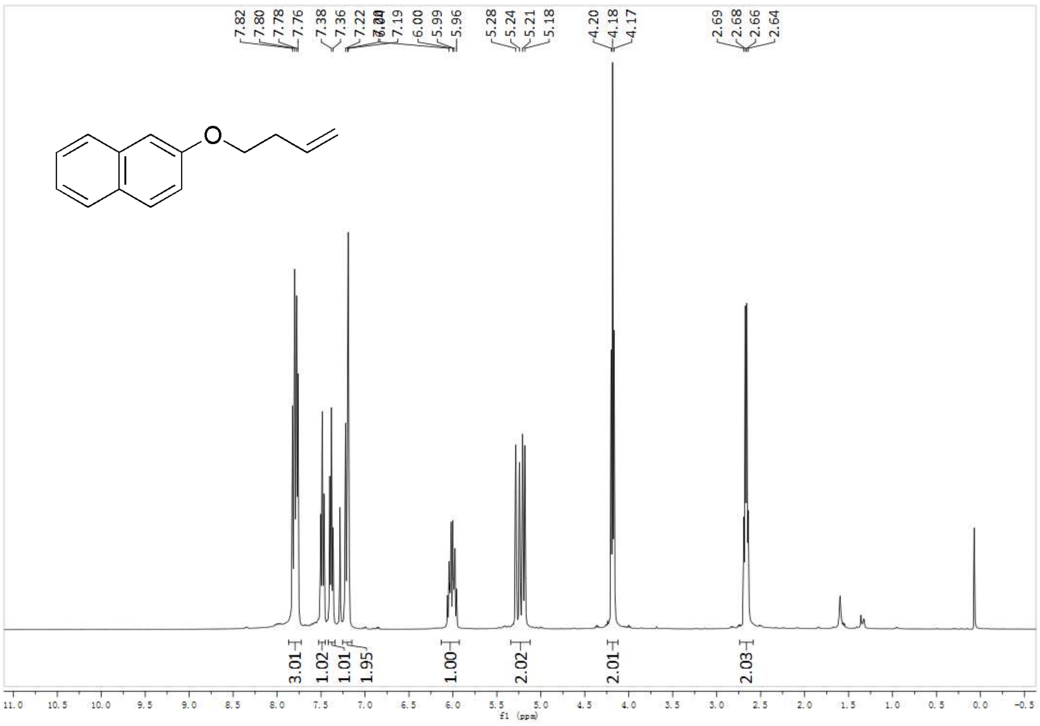
7.82
7.80
7.78
7.76
7.38
7.36
7.22
7.19
6.00
5.99
5.96
5.28
5.24
5.21
5.18
4.20
4.18
4.17
2.69
2.68
2.66
2.64
O
3.01
1.02
1.01
1.95
1.00
2.02
2.01
2.03
f1 (ppm)

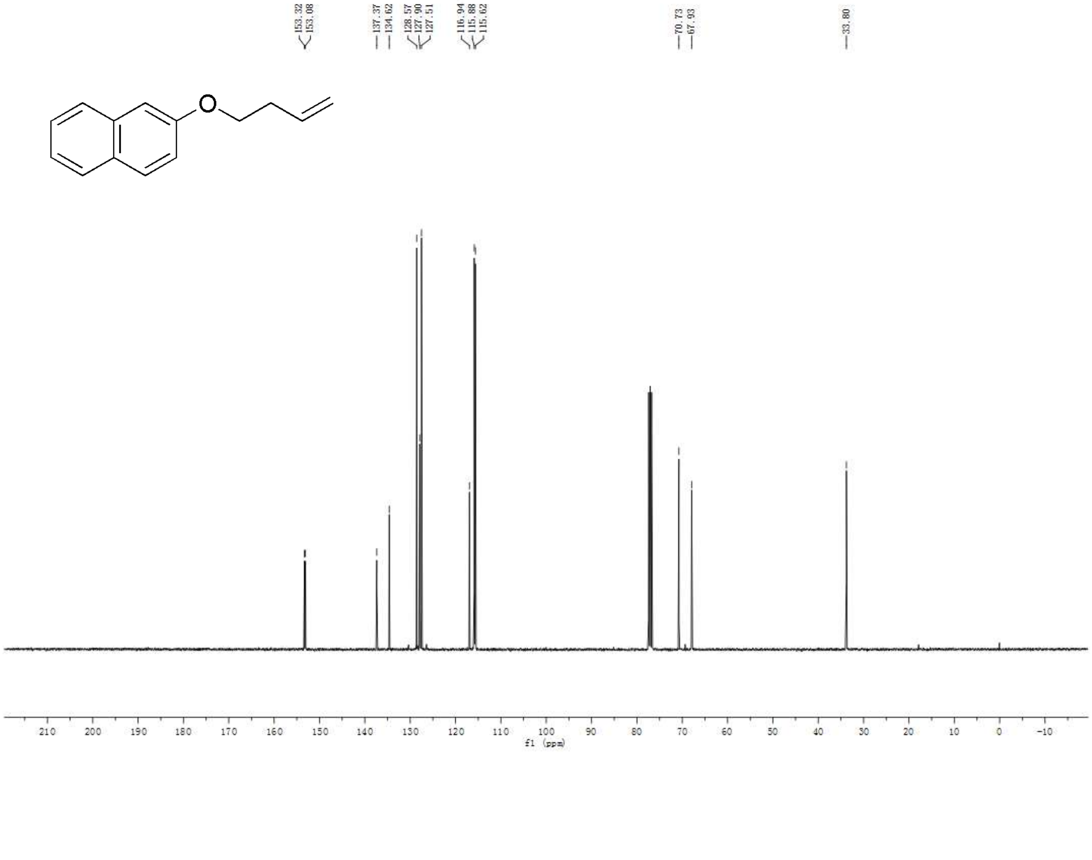
153.32
153.08
137.37
134.62
128.57
127.90
127.51
116.94
115.88
115.62
70.73
67.93
33.80
O
f1 (ppm)

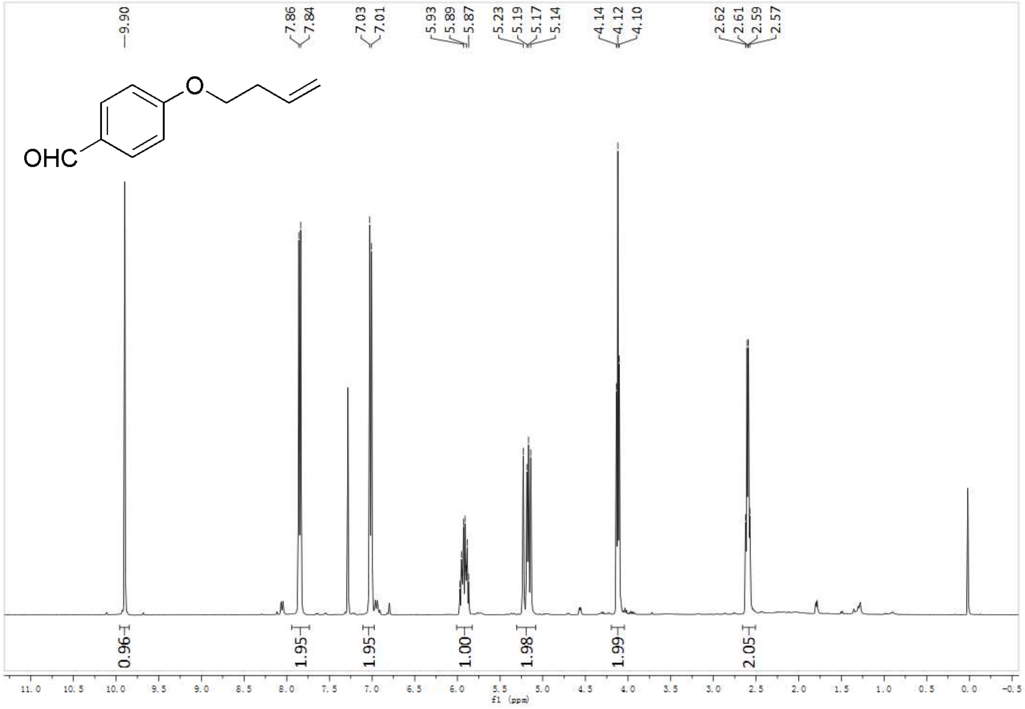

9.90
7.86
7.84
7.03
7.01
5.93
5.89
5.87
5.23
5.19
5.17
5.14
4.14
4.12
4.10
2.62
2.61
2.59
2.57
OHC
O
0.96
1.95
1.95
1.00
1.98
1.99
2.05
f1 (ppm)

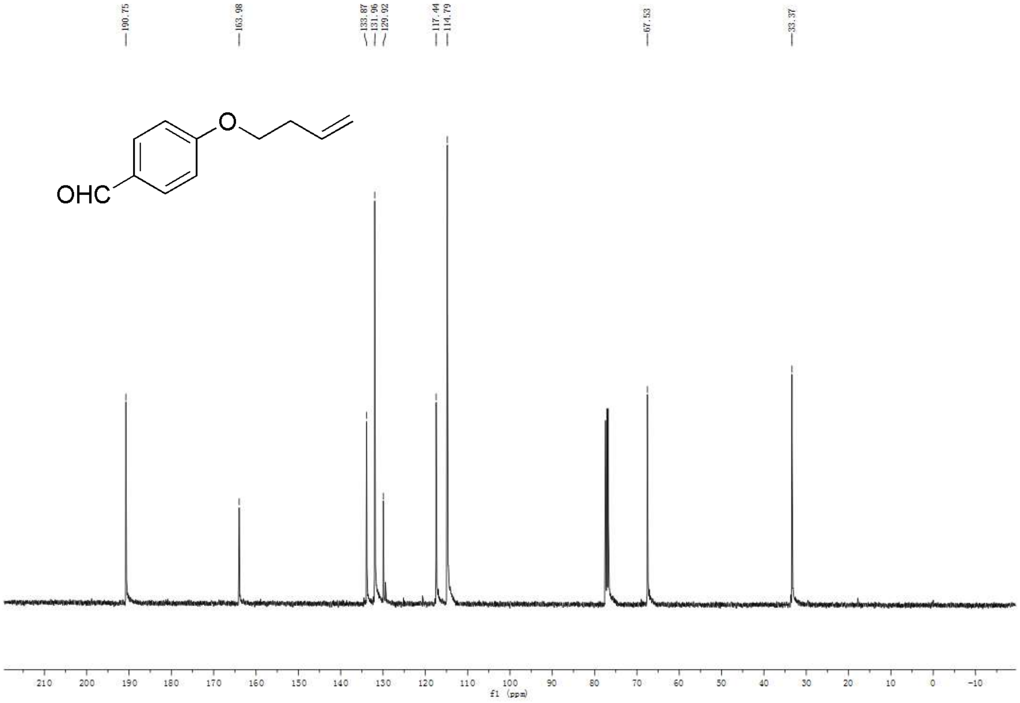

190.75
163.98
133.87
131.96
129.92
117.44
114.79
67.53
33.37
OHC
O
f1 (ppm)

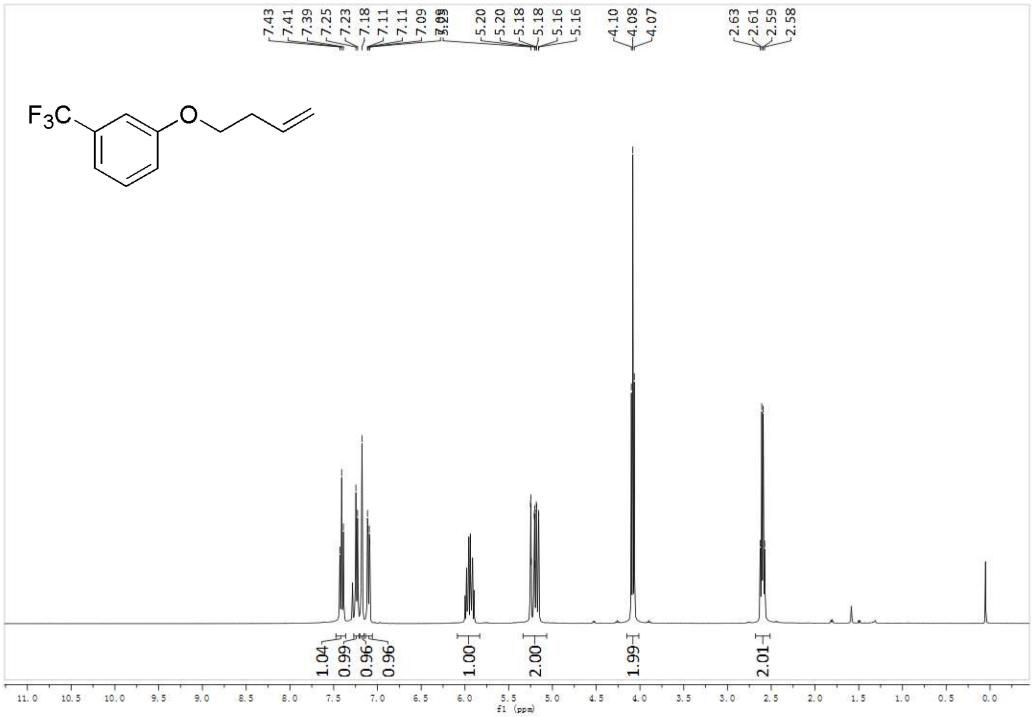

7.43
7.41
7.39
7.25
7.23
7.18
7.11
7.11
7.09
5.20
5.20
5.18
5.18
5.16
5.16
4.10
4.08
4.07
2.63
2.61
2.59
2.58
F3C
O
1.04
0.99
0.96
0.96
1.00
2.00
1.99
2.01
11.0
10.5
10.0
9.5
9.0
8.5
8.0
7.5
7.0
6.5
6.0
5.5
5.0
4.5
4.0
3.5
3.0
2.5
2.0
1.5
1.0
0.5
0.0
f1 (ppm)

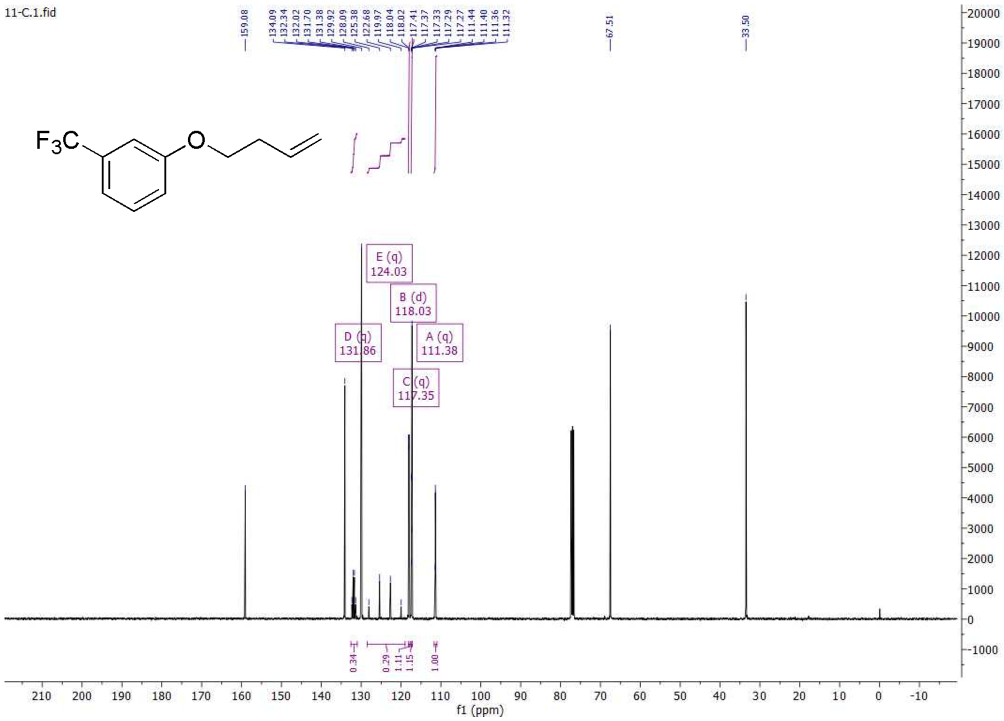

11-C.1.fid
159.08
134.09
132.34
132.02
131.70
131.38
129.92
128.09
125.38
122.68
119.97
118.04
118.02
117.41
117.37
117.33
117.29
117.27
111.44
111.40
111.36
111.32
67.51
33.50
F3C
O
E (q)
124.03
B (d)
118.03
D (q)
131.86
A (q)
111.38
C (q)
117.35
0.34
0.29
1.11
1.15
1.00
210
200
190
180
170
160
150
140
130
120
110
100
90
80
70
60
50
40
30
20
10
0
-10
f1 (ppm)

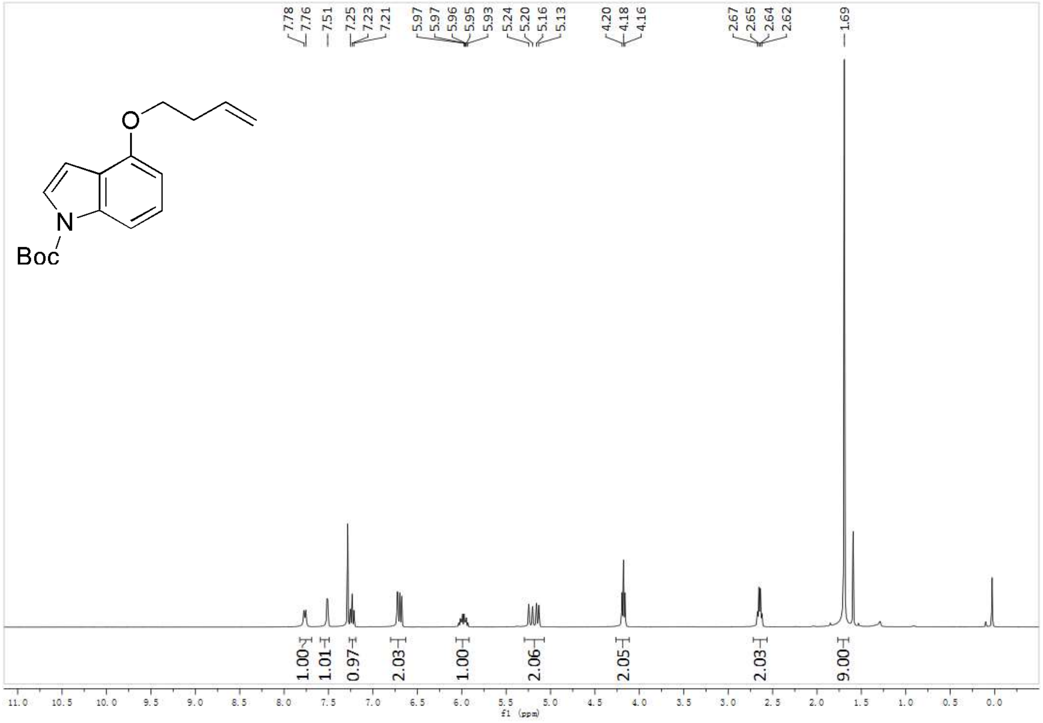
O
N
Boc
7.78
7.76
7.51
7.25
7.23
7.21
5.97
5.97
5.96
5.95
5.93
5.24
5.20
5.16
5.13
4.20
4.18
4.16
2.67
2.65
2.64
2.62
1.69
1.00
1.01
0.97
2.03
1.00
2.06
2.05
2.03
9.00
f1 (ppm)

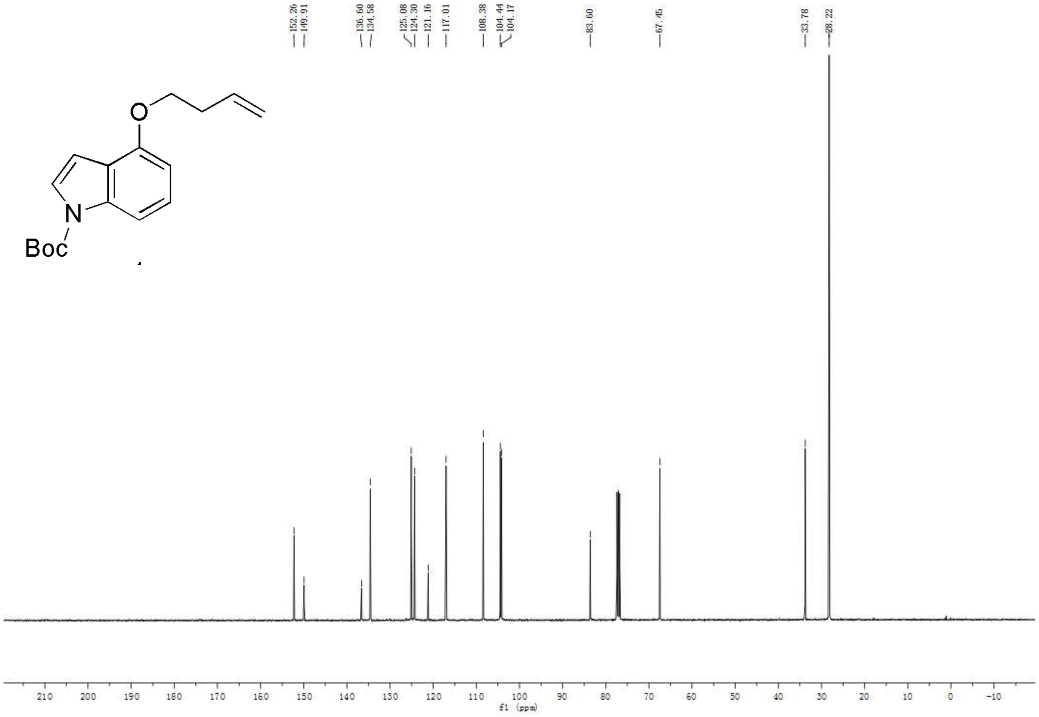
O
N
Boc
152.26
149.91
136.60
134.58
125.08
124.30
121.16
117.01
108.38
104.44
104.17
83.60
67.45
33.78
28.22
f1 (ppm)

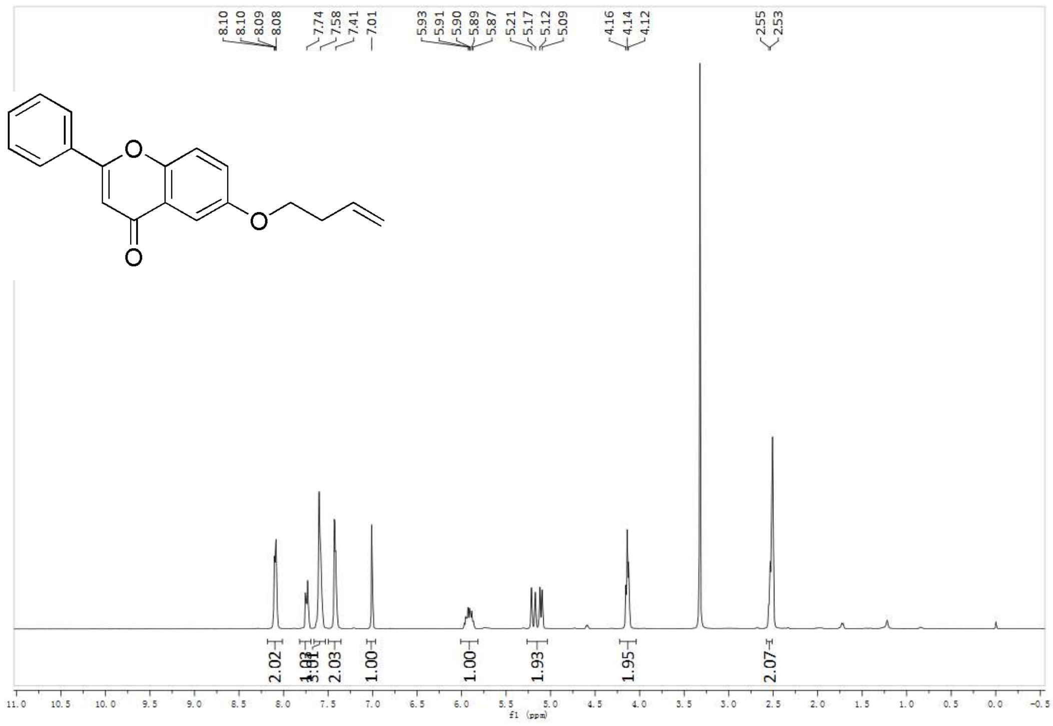
8.10
8.10
8.09
8.08
7.74
7.58
7.41
7.01
5.93
5.91
5.90
5.89
5.87
5.21
5.17
5.12
5.09
4.16
4.14
4.12
2.55
2.53
2.02
2.03
1.00
1.00
1.93
1.95
2.07
f1 (ppm)

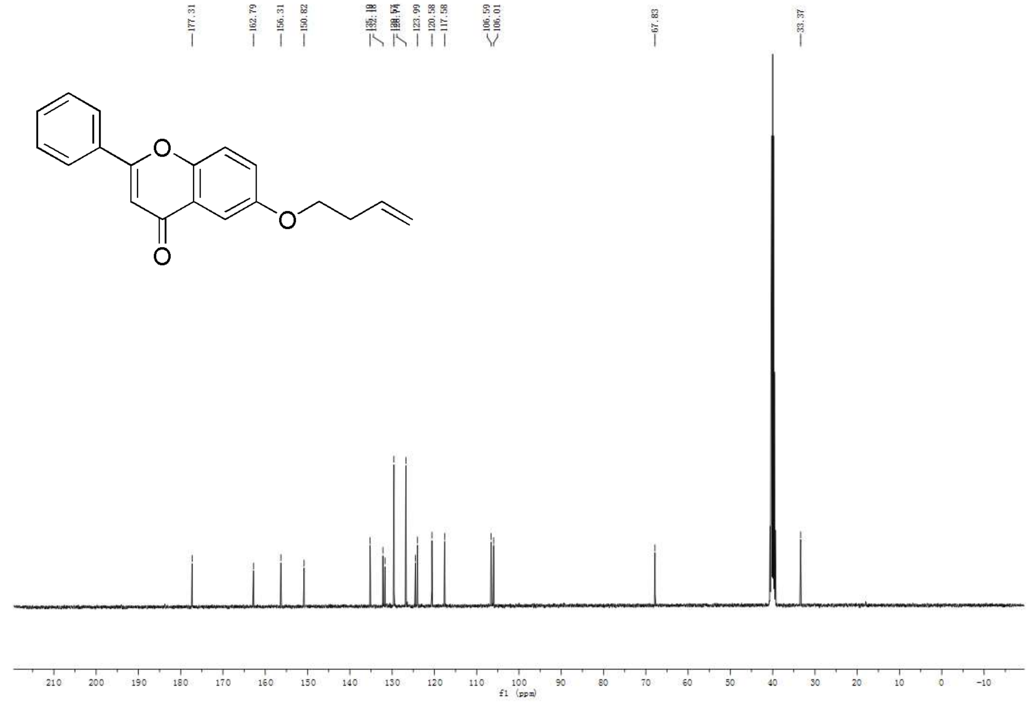
177.31
162.79
156.31
150.82
123.99
120.58
117.58
106.59
106.01
67.83
33.37
f1 (ppm)

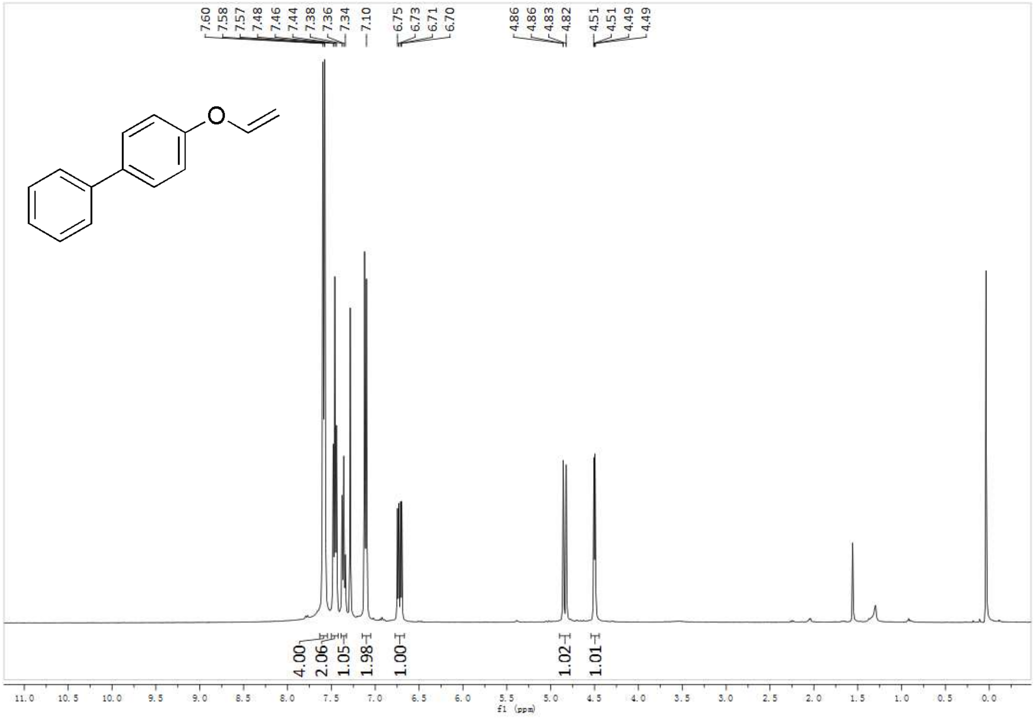

7.60
7.58
7.57
7.48
7.46
7.44
7.38
7.36
7.34
7.10
6.75
6.73
6.71
6.70
4.86
4.86
4.83
4.82
4.51
4.51
4.49
4.49
4.00
2.06
1.05
1.98
1.00
1.02
1.01
11.0 10.5 10.0 9.5 9.0 8.5 8.0 7.5 7.0 6.5 6.0 5.5 5.0 4.5 4.0 3.5 3.0 2.5 2.0 1.5 1.0 0.5 0.0
f1 (ppm)

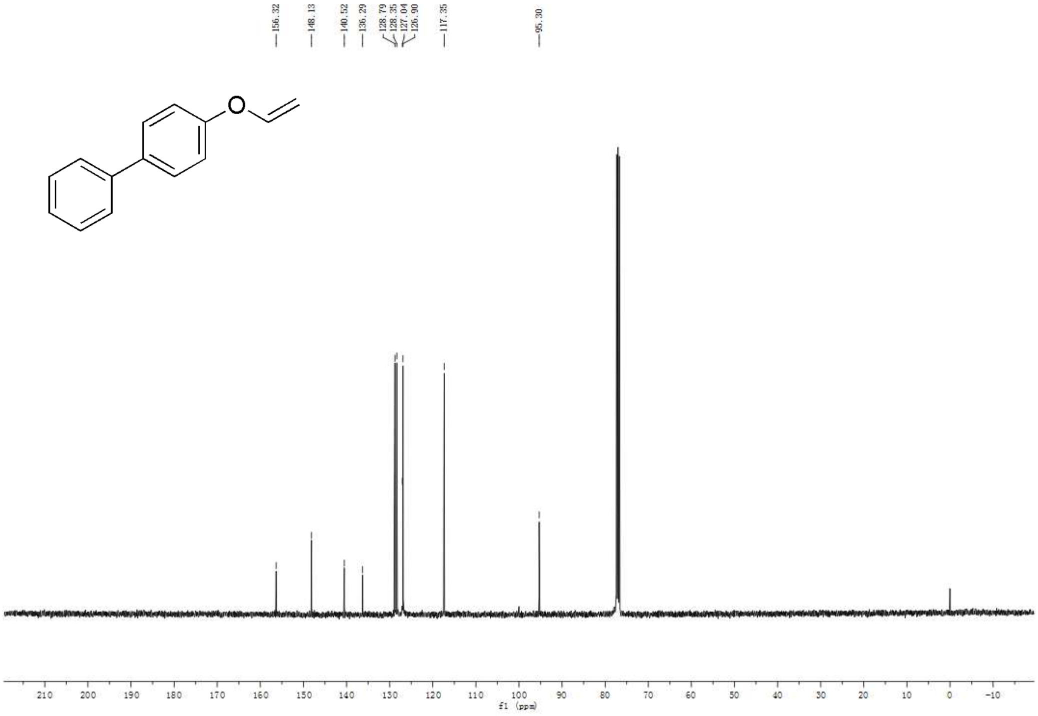

156.32
148.13
140.52
136.29
128.79
128.35
127.04
126.90
117.35
95.30
210 200 190 180 170 160 150 140 130 120 110 100 90 80 70 60 50 40 30 20 10 0 -10
f1 (ppm)

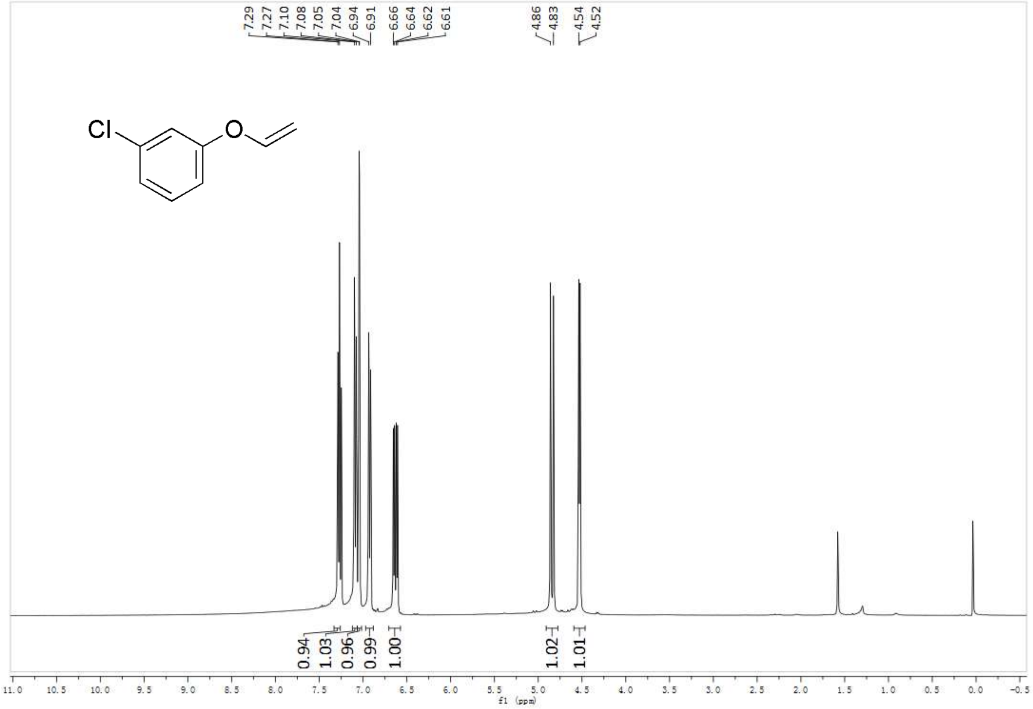

7.29
7.27
7.10
7.08
7.05
7.04
6.94
6.91
6.66
6.64
6.62
6.61
4.86
4.83
4.54
4.52
Cl
O
0.94
1.03
0.96
0.99
1.00
1.02
1.01
f1 (ppm)

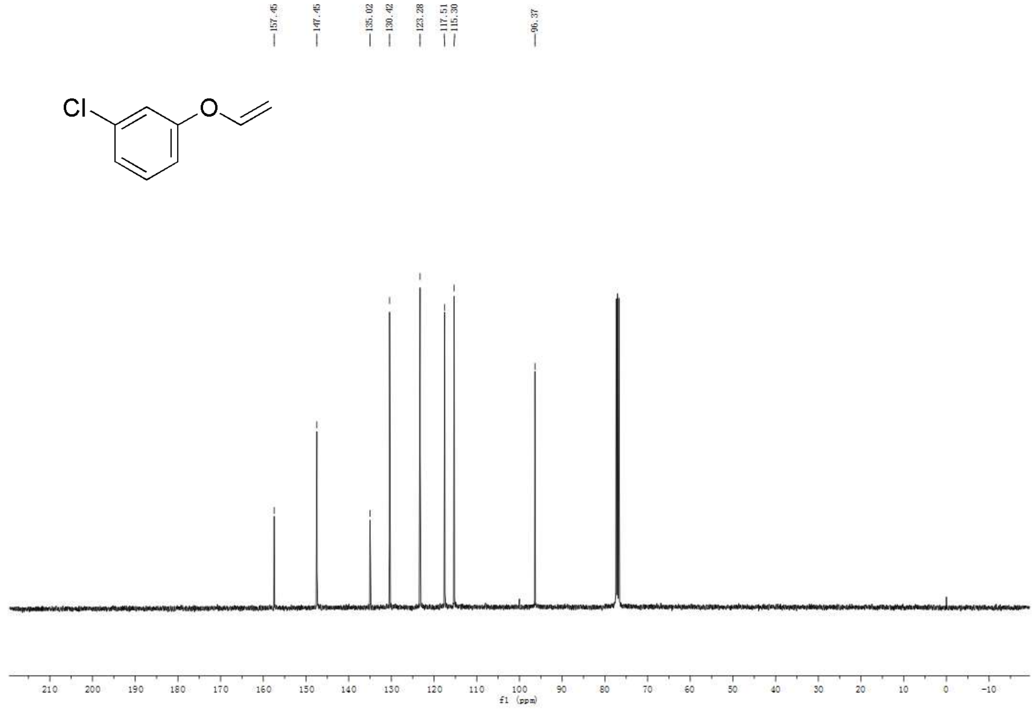

157.45
147.45
135.02
130.42
123.28
117.51
115.30
96.37
Cl
O
f1 (ppm)

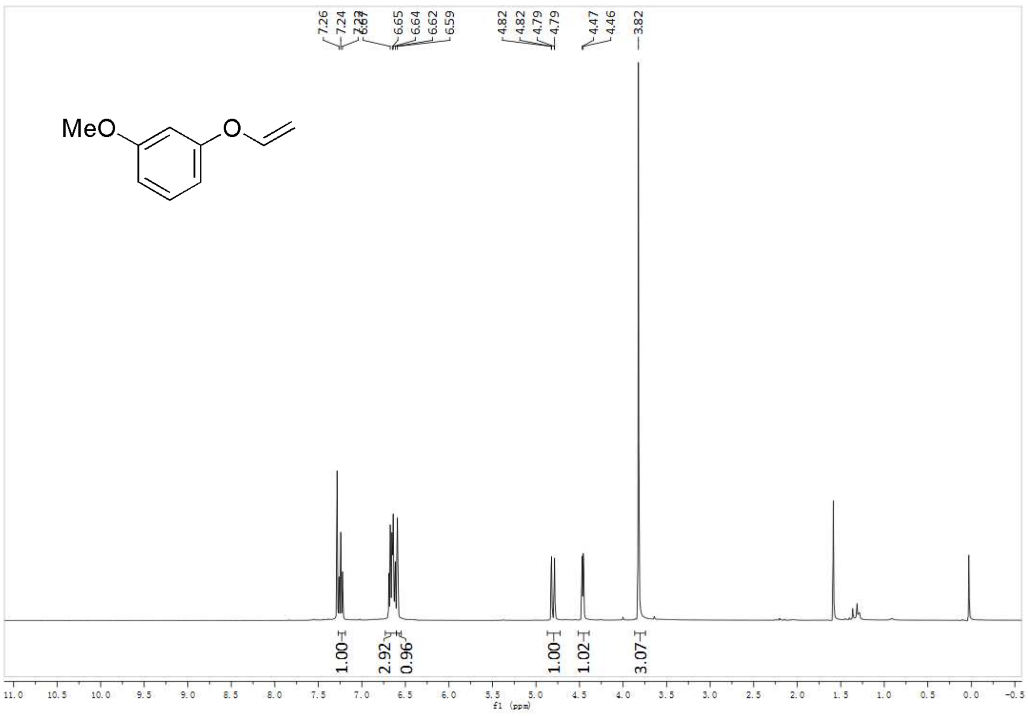

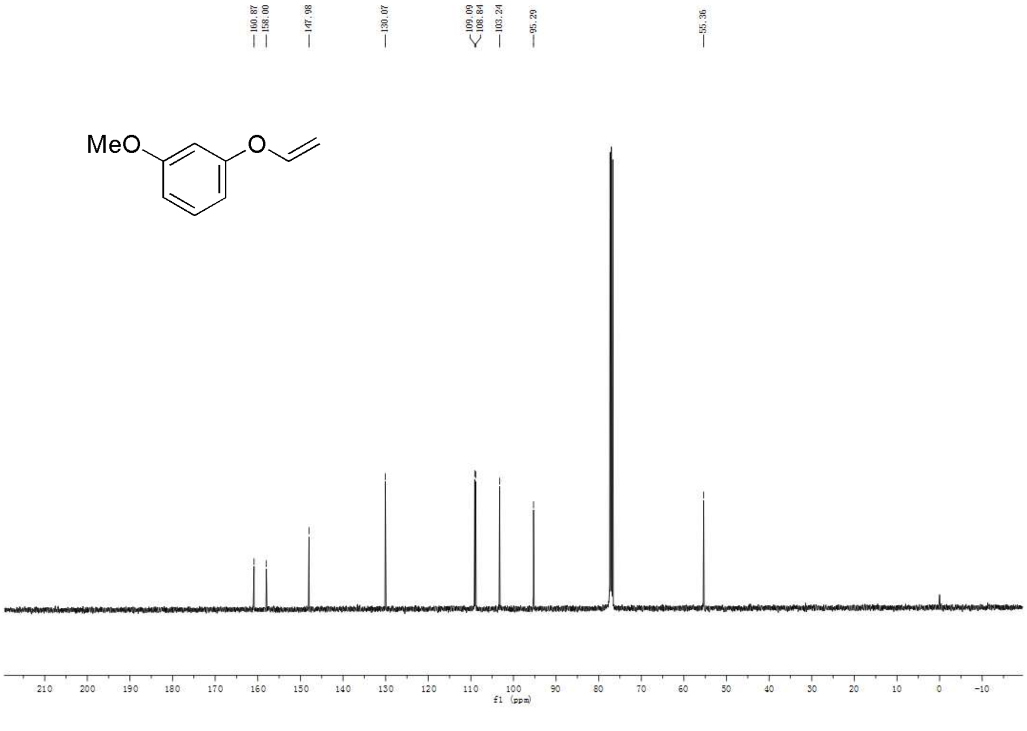

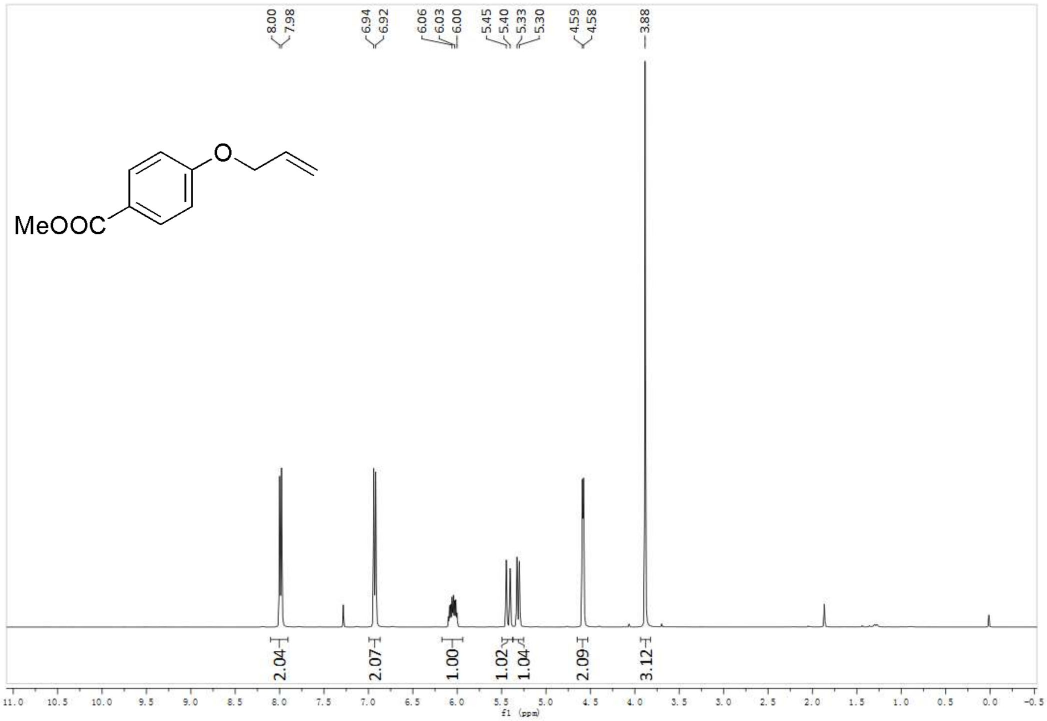

8.00
7.98
6.94
6.92
6.06
6.03
6.00
5.45
5.40
5.33
5.30
4.59
4.58
3.88
MeOOC
O
2.04
2.07
1.00
1.02
1.04
2.09
3.12
f1 (ppm)

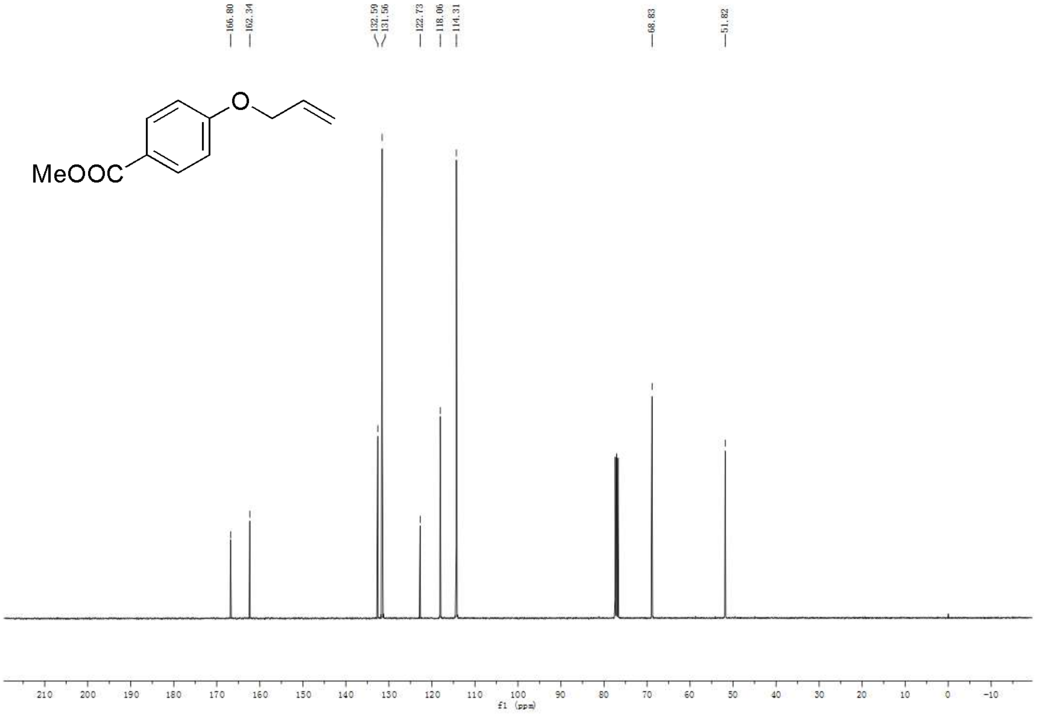

166.80
162.34
132.59
131.56
122.73
118.06
114.31
68.83
51.82
MeOOC
O
f1 (ppm)

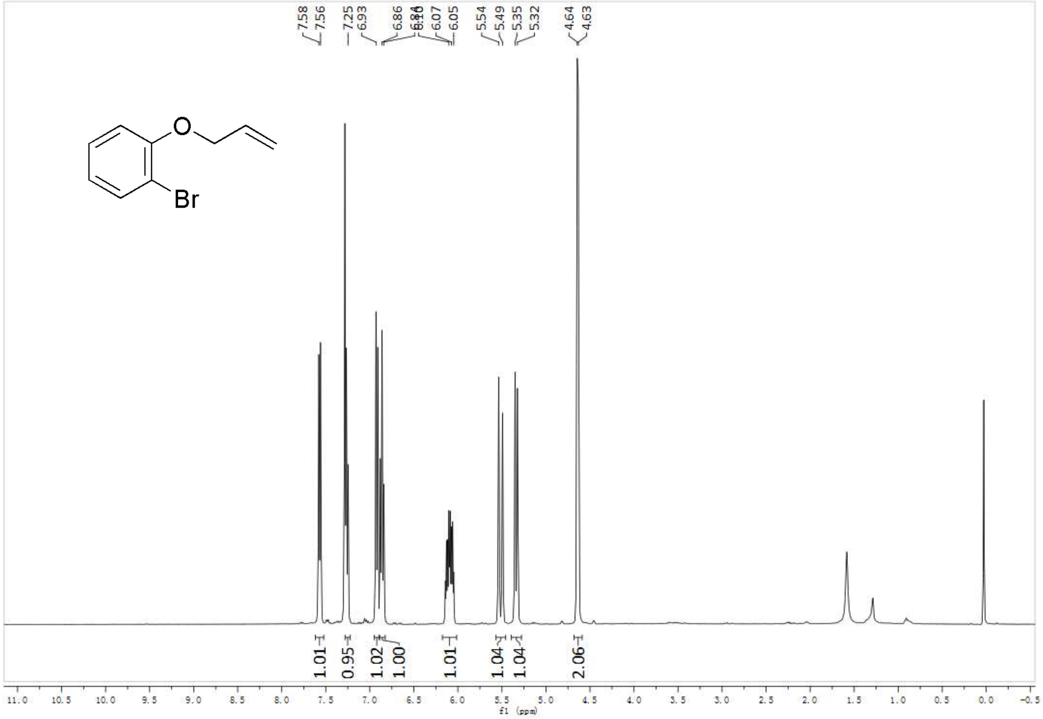
7.58
7.56
7.25
6.93
6.86
6.84
6.10
6.07
6.05
5.54
5.49
5.35
5.32
4.64
4.63
O
Br
1.01
0.95
1.02
1.00
1.01
1.04
1.04
2.06
f1 (ppm)

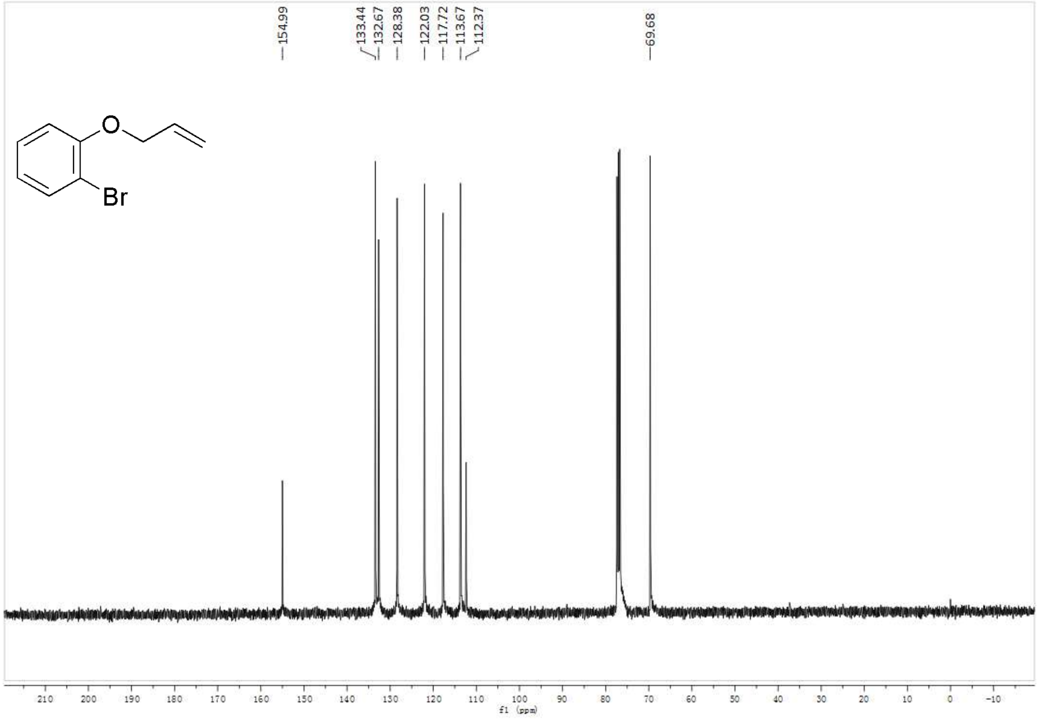
154.99
133.44
132.67
128.38
122.03
117.72
113.67
112.37
69.68
O
Br
f1 (ppm)

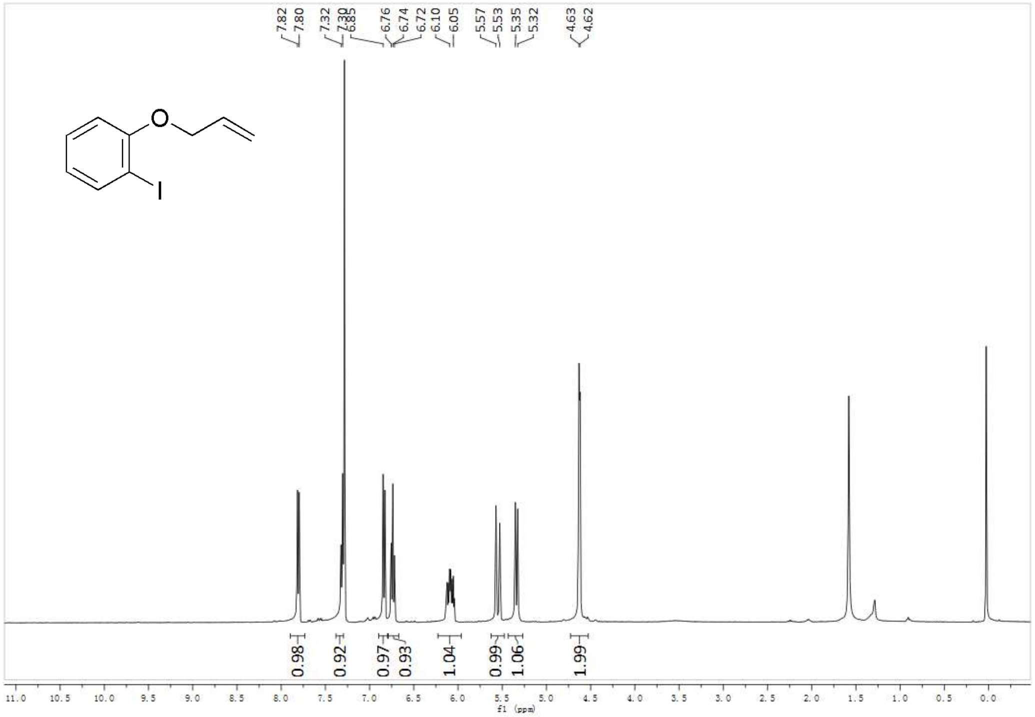

7.82
7.80
7.32
7.30
6.85
6.76
6.74
6.72
6.10
6.05
5.57
5.53
5.35
5.32
4.63
4.62
O
I
0.98
0.92
0.97
0.93
1.04
0.99
1.06
1.99
f1 (ppm)

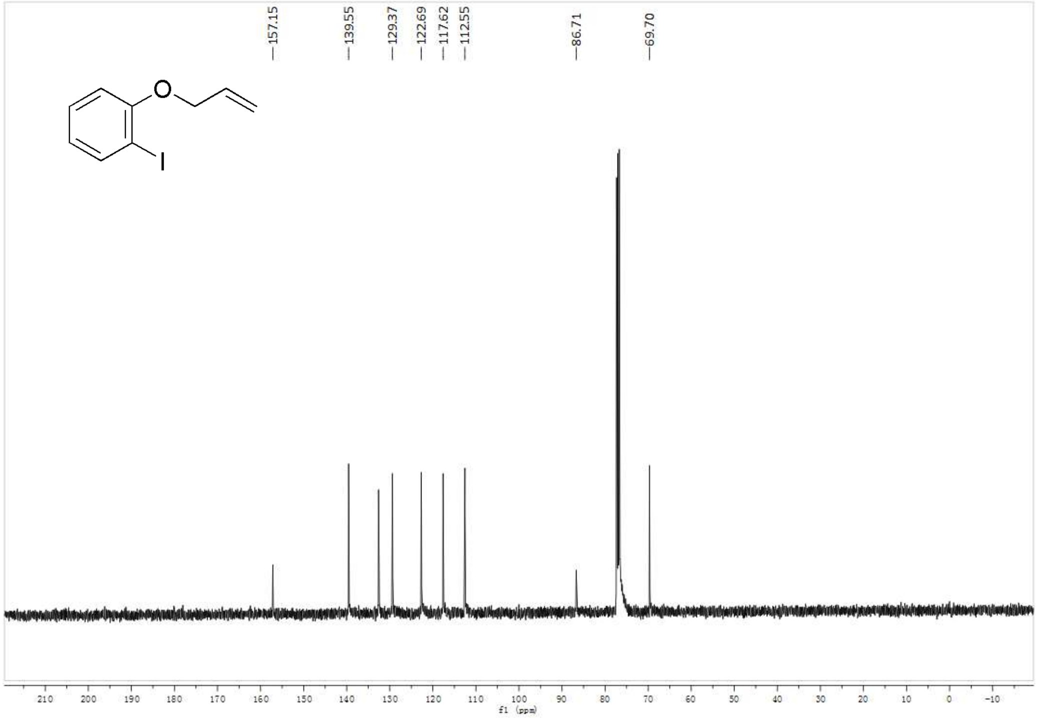

157.15
139.55
129.37
122.69
117.62
112.55
86.71
69.70
O
I
f1 (ppm)

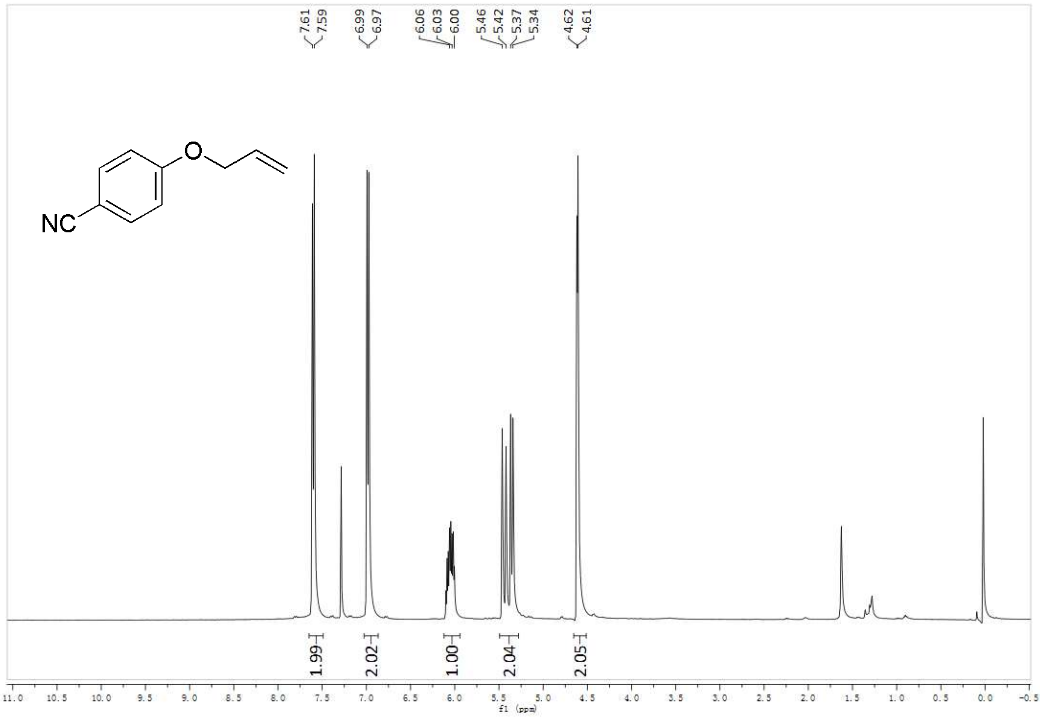
7.61
7.59
6.99
6.97
6.06
6.03
6.00
5.46
5.42
5.37
5.34
4.62
4.61
NC
O
1.99
2.02
1.00
2.04
2.05
11.0 10.5 10.0 9.5 9.0 8.5 8.0 7.5 7.0 6.5 6.0 5.5 5.0 4.5 4.0 3.5 3.0 2.5 2.0 1.5 1.0 0.5 0.0 -0.5
f1 (ppm)

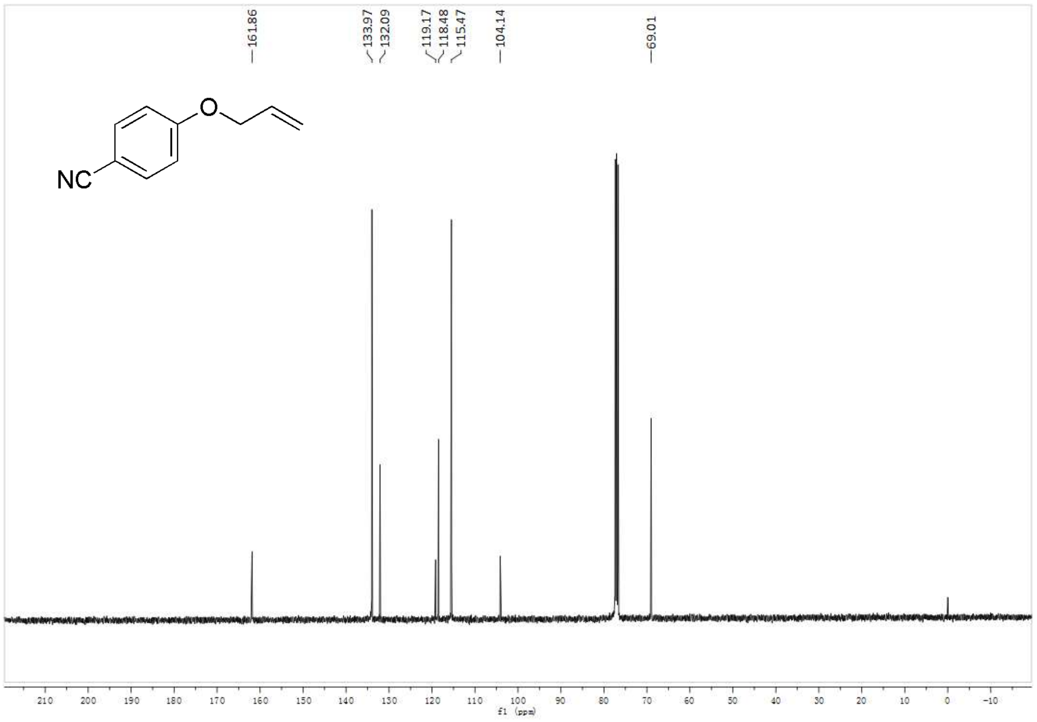
161.86
133.97
132.09
119.17
118.48
115.47
104.14
69.01
NC
O
210 200 190 180 170 160 150 140 130 120 110 100 90 80 70 60 50 40 30 20 10 0 -10
f1 (ppm)

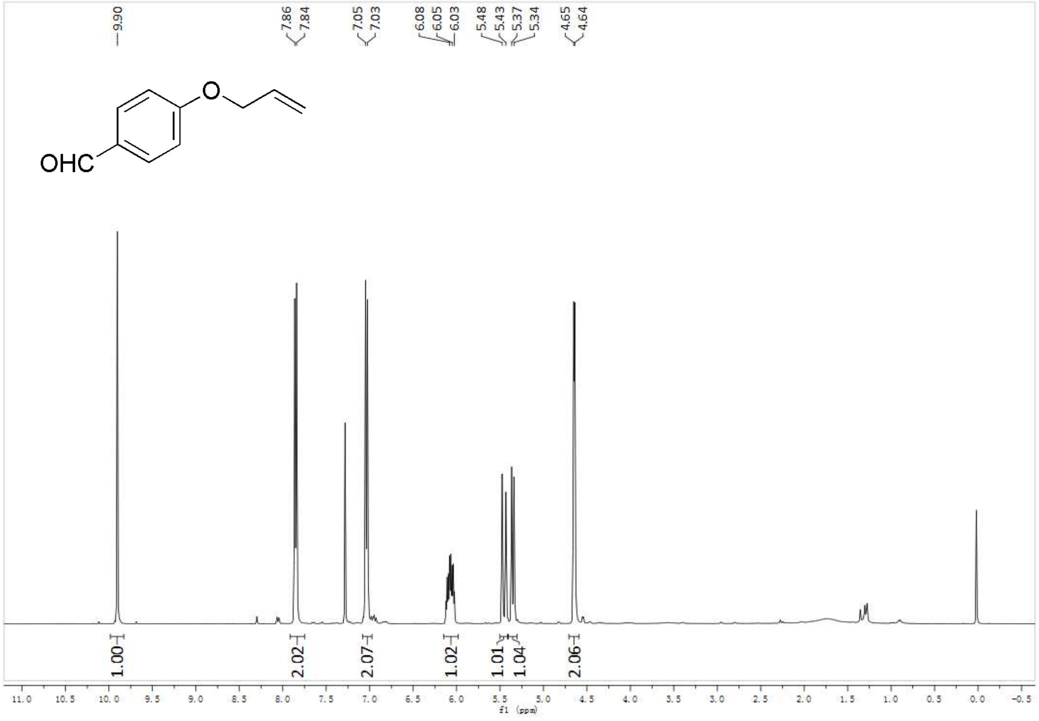

9.90
7.86
7.84
7.05
7.03
6.08
6.05
6.03
5.48
5.43
5.37
5.34
4.65
4.64
OHC
O
1.00
2.02
2.07
1.02
1.01
1.04
2.06
f1 (ppm)

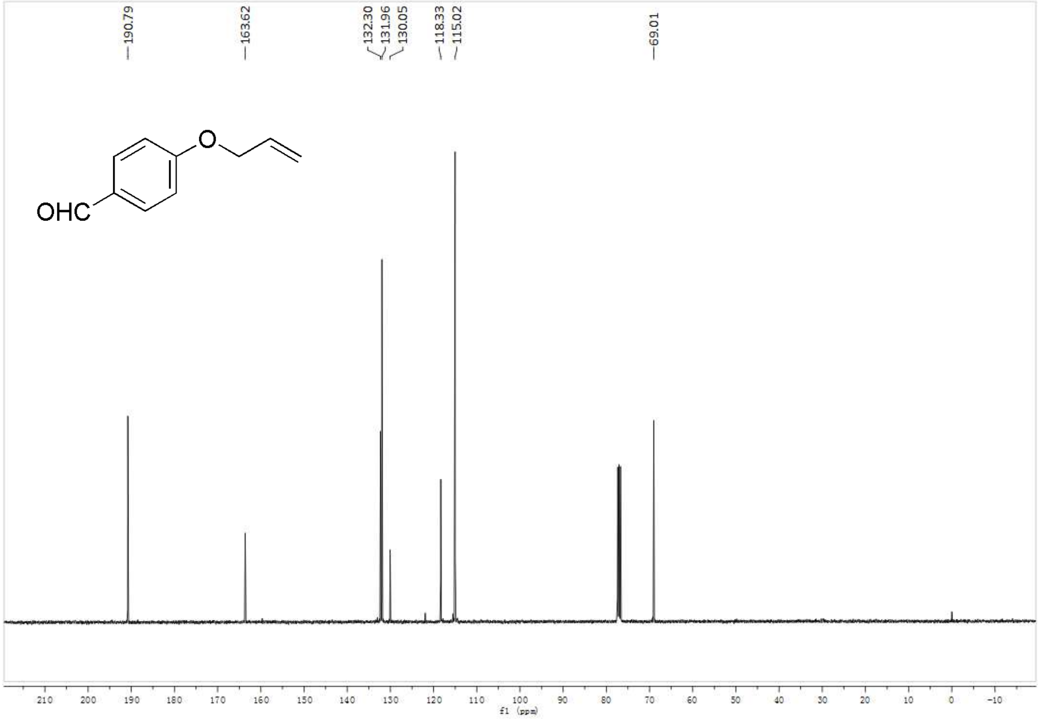

190.79
163.62
132.30
131.96
130.05
118.33
115.02
69.01
OHC
O
f1 (ppm)

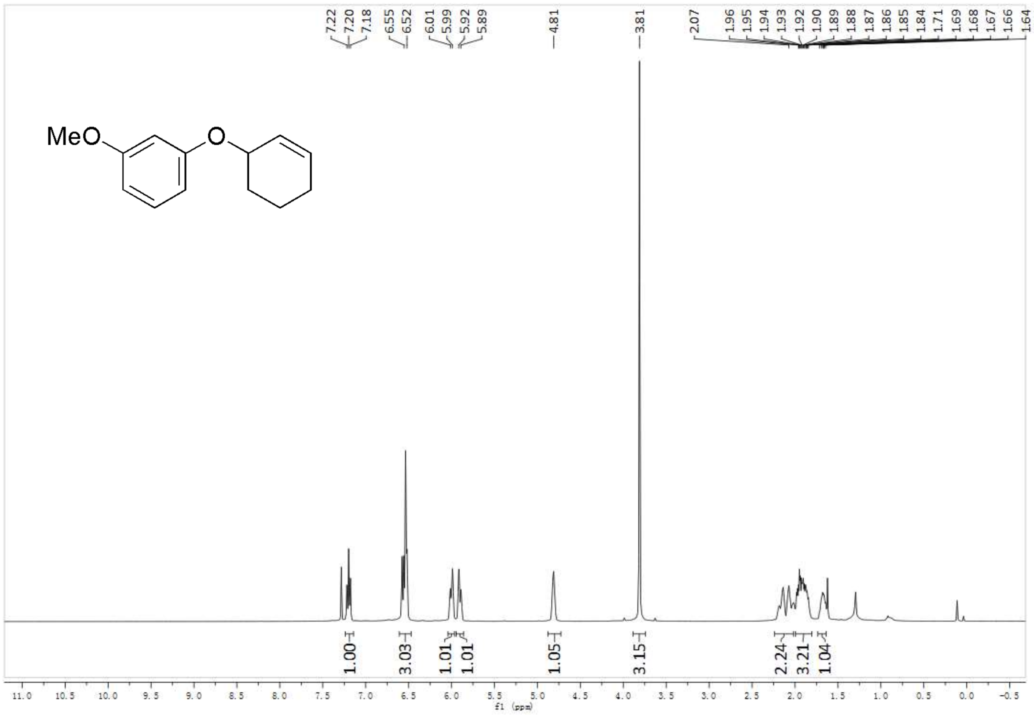

7.22
7.20
7.18
6.55
6.52
6.01
5.99
5.92
5.89
4.81
3.81
2.07
1.96
1.95
1.94
1.93
1.92
1.90
1.89
1.88
1.87
1.86
1.85
1.84
1.71
1.69
1.68
1.67
1.66
1.64
MeO
O
1.00
3.03
1.01
1.01
1.05
3.15
2.24
3.21
1.04
11.0 10.5 10.0 9.5 9.0 8.5 8.0 7.5 7.0 6.5 6.0 5.5 5.0 4.5 4.0 3.5 3.0 2.5 2.0 1.5 1.0 0.5 0.0 -0.5
f1 (ppm)

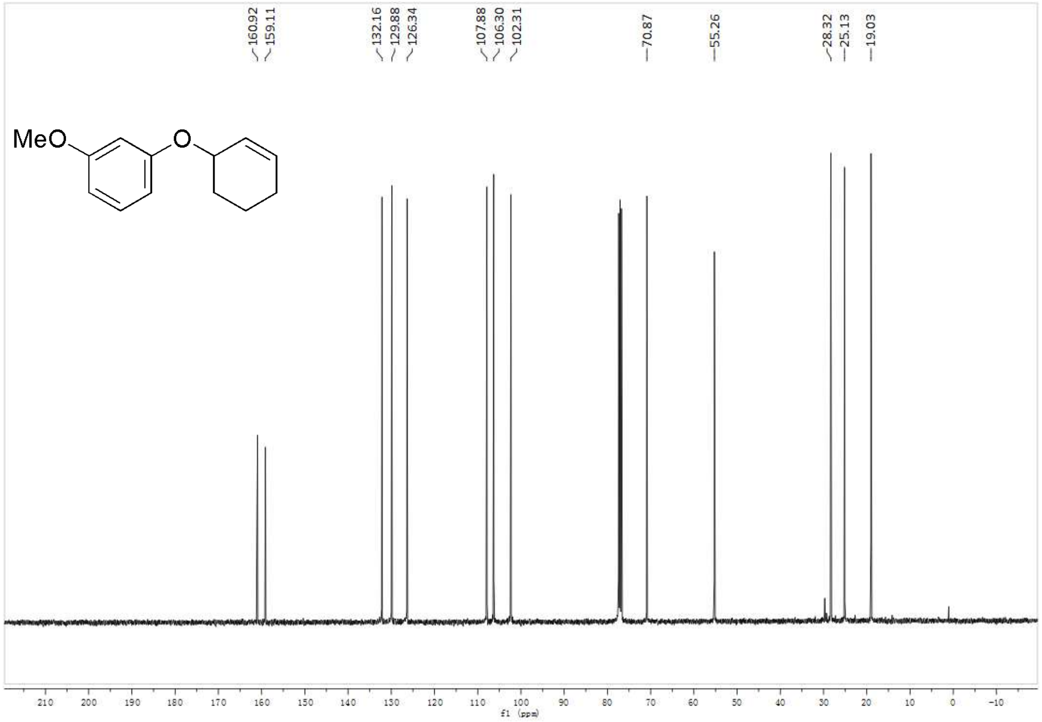

160.92
159.11
132.16
129.88
126.34
107.88
106.30
102.31
70.87
55.26
28.32
25.13
19.03
MeO
O
210 200 190 180 170 160 150 140 130 120 110 100 90 80 70 60 50 40 30 20 10 0 -10
f1 (ppm)

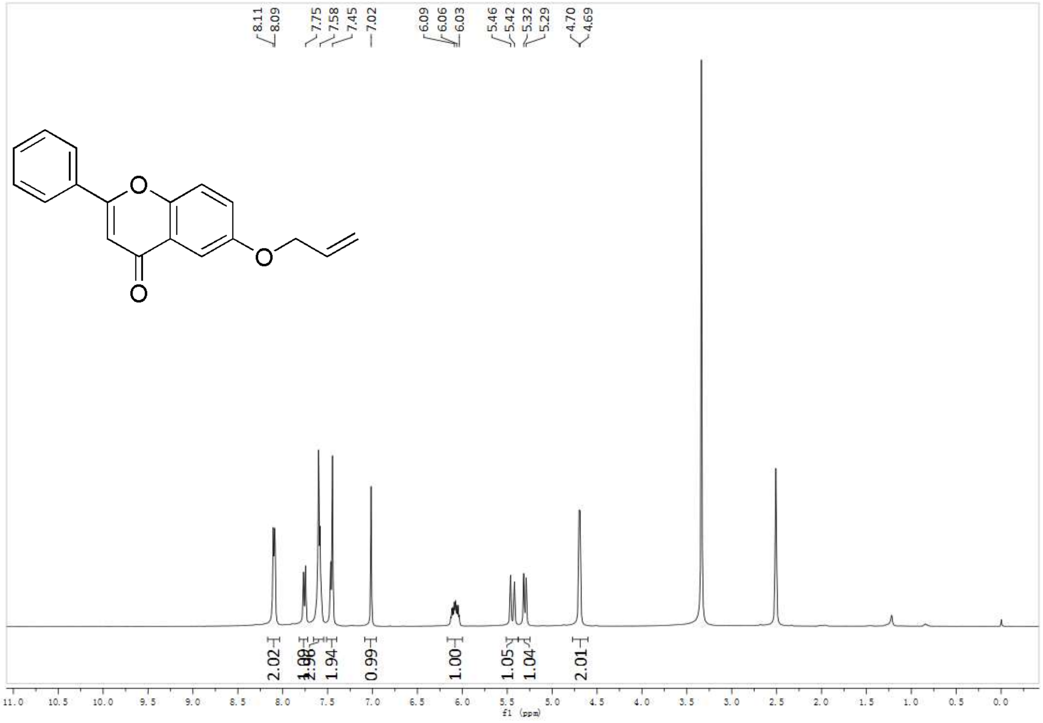

8.11
8.09
7.75
7.58
7.45
7.02
6.09
6.06
6.03
5.46
5.42
5.32
5.29
4.70
4.69
O
O
O
2.02
1.94
0.99
1.00
1.05
1.04
2.01
11.0 10.5 10.0 9.5 9.0 8.5 8.0 7.5 7.0 6.5 6.0 5.5 5.0 4.5 4.0 3.5 3.0 2.5 2.0 1.5 1.0 0.5 0.0
f1 (ppm)

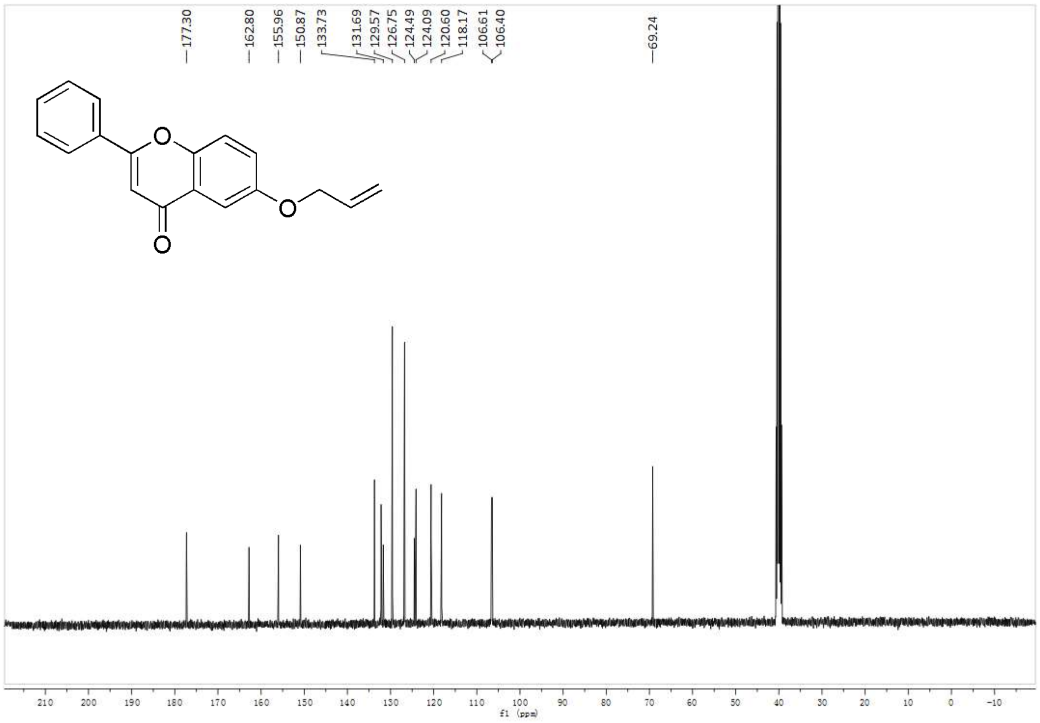

177.30
162.80
155.96
150.87
133.73
131.69
129.57
126.75
124.49
124.09
120.60
118.17
106.61
106.40
69.24
O
O
O
210 200 190 180 170 160 150 140 130 120 110 100 90 80 70 60 50 40 30 20 10 0 -10
f1 (ppm)

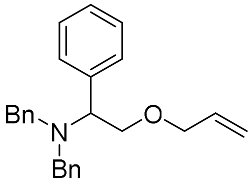

Bn
N
Bn
O

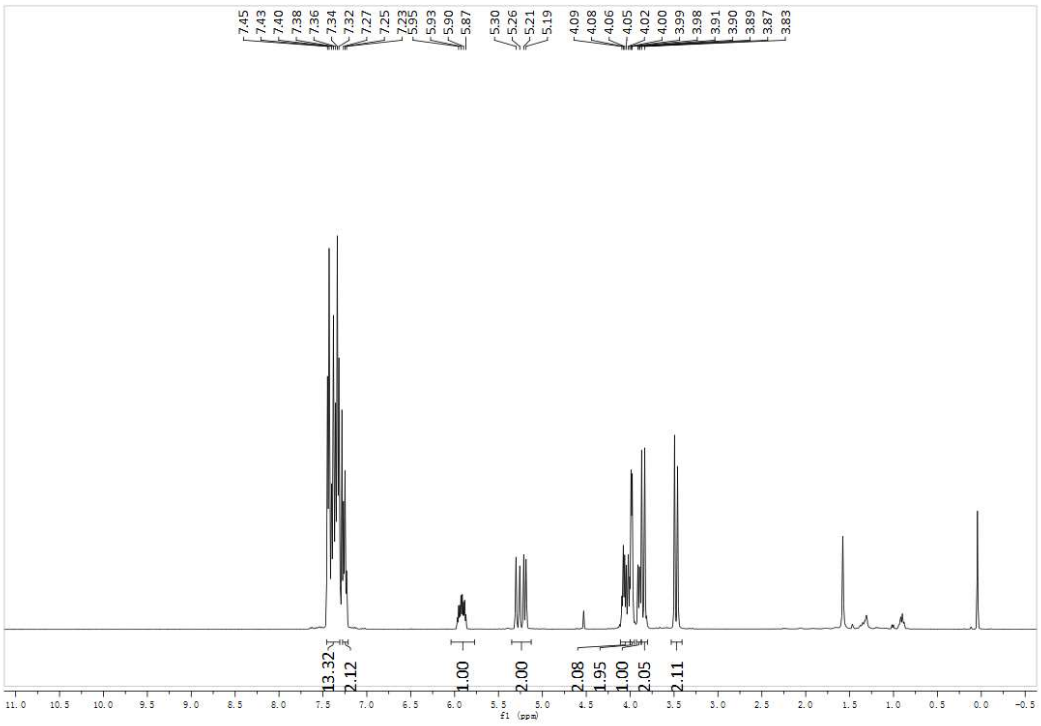
7.45 7.43 7.40 7.38 7.36 7.34 7.32 7.27 7.25 7.23 5.95 5.93 5.90 5.87 5.30 5.26 5.21 5.19 4.09 4.08 4.06 4.05 4.02 4.00 3.99 3.98 3.91 3.90 3.89 3.87 3.83
13.32 2.12 1.00 2.00 2.08 1.95 1.00 2.05 2.11
f1 (ppm)

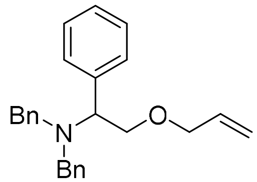
Bn
N
Bn
O

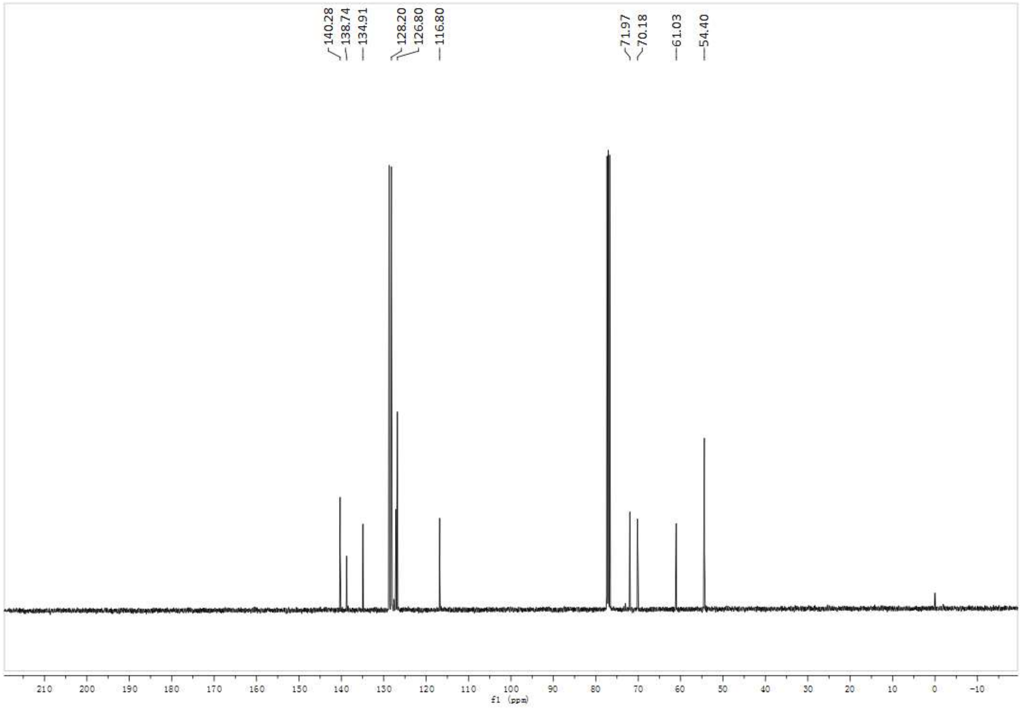
140.28 138.74 134.91 128.20 126.80 116.80 71.97 70.18 61.03 54.40
f1 (ppm)

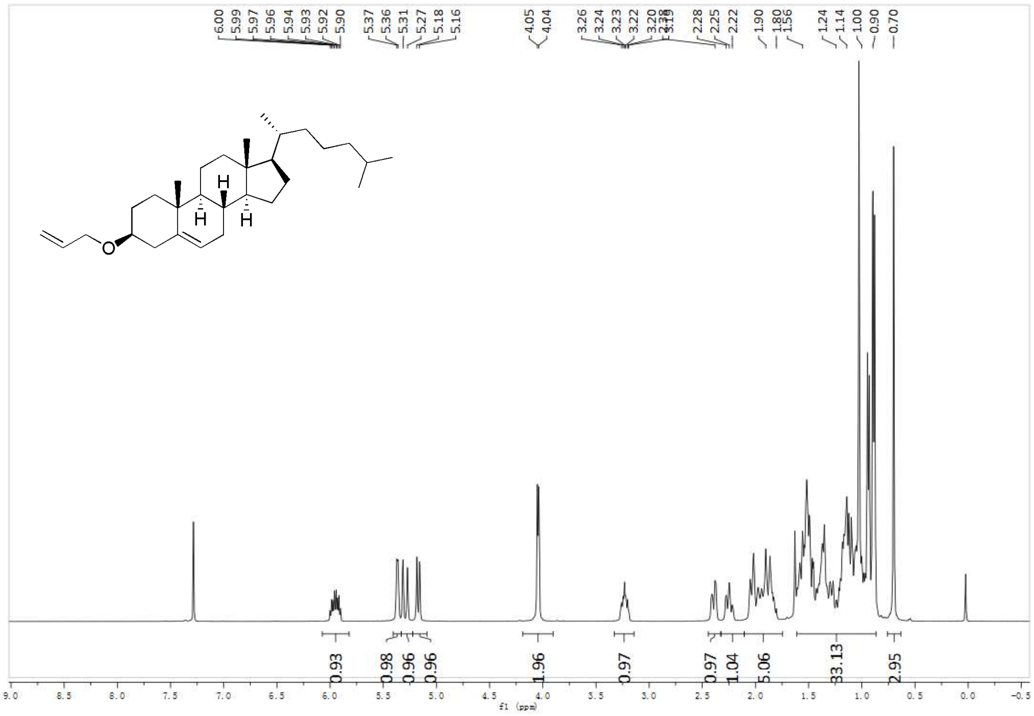
6.00
5.99
5.97
5.96
5.94
5.93
5.92
5.90
5.37
5.36
5.31
5.27
5.18
5.16
4.05
4.04
3.26
3.24
3.23
3.22
3.20
2.28
2.25
2.22
1.90
1.80
1.56
1.24
1.14
1.00
0.90
0.70
H
H
H
O
0.93
0.98
0.96
0.96
1.96
0.97
0.97
1.04
5.06
33.13
2.95
f1 (ppm)

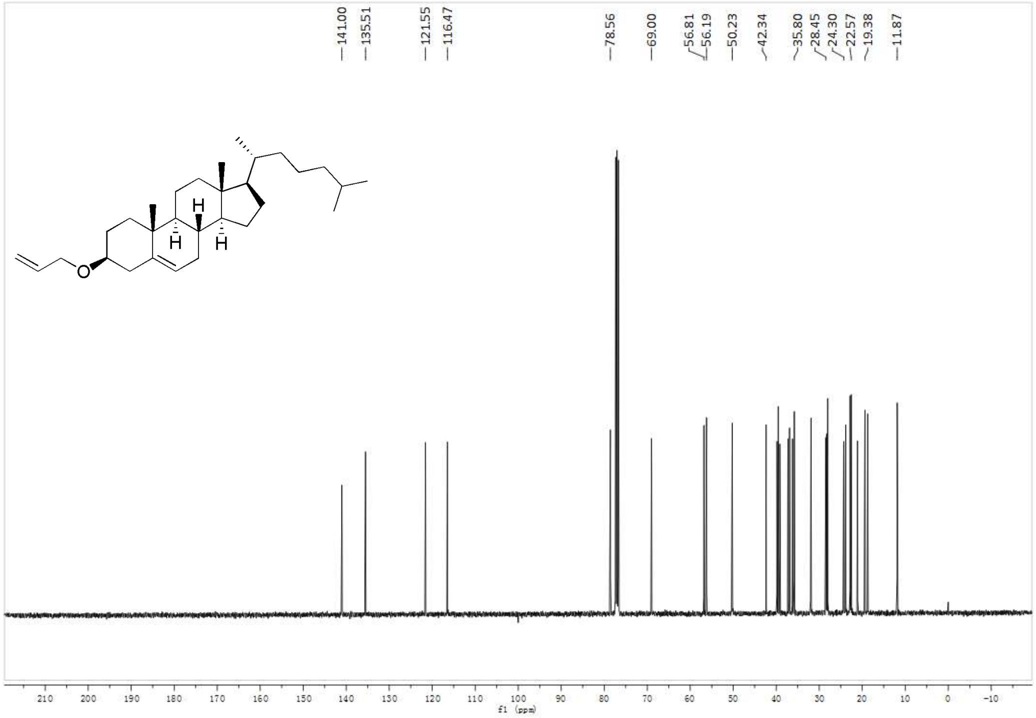
141.00
135.51
121.55
116.47
78.56
69.00
56.81
56.19
50.23
42.34
35.80
28.45
24.30
22.57
19.38
11.87
H
H
H
O
f1 (ppm)

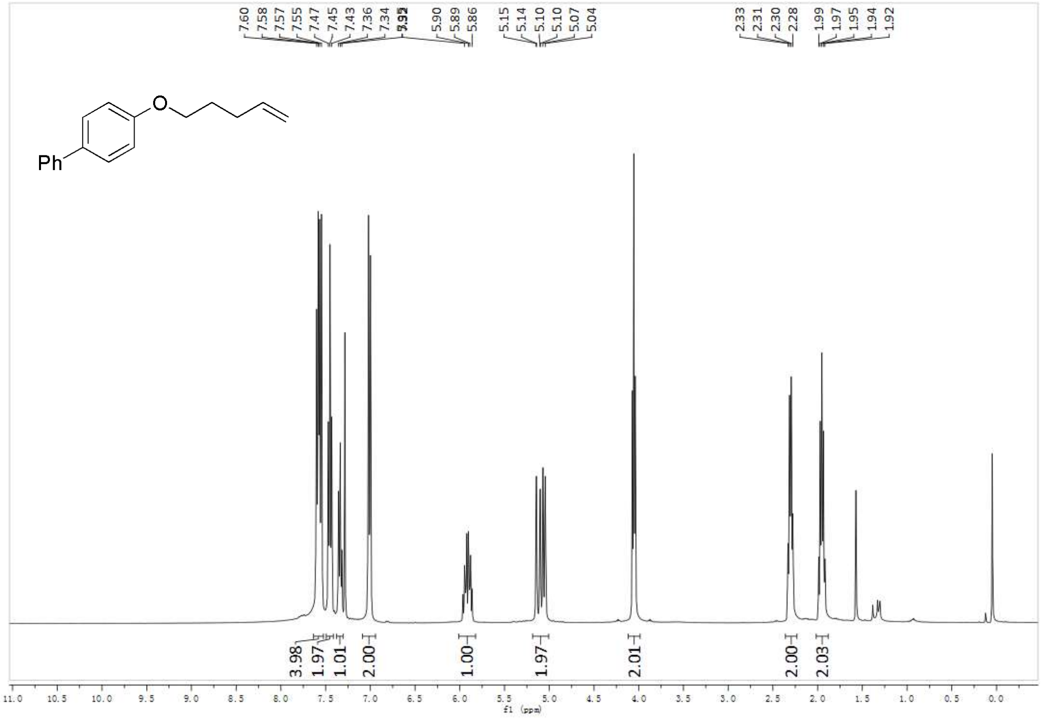
7.60
7.58
7.57
7.55
7.47
7.45
7.43
7.36
7.34
5.90
5.89
5.86
5.15
5.14
5.10
5.10
5.07
5.04
2.33
2.31
2.30
2.28
1.99
1.97
1.95
1.94
1.92
Ph
O
3.98
1.97
1.01
2.00
1.00
1.97
2.01
2.00
2.03
f1 (ppm)

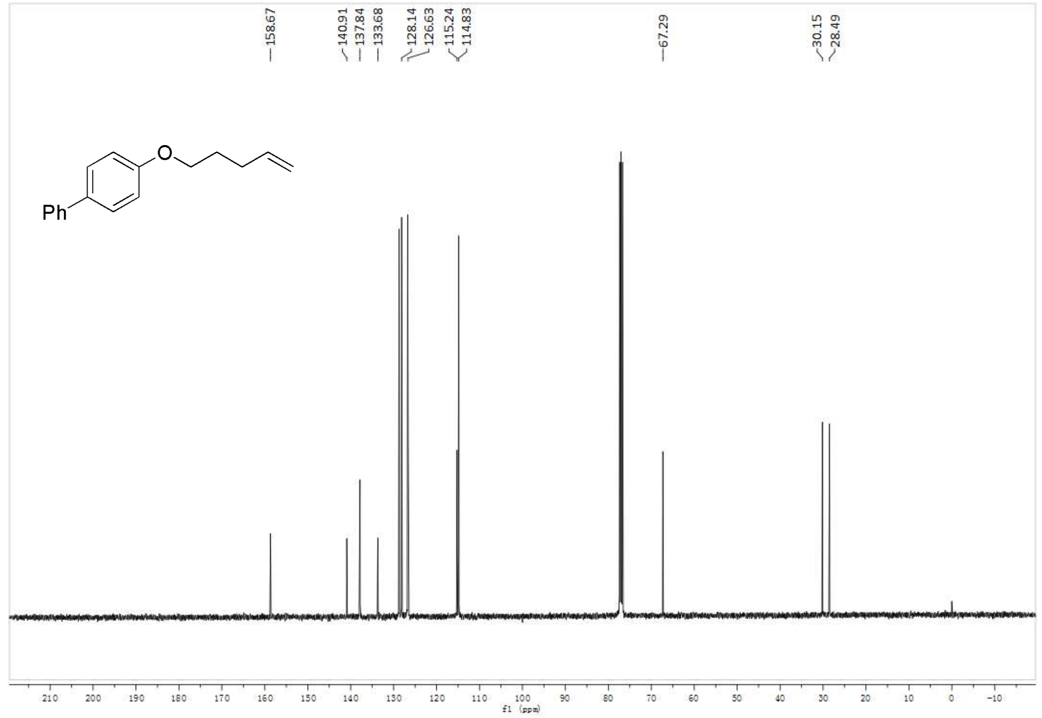
158.67
140.91
137.84
133.68
128.14
126.63
115.24
114.83
67.29
30.15
28.49
Ph
O
f1 (ppm)

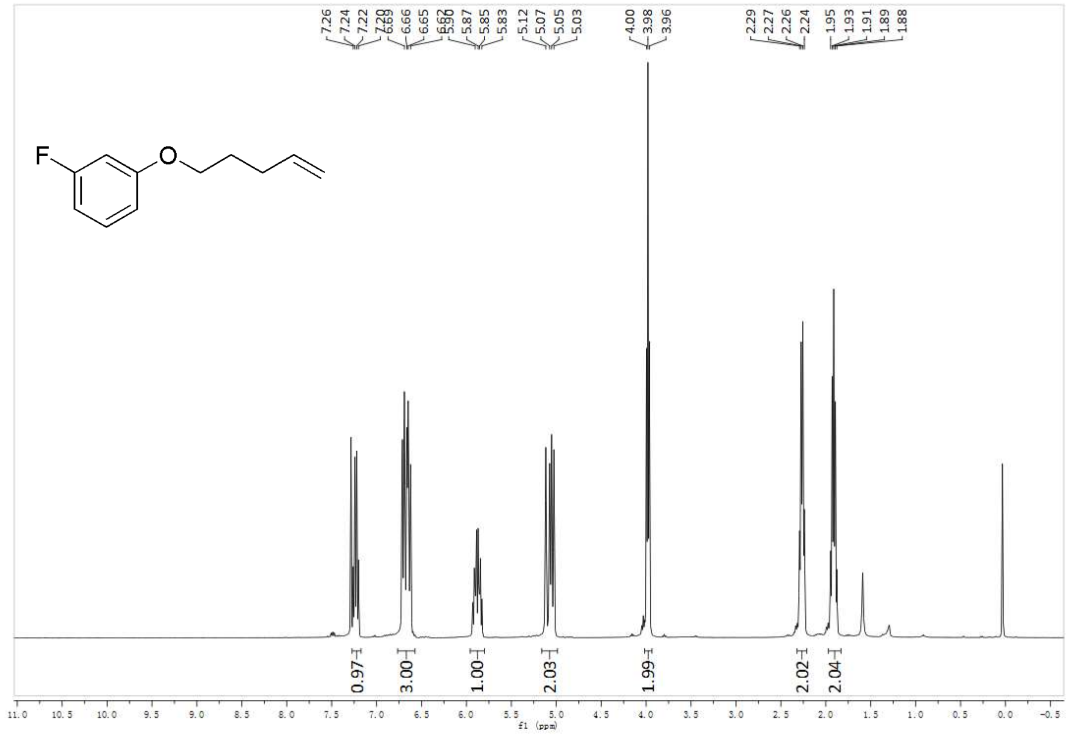
F
O
7.26
7.24
7.22
7.20
6.69
6.66
6.65
6.62
5.90
5.87
5.85
5.83
5.12
5.07
5.05
5.03
4.00
3.98
3.96
2.29
2.27
2.26
2.24
1.95
1.93
1.91
1.89
1.88
0.97
3.00
1.00
2.03
1.99
2.02
2.04
f1 (ppm)

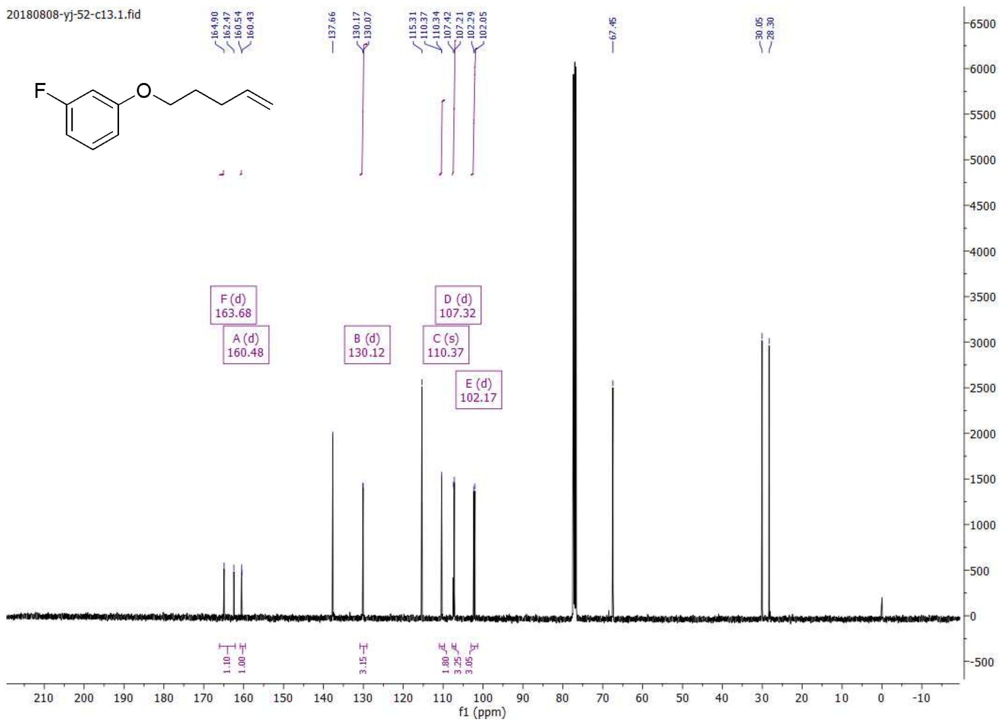
20180808-yj-52-c13.1.fid
164.90
162.47
160.54
160.43
137.66
130.17
130.07
115.31
110.37
110.34
107.42
107.21
102.29
102.05
67.45
30.05
28.30
F
O
F (d)
163.68
A (d)
160.48
B (d)
130.12
C (s)
110.37
D (d)
107.32
E (d)
102.17
1.10
1.00
3.15
1.80
3.25
3.05
f1 (ppm)

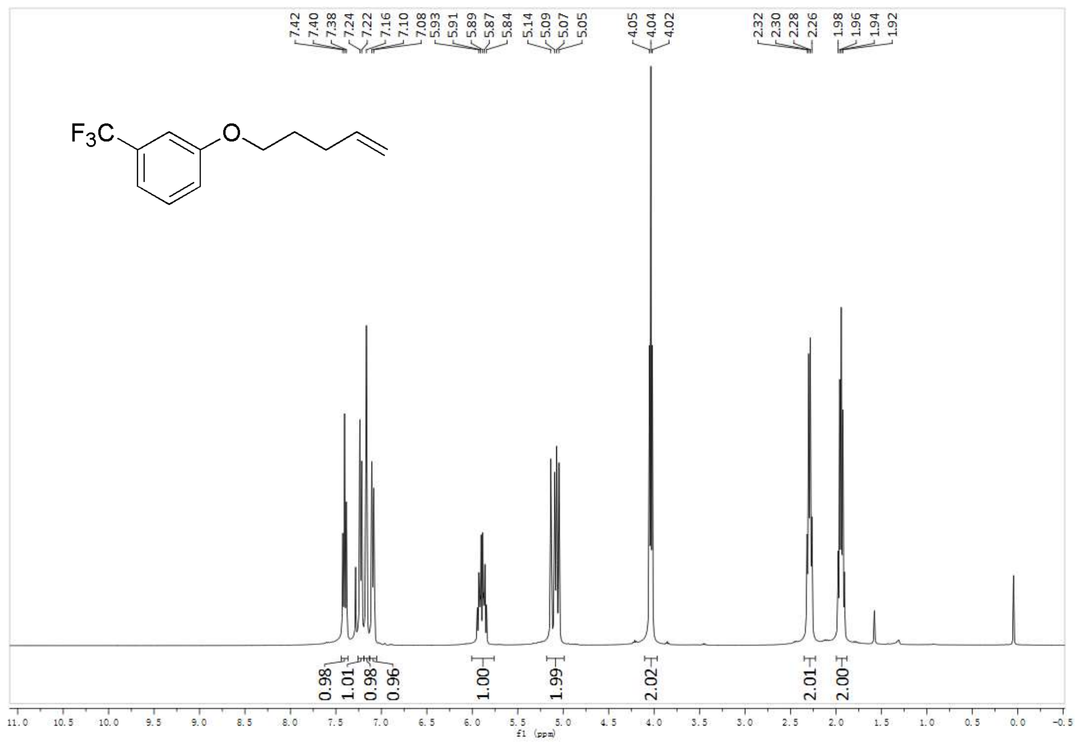

7.42 7.40 7.38 7.24 7.22 7.16 7.10 7.08 5.93 5.91 5.89 5.87 5.84 5.14 5.09 5.07 5.05 4.05 4.04 4.02 2.32 2.30 2.28 2.26 1.98 1.96 1.94 1.92
F_3C
O
0.98 1.01 0.98 0.96 1.00 1.99 2.02 2.01 2.00
f1 (ppm)

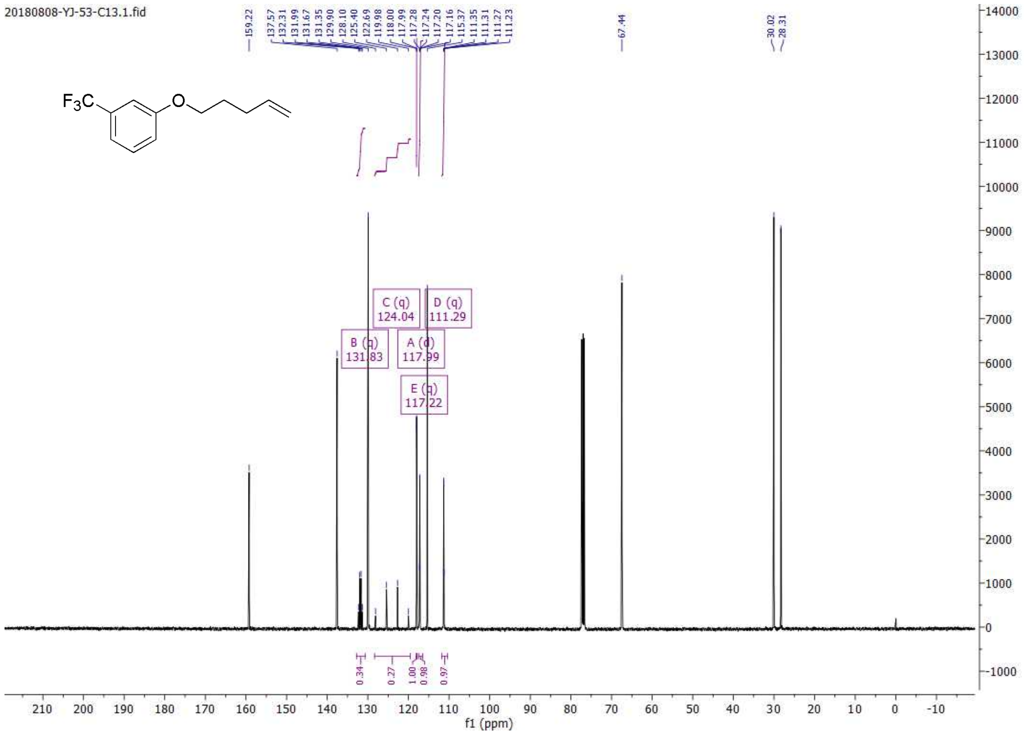

20180808-YJ-53-C13.1.fid
159.22 137.57 132.31 131.99 131.67 131.35 129.90 128.10 125.40 122.69 119.98 118.00 117.99 117.28 117.24 117.20 117.16 115.37 111.35 111.31 111.27 111.23
67.44
30.02 28.31
F_3C
O
C (q) 124.04
D (q) 111.29
B (q) 131.83
A (q) 117.99
E (q) 117.22
0.34 0.27 1.00 0.98 0.97
f1 (ppm)

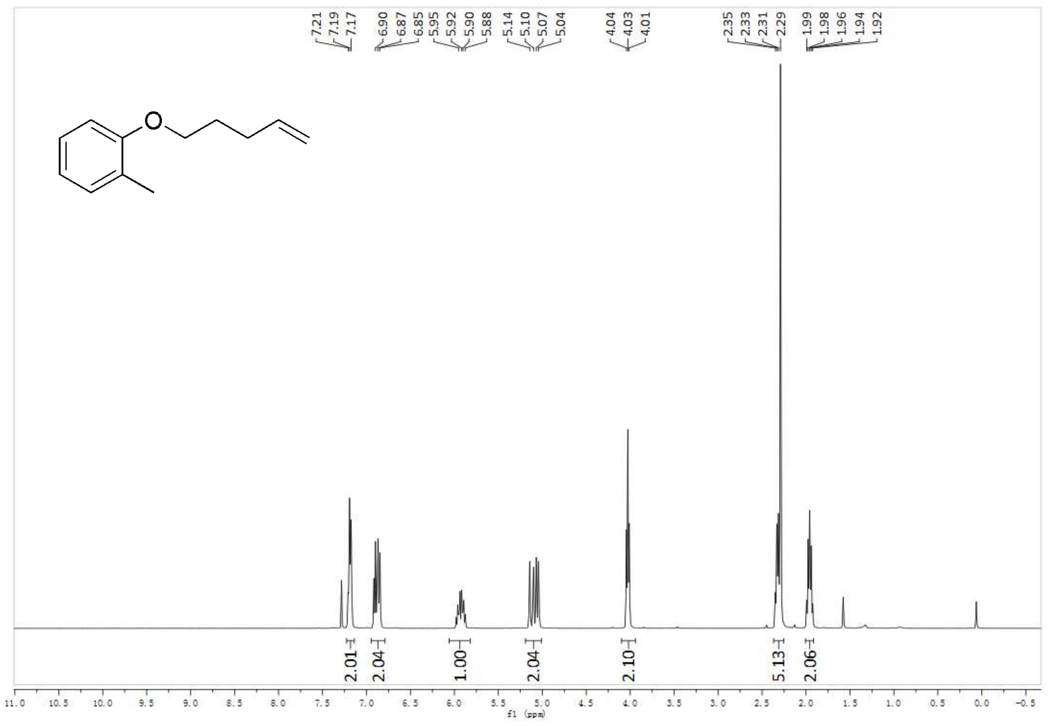

7.21
7.19
7.17
6.90
6.87
6.85
5.95
5.92
5.90
5.88
5.14
5.10
5.07
5.04
4.04
4.03
4.01
2.35
2.33
2.31
2.29
1.99
1.98
1.96
1.94
1.92
O
2.01
2.04
1.00
2.04
2.10
5.13
2.06
f1 (ppm)

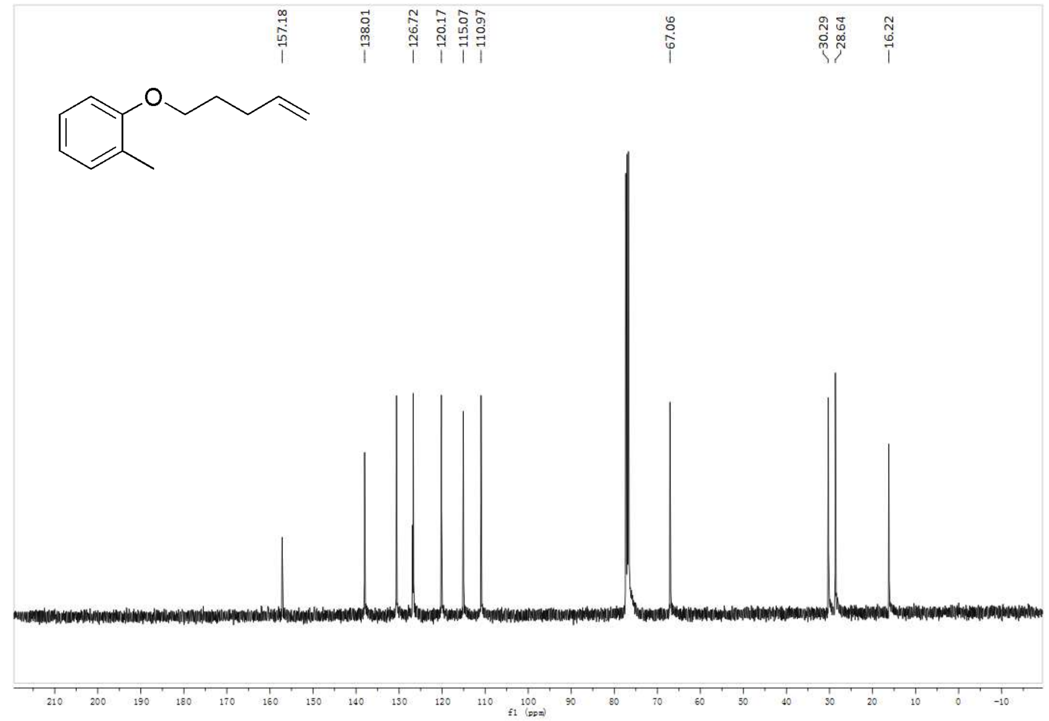

157.18
138.01
126.72
120.17
115.07
110.97
67.06
30.29
28.64
16.22
O
f1 (ppm)

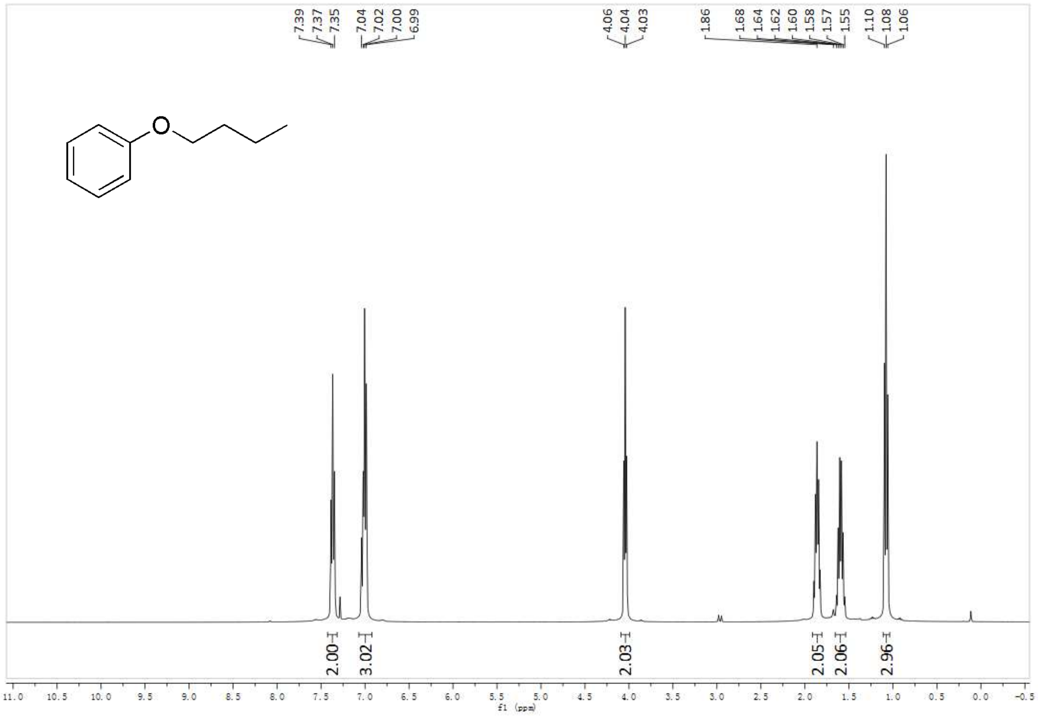

7.39
7.37
7.35
7.04
7.02
7.00
6.99
4.06
4.04
4.03
1.86
1.68
1.64
1.62
1.60
1.58
1.57
1.55
1.10
1.08
1.06
O
2.00
3.02
2.03
2.05
2.06
2.96
11.0 10.5 10.0 9.5 9.0 8.5 8.0 7.5 7.0 6.5 6.0 5.5 5.0 4.5 4.0 3.5 3.0 2.5 2.0 1.5 1.0 0.5 0.0 -0.5
f1 (ppm)

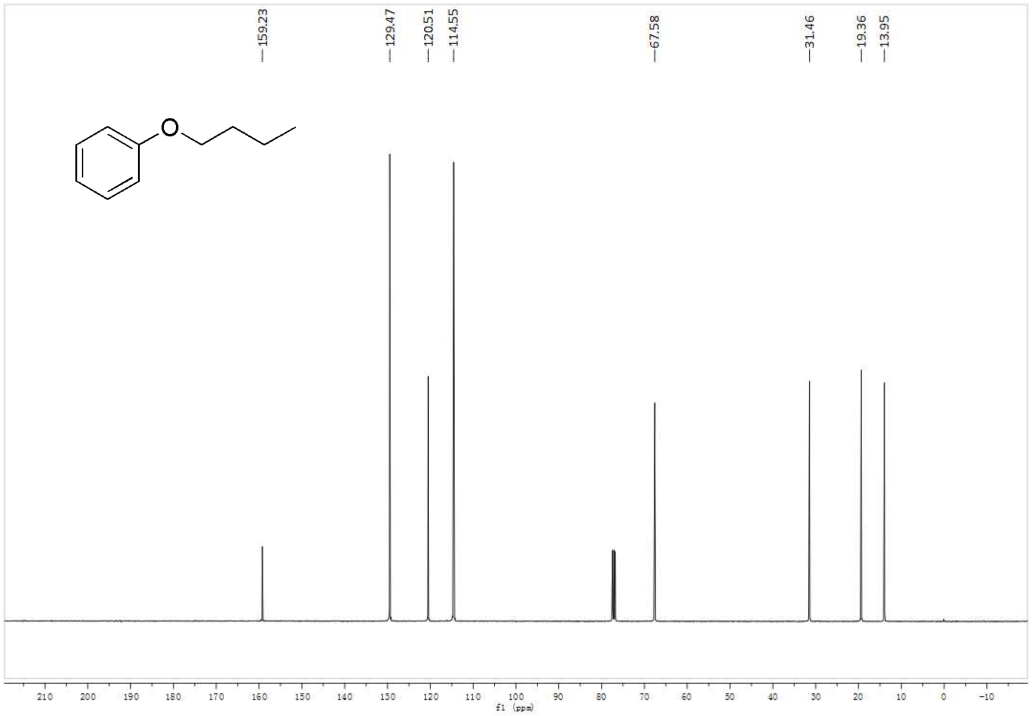

159.23
129.47
120.51
114.55
67.58
31.46
19.36
13.95
O
210 200 190 180 170 160 150 140 130 120 110 100 90 80 70 60 50 40 30 20 10 0 -10
f1 (ppm)

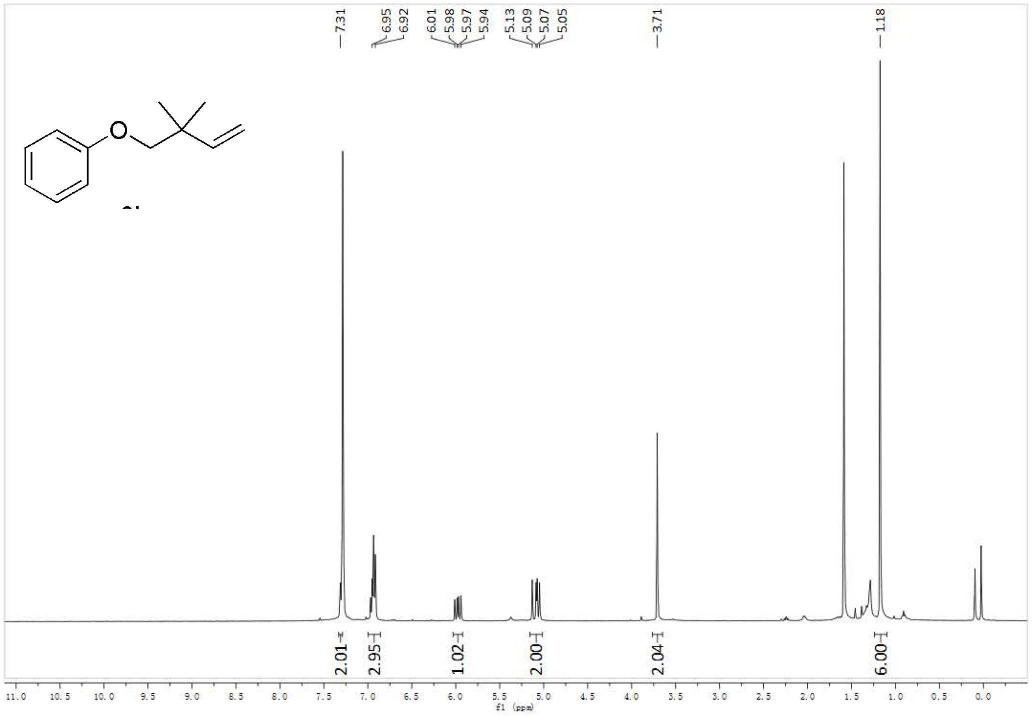
7.31
6.95
6.92
6.01
5.98
5.97
5.94
5.13
5.09
5.07
5.05
3.71
1.18
O
2.01
2.95
1.02
2.00
2.04
6.00
11.0 10.5 10.0 9.5 9.0 8.5 8.0 7.5 7.0 6.5 6.0 5.5 5.0 4.5 4.0 3.5 3.0 2.5 2.0 1.5 1.0 0.5 0.0
f1 (ppm)

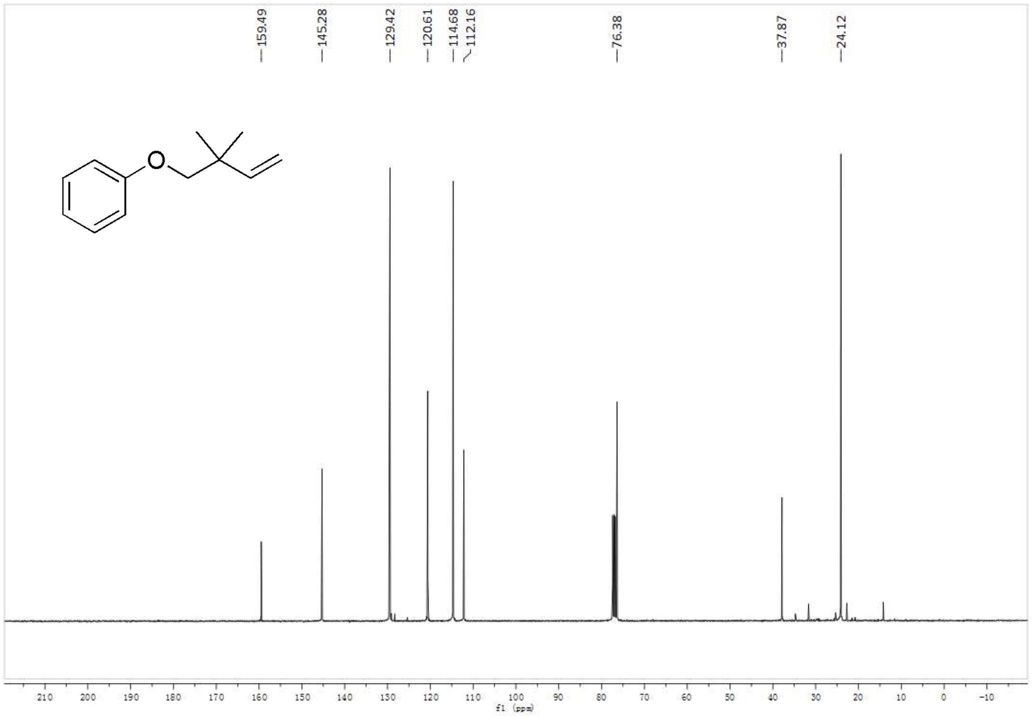
159.49
145.28
129.42
120.61
114.68
112.16
76.38
37.87
24.12
O
210 200 190 180 170 160 150 140 130 120 110 100 90 80 70 60 50 40 30 20 10 0 -10
f1 (ppm)

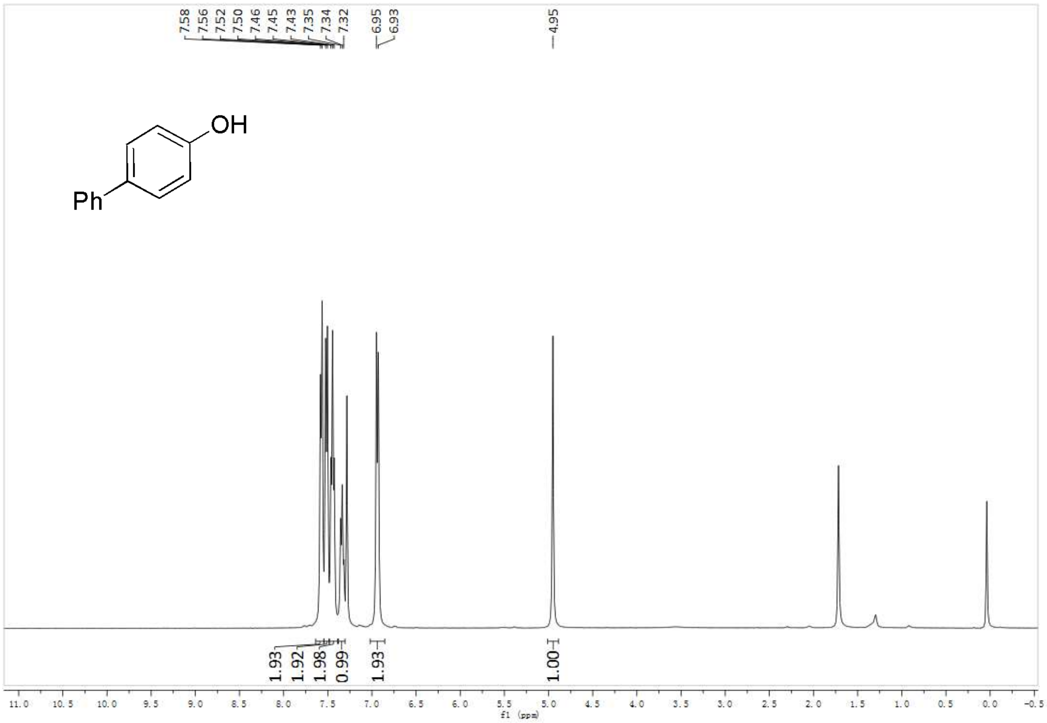
7.58
7.56
7.52
7.50
7.46
7.45
7.43
7.35
7.34
7.32
6.95
6.93
4.95
OH
Ph
1.93
1.92
1.98
0.99
1.93
1.00
f1 (ppm)

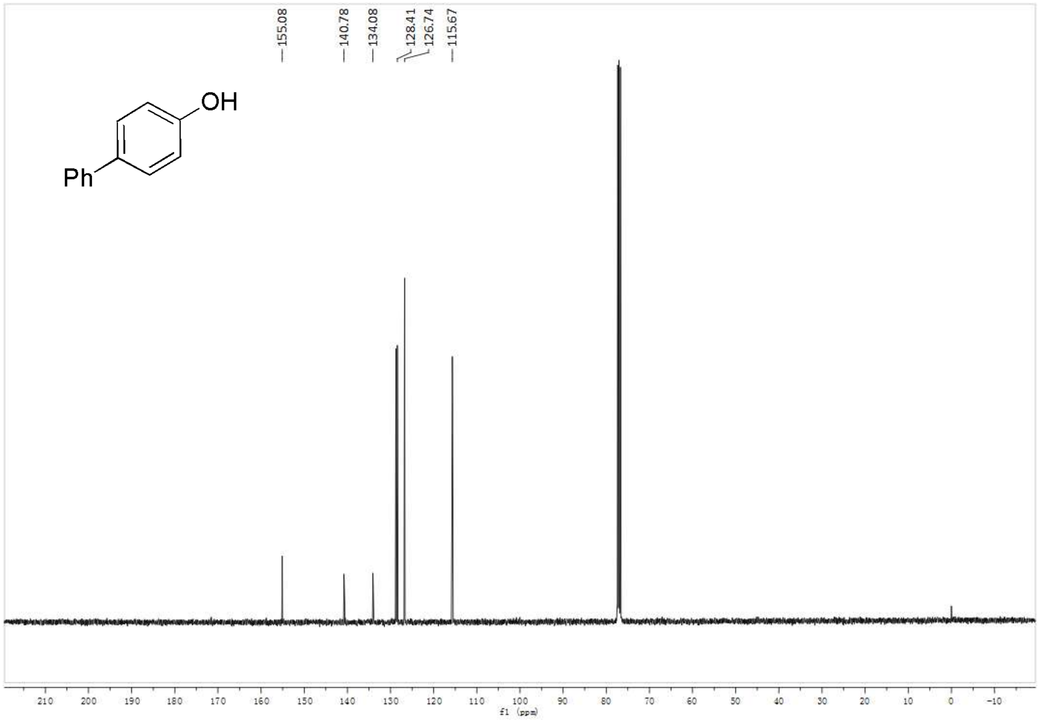
155.08
140.78
134.08
128.41
126.74
115.67
OH
Ph
f1 (ppm)

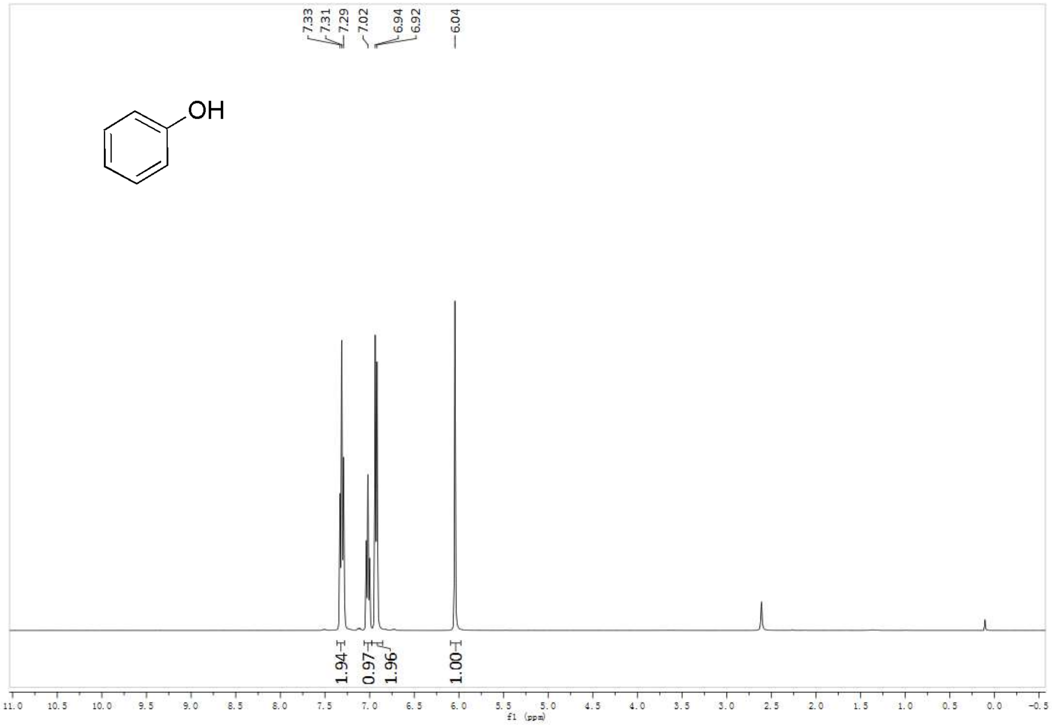
7.33
7.31
7.29
7.02
6.94
6.92
6.04
OH
1.94
0.97
1.96
1.00
11.0 10.5 10.0 9.5 9.0 8.5 8.0 7.5 7.0 6.5 6.0 5.5 5.0 4.5 4.0 3.5 3.0 2.5 2.0 1.5 1.0 0.5 0.0 -0.5
f1 (ppm)

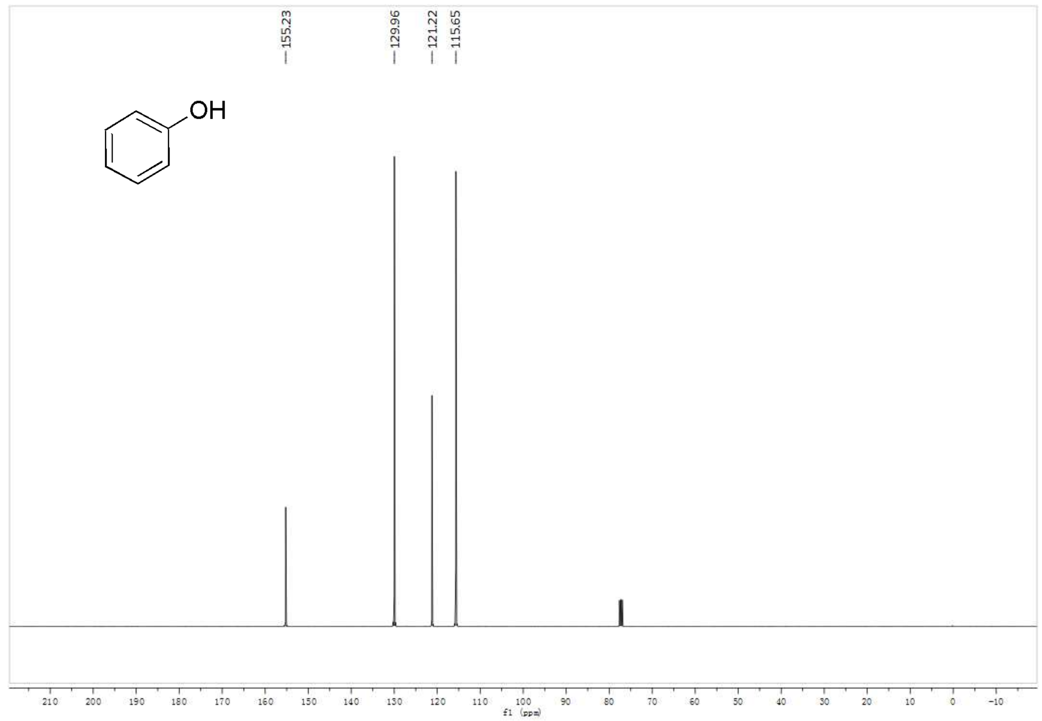
155.23
129.96
121.22
115.65
OH
210 200 190 180 170 160 150 140 130 120 110 100 90 80 70 60 50 40 30 20 10 0 -10
f1 (ppm)

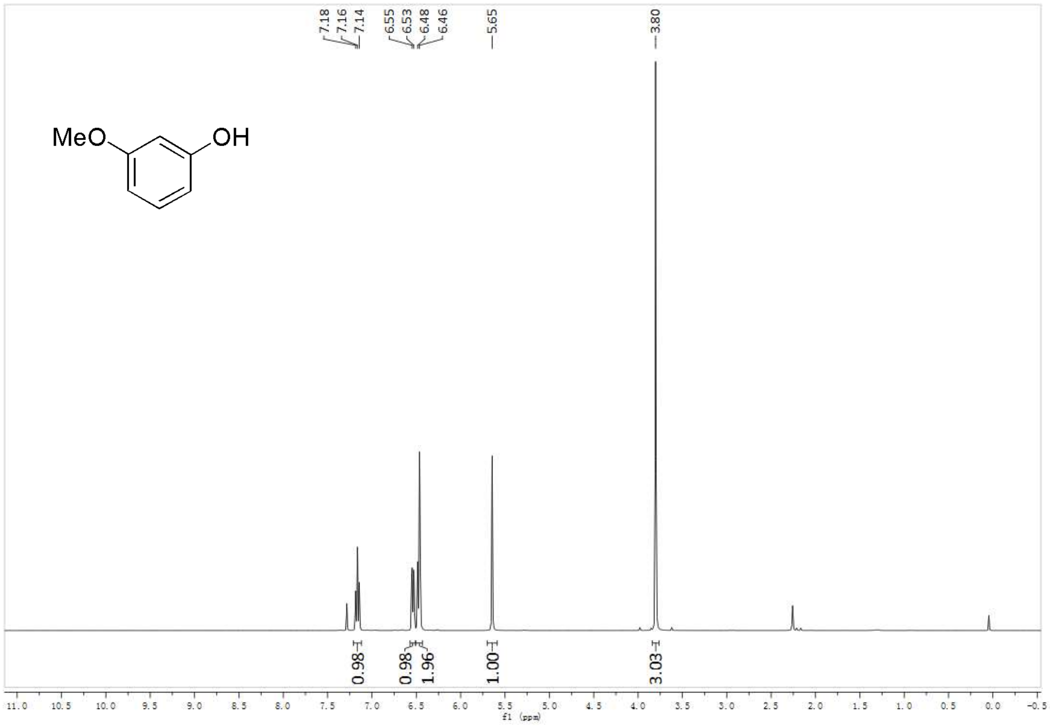
7.18
7.16
7.14
6.55
6.53
6.48
6.46
5.65
3.80
MeO
OH
0.98
0.98
1.96
1.00
3.03
f1 (ppm)

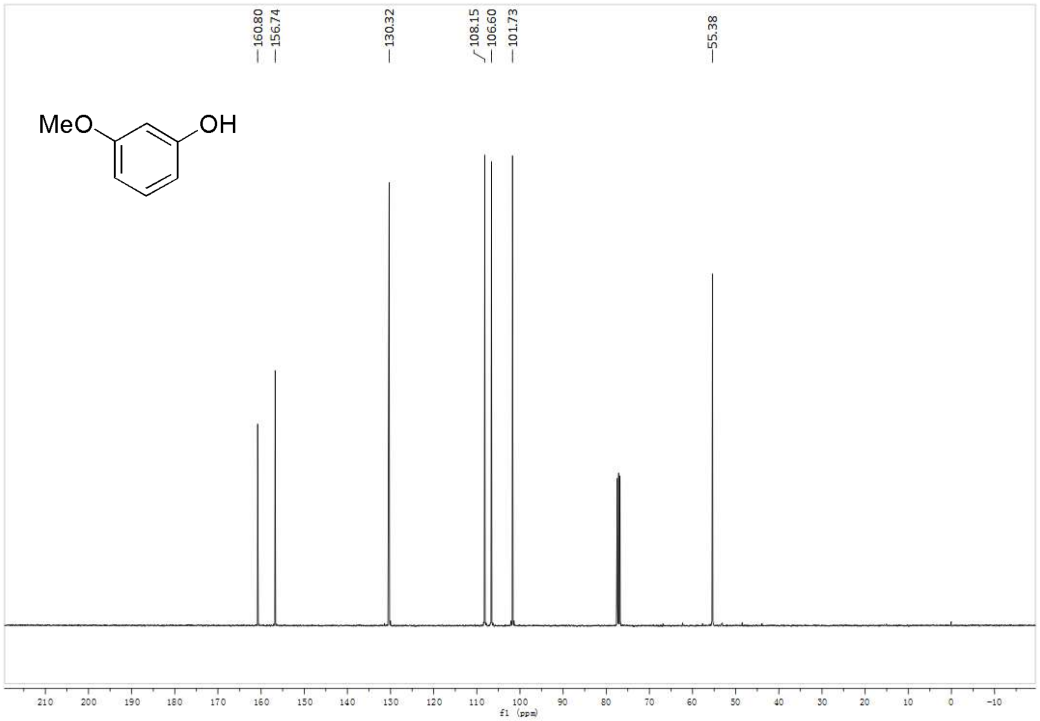
160.80
156.74
130.32
108.15
106.60
101.73
55.38
MeO
OH
f1 (ppm)

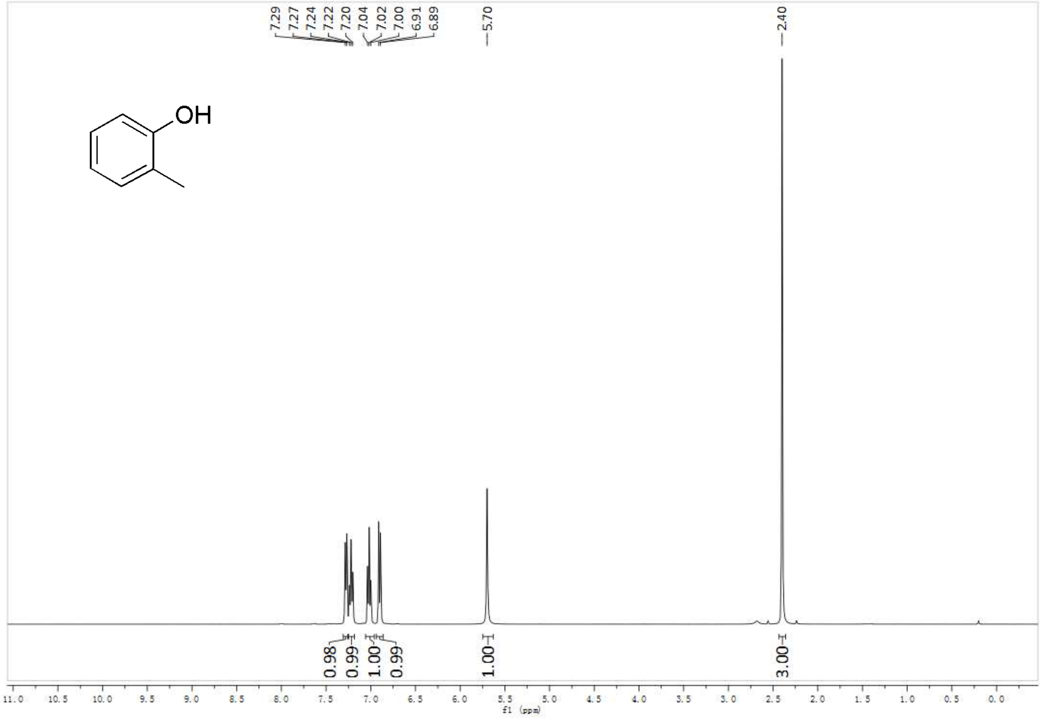

7.29
7.27
7.24
7.22
7.20
7.04
7.02
7.00
6.91
6.89
5.70
2.40
OH
0.98
0.99
1.00
0.99
1.00
3.00
11.0 10.5 10.0 9.5 9.0 8.5 8.0 7.5 7.0 6.5 6.0 5.5 5.0 4.5 4.0 3.5 3.0 2.5 2.0 1.5 1.0 0.5 0.0
f1 (ppm)

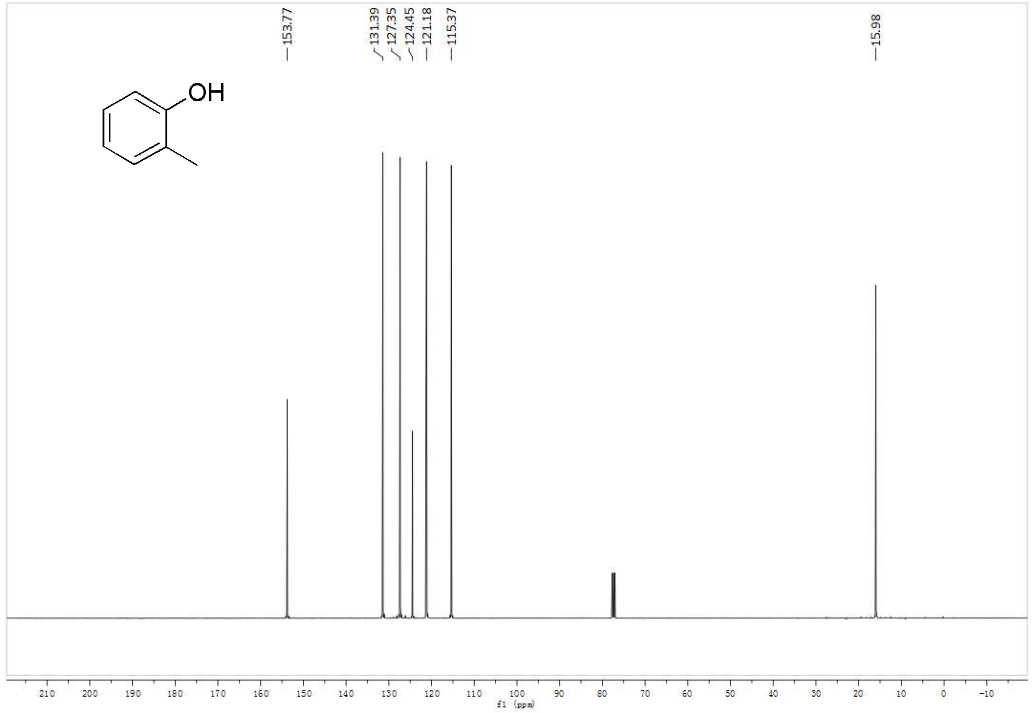

153.77
131.39
127.35
124.45
121.18
115.37
15.98
OH
210 200 190 180 170 160 150 140 130 120 110 100 90 80 70 60 50 40 30 20 10 0 -10
f1 (ppm)

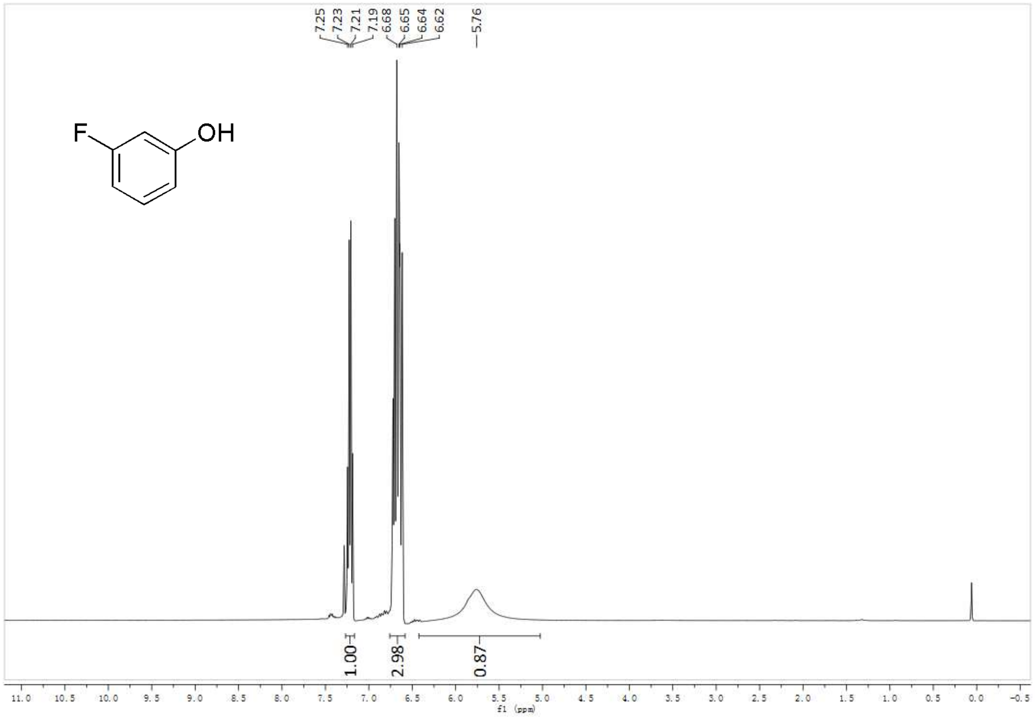
7.25
7.23
7.21
7.19
6.68
6.65
6.64
6.62
5.76
F
OH
1.00
2.98
0.87
11.0 10.5 10.0 9.5 9.0 8.5 8.0 7.5 7.0 6.5 6.0 5.5 5.0 4.5 4.0 3.5 3.0 2.5 2.0 1.5 1.0 0.5 0.0 -0.5
f1 (ppm)

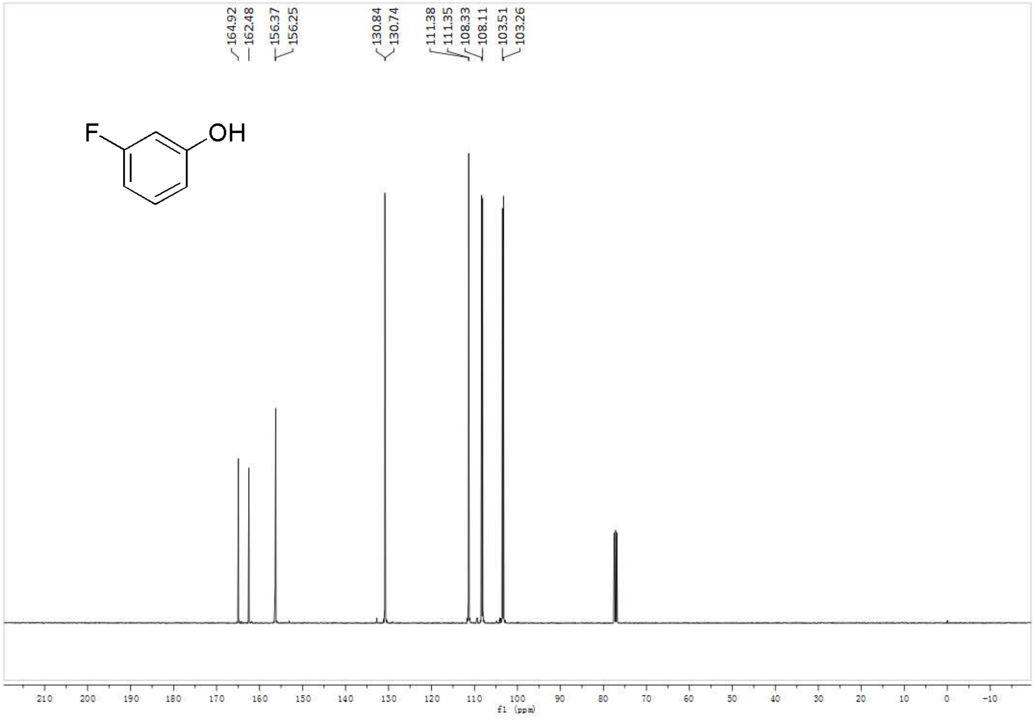
164.92
162.48
156.37
156.25
130.84
130.74
111.38
111.35
108.33
108.11
103.51
103.26
F
OH
210 200 190 180 170 160 150 140 130 120 110 100 90 80 70 60 50 40 30 20 10 0 -10
f1 (ppm)

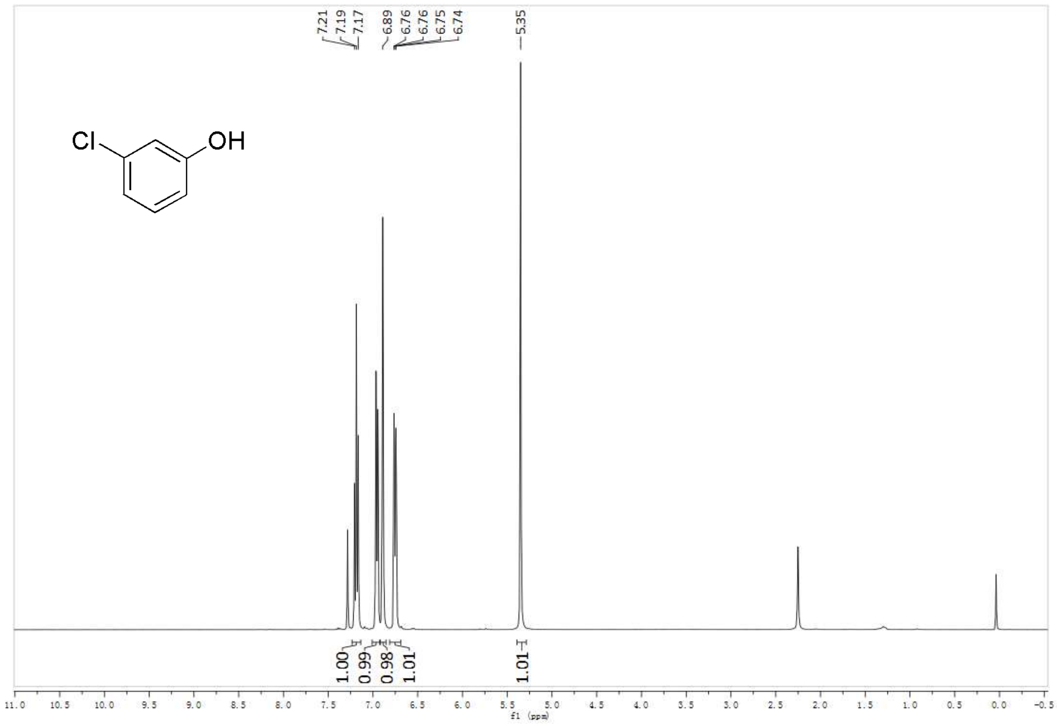
7.21
7.19
7.17
6.89
6.76
6.76
6.75
6.74
5.35
Cl
OH
1.00
0.99
0.98
1.01
1.01
f1 (ppm)

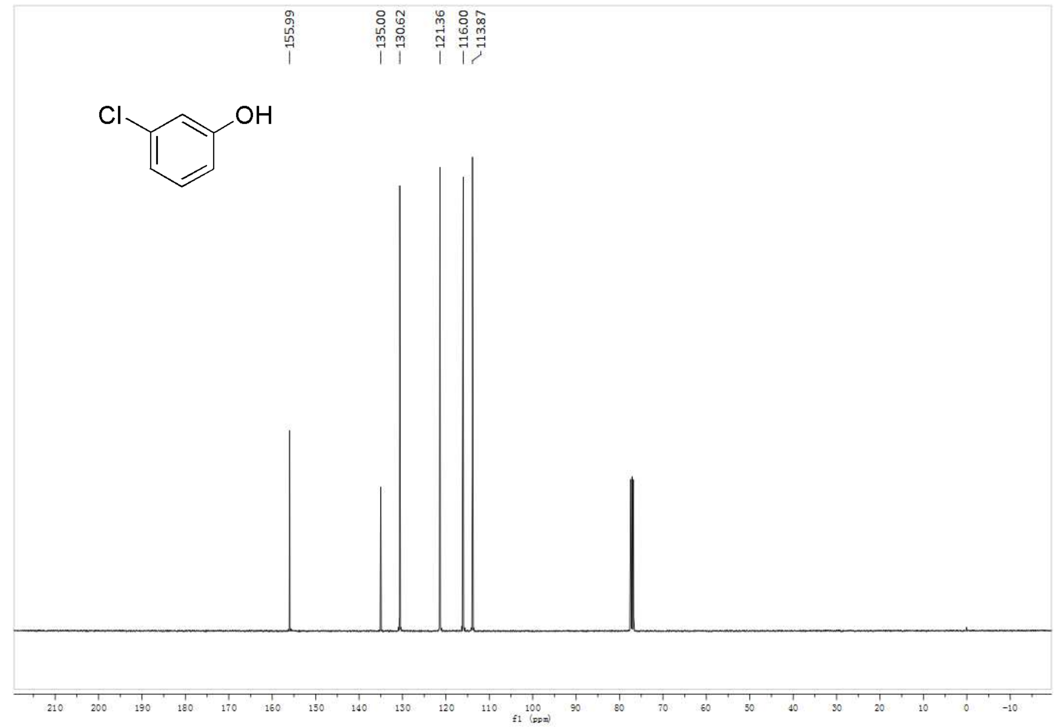
155.99
135.00
130.62
121.36
116.00
113.87
Cl
OH
f1 (ppm)

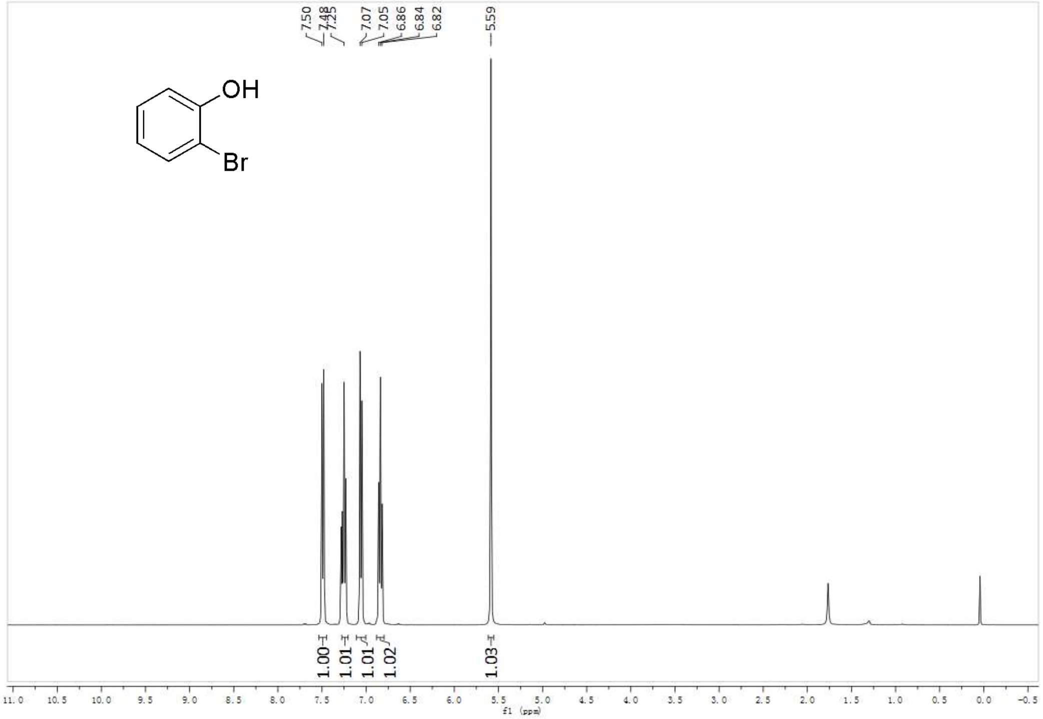
7.50
7.48
7.25
7.07
7.05
6.86
6.84
6.82
5.59
OH
Br
1.00
1.01
1.01
1.02
1.03
f1 (ppm)

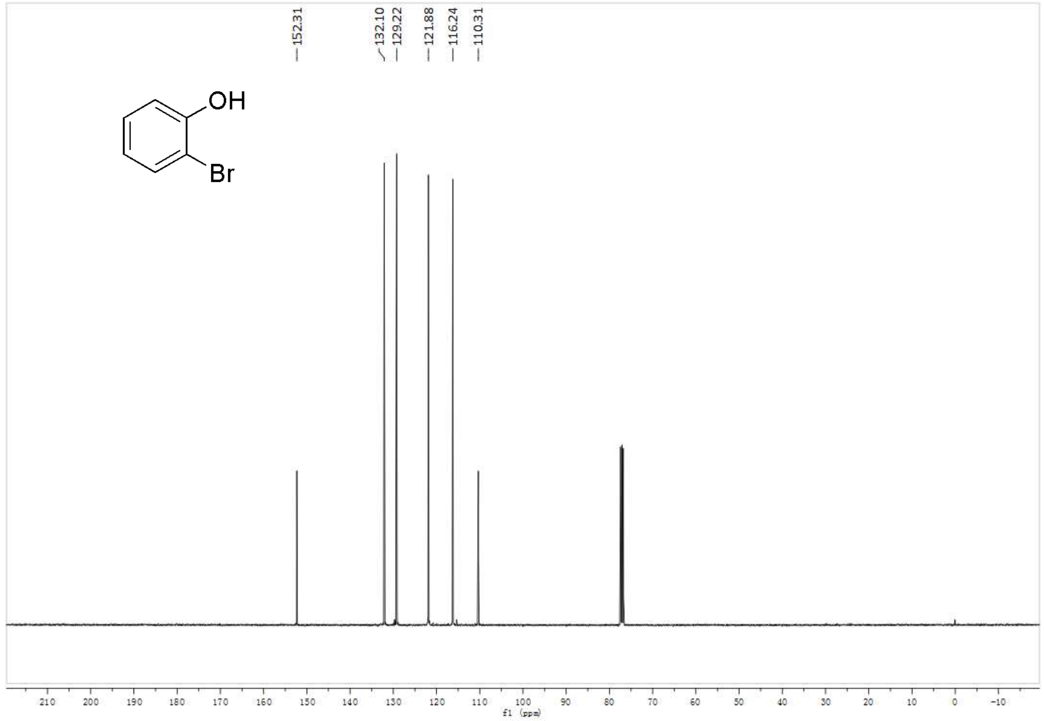
152.31
132.10
129.22
121.88
116.24
110.31
OH
Br
f1 (ppm)

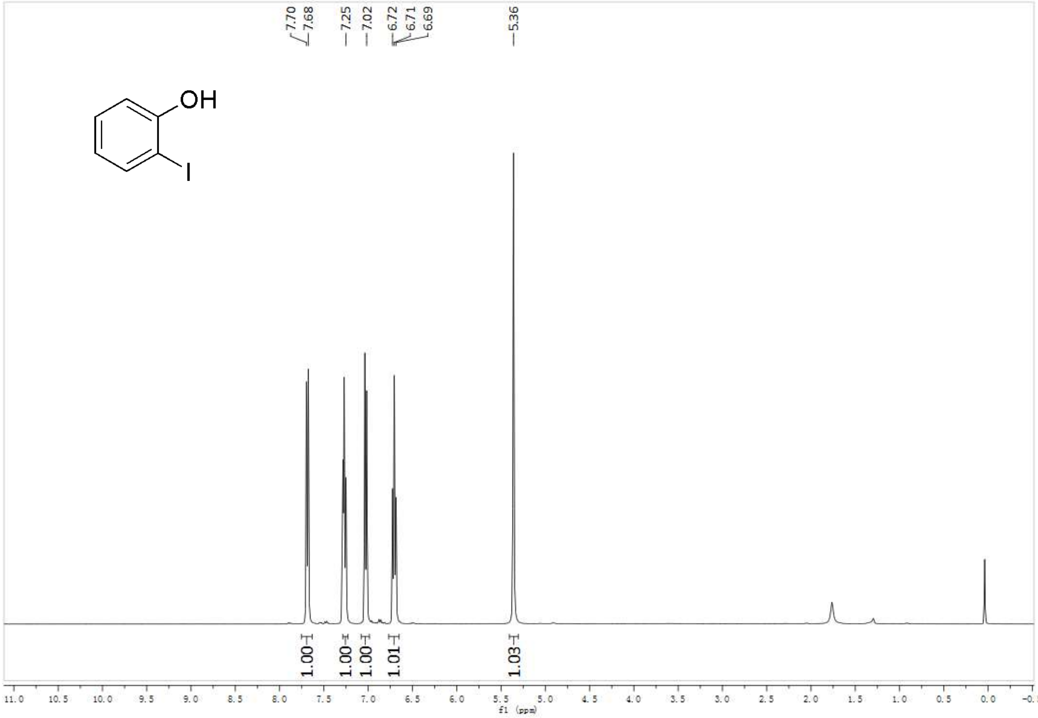
7.70
7.68
7.25
7.02
6.72
6.71
6.69
5.36
OH
I
1.00
1.00
1.00
1.01
1.03
f1 (ppm)

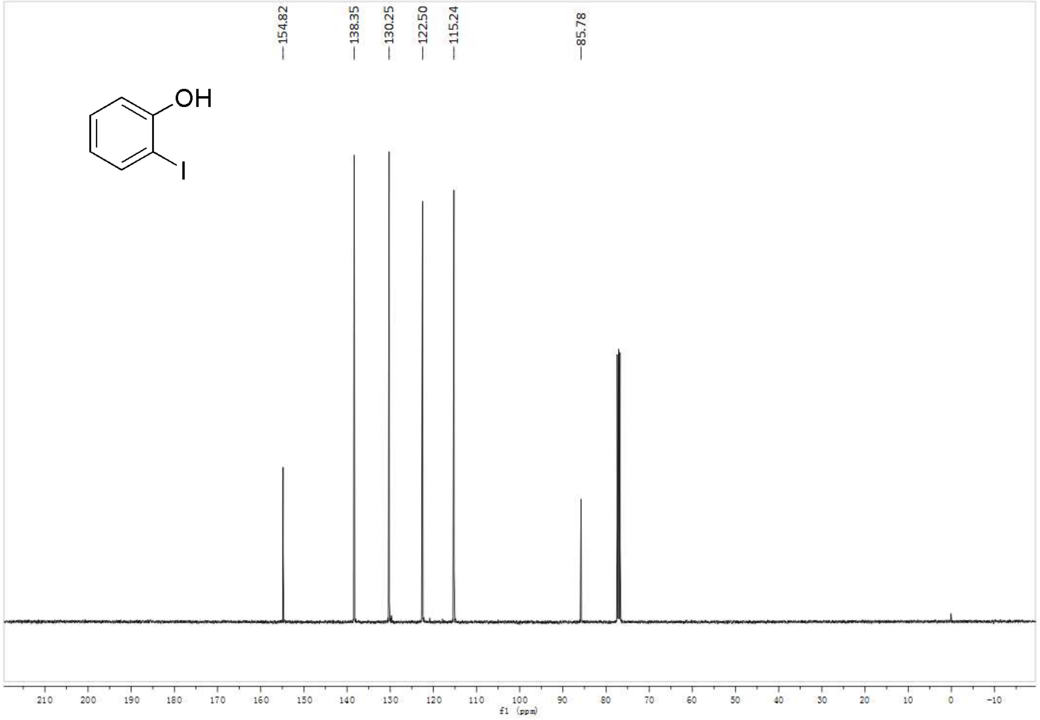
154.82
138.35
130.25
122.50
115.24
85.78
OH
I
f1 (ppm)

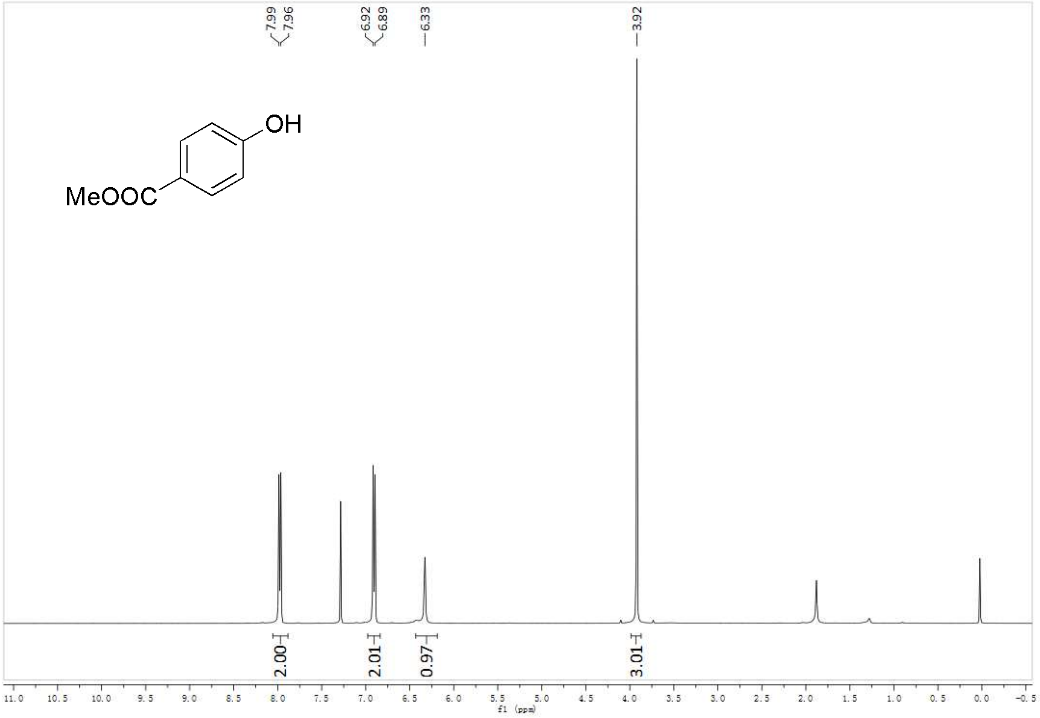
7.99
7.96
6.92
6.89
6.33
3.92
OH
MeOOC
2.00
2.01
0.97
3.01
f1 (ppm)

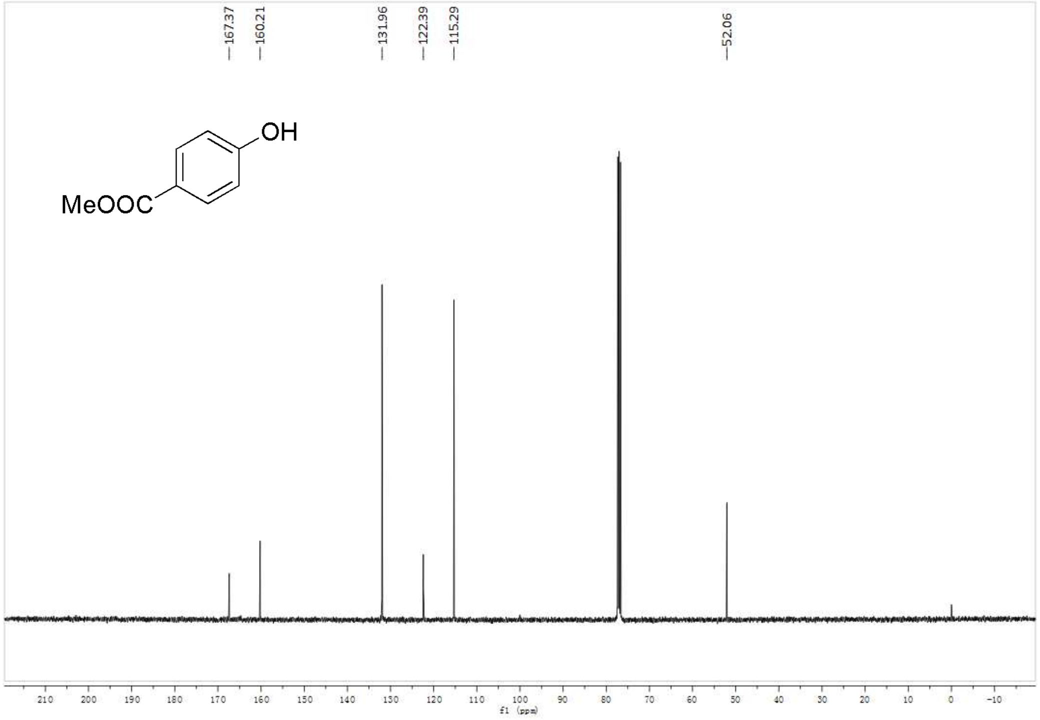
167.37
160.21
131.96
122.39
115.29
52.06
OH
MeOOC
f1 (ppm)

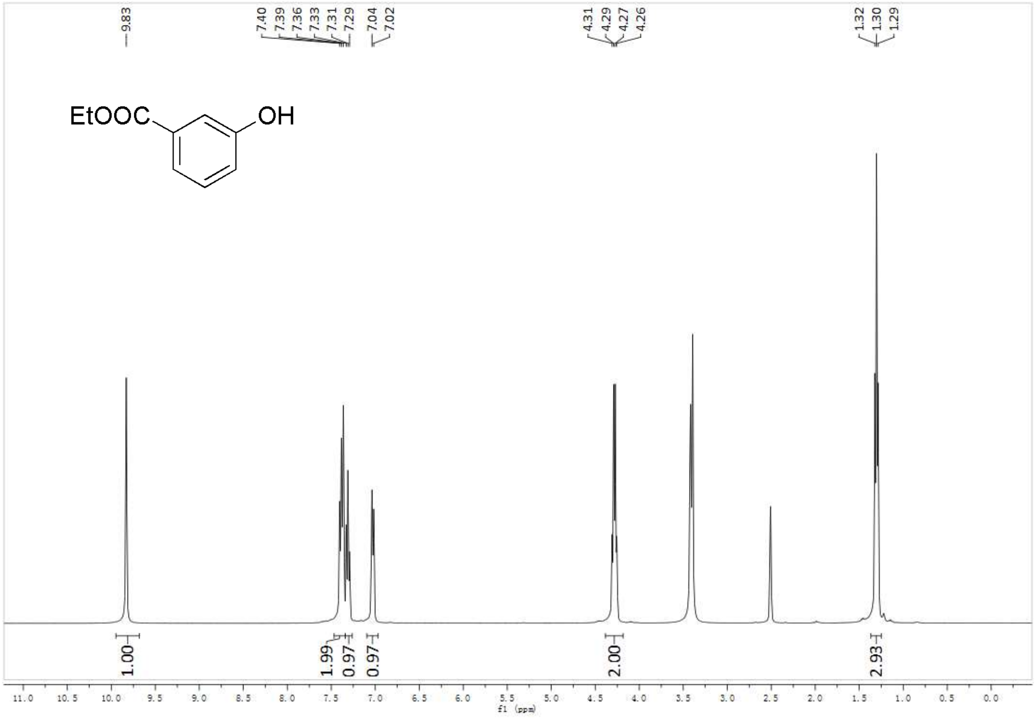

9.83
7.40
7.39
7.36
7.33
7.31
7.29
7.04
7.02
4.31
4.29
4.27
4.26
1.32
1.30
1.29
EtOOC
OH
1.00
1.99
0.97
0.97
2.00
2.93
f1 (ppm)

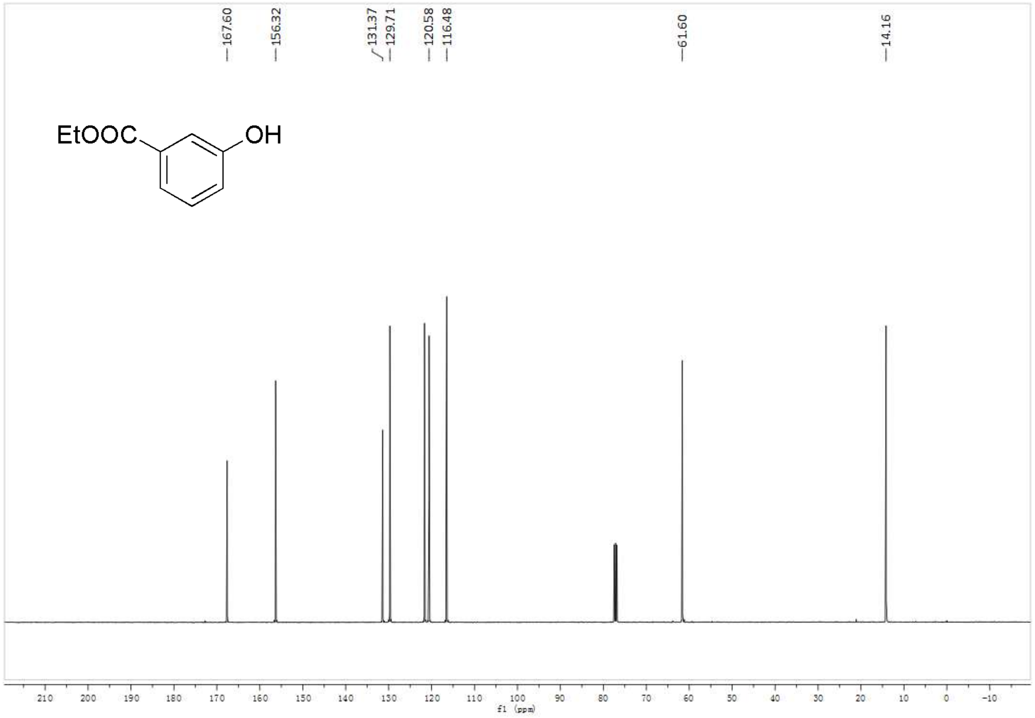

167.60
156.32
131.37
129.71
120.58
116.48
61.60
14.16
EtOOC
OH
f1 (ppm)

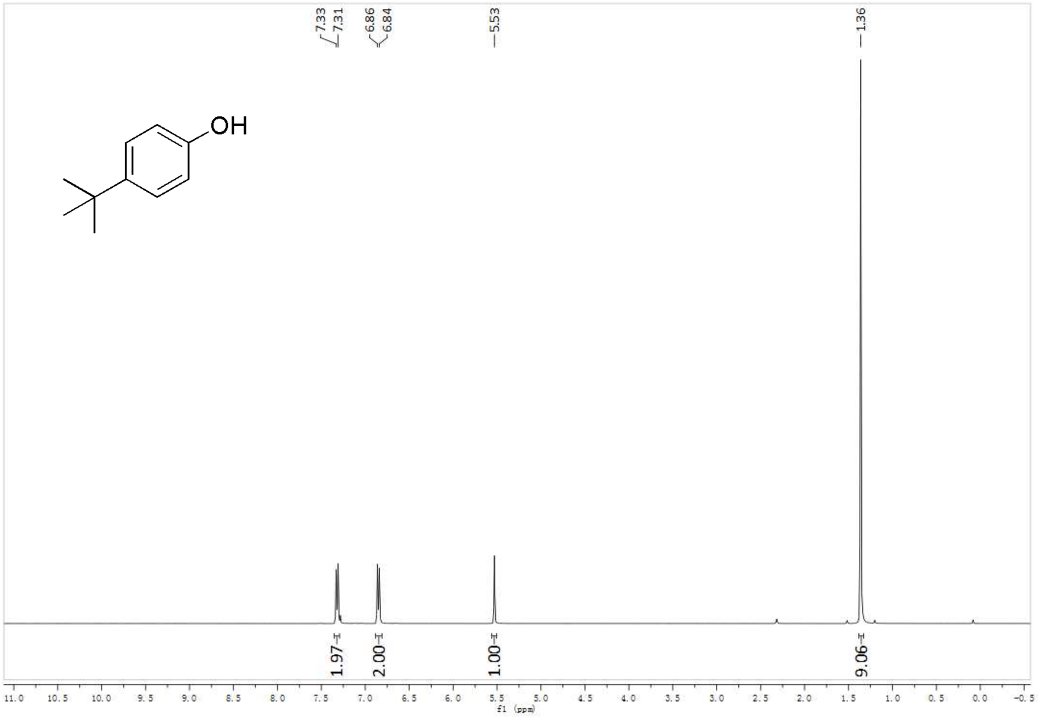

7.33
7.31
6.86
6.84
5.53
1.36
OH
1.97
2.00
1.00
9.06
11.0 10.5 10.0 9.5 9.0 8.5 8.0 7.5 7.0 6.5 6.0 5.5 5.0 4.5 4.0 3.5 3.0 2.5 2.0 1.5 1.0 0.5 0.0 -0.5
f1 (ppm)

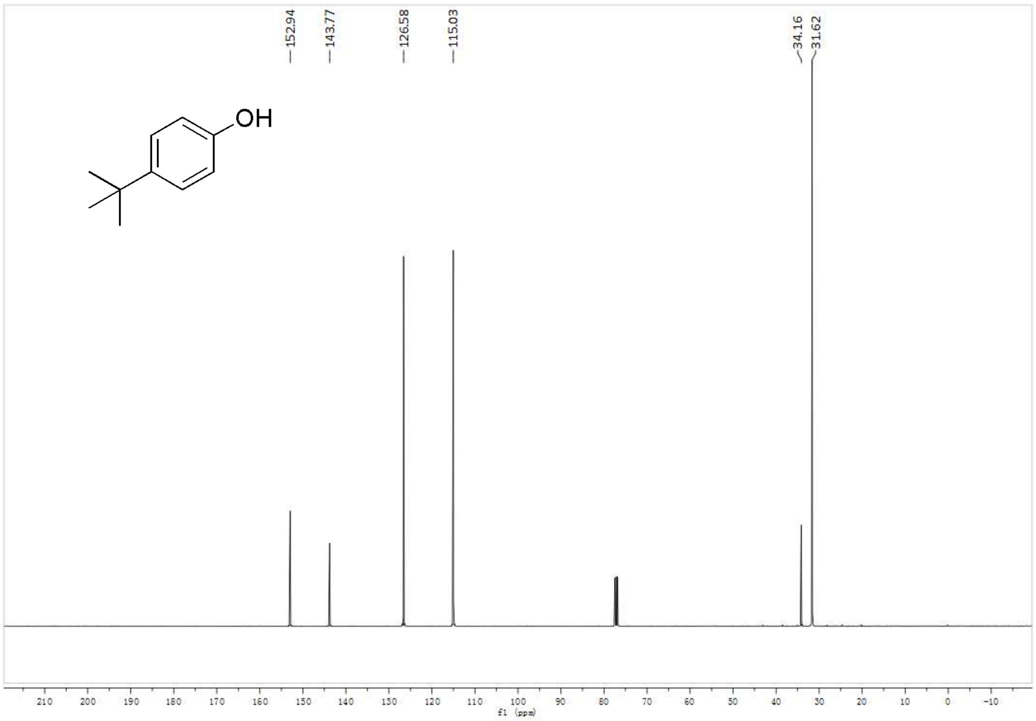

152.94
143.77
126.58
115.03
34.16
31.62
OH
210 200 190 180 170 160 150 140 130 120 110 100 90 80 70 60 50 40 30 20 10 0 -10
f1 (ppm)

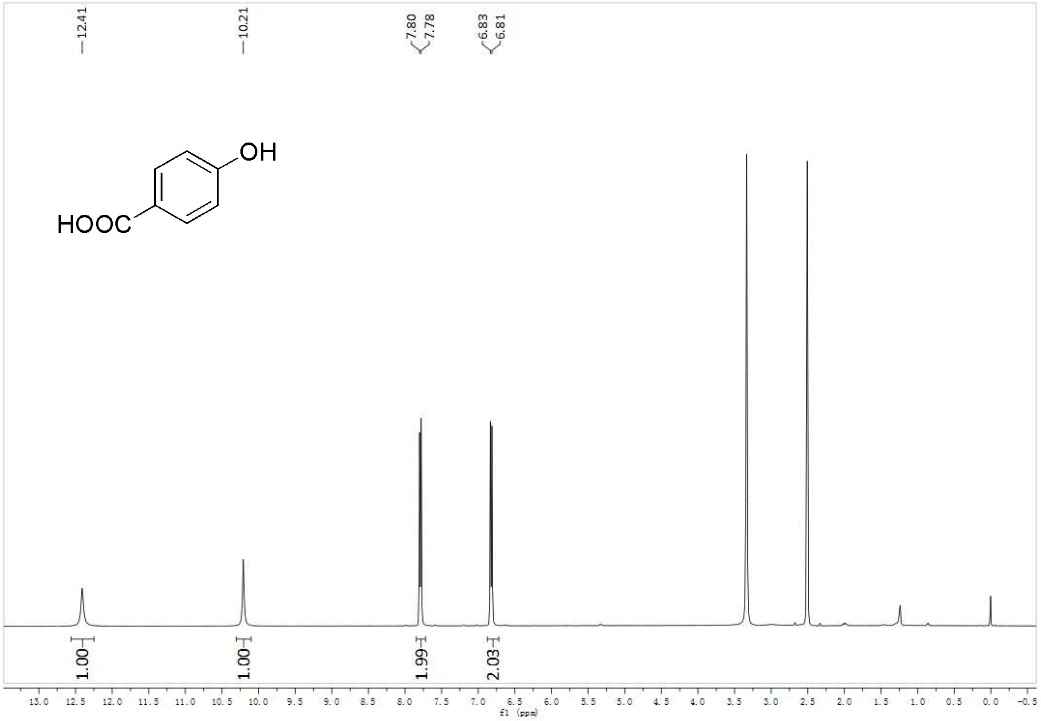

12.41
10.21
7.80
7.78
6.83
6.81
OH
HOOC
1.00
1.00
1.99
2.03
f1 (ppm)

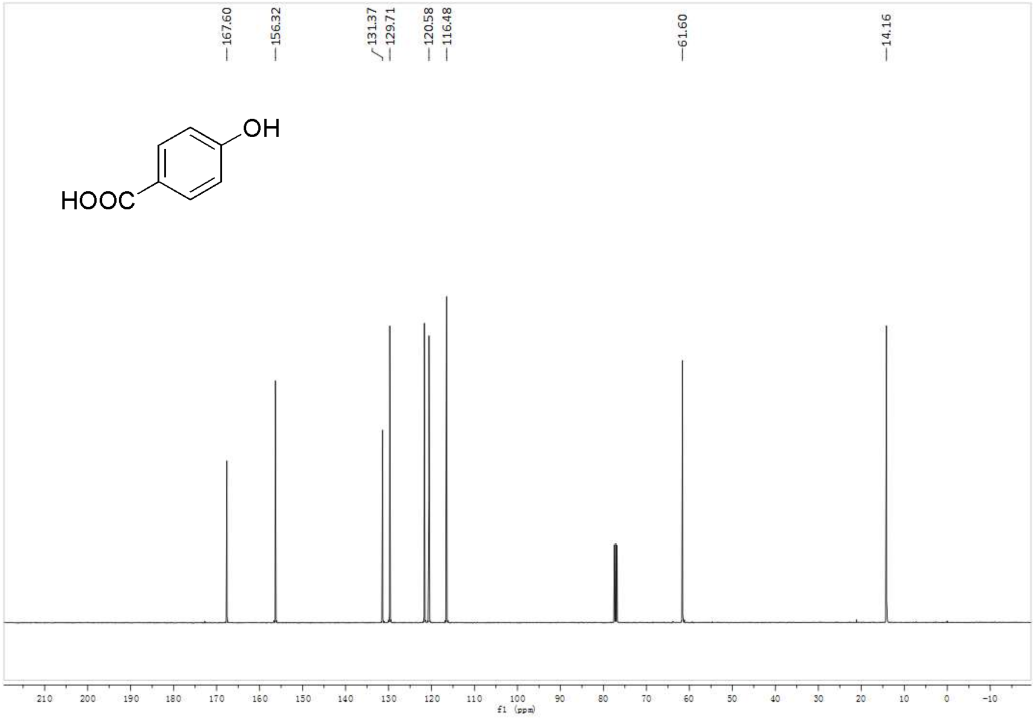

167.60
156.32
131.37
129.71
120.58
116.48
61.60
14.16
OH
HOOC
f1 (ppm)

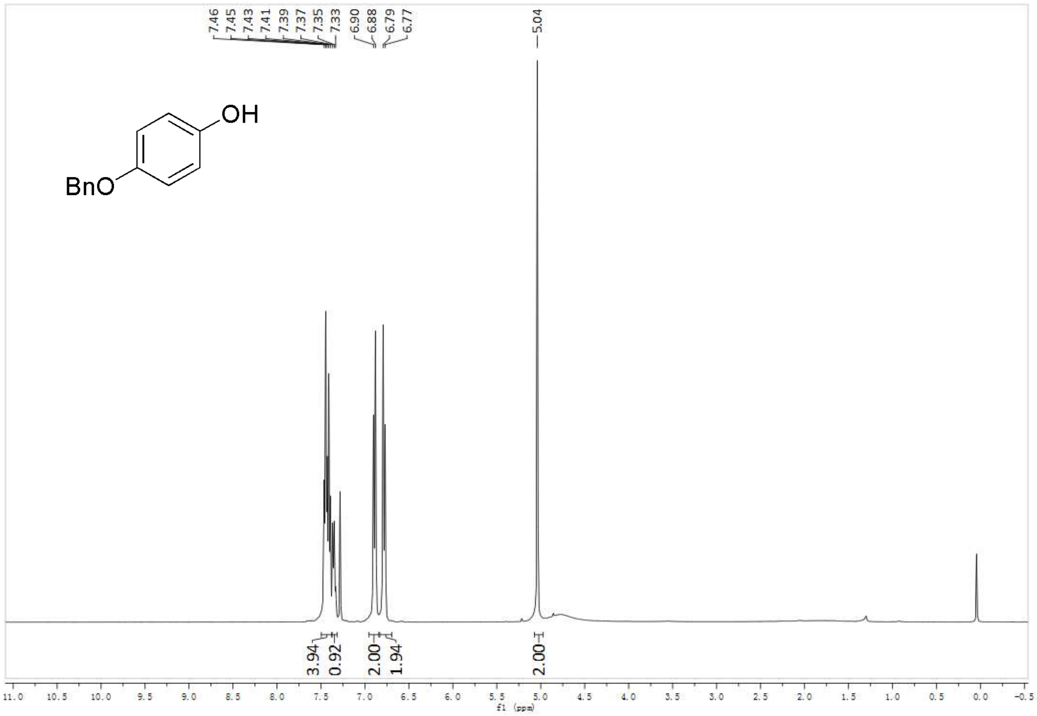
7.46
7.45
7.43
7.41
7.39
7.37
7.35
7.33
6.90
6.88
6.79
6.77
5.04
OH
BnO
3.94
0.92
2.00
1.94
2.00
f1 (ppm)

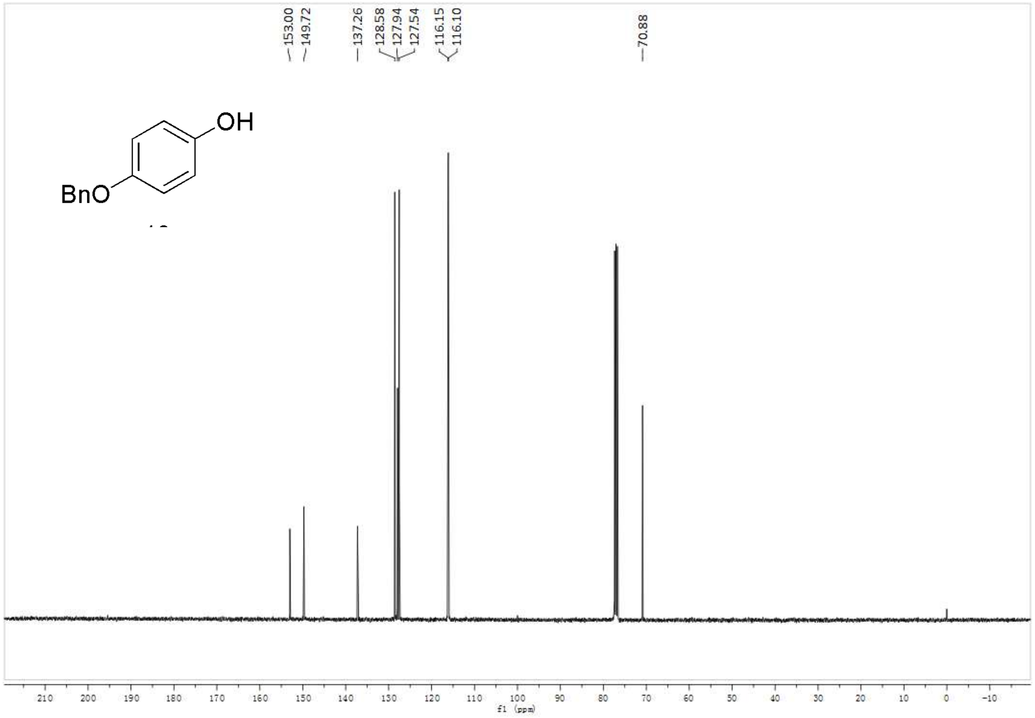
153.00
149.72
137.26
128.58
127.94
127.54
116.15
116.10
70.88
OH
BnO
f1 (ppm)

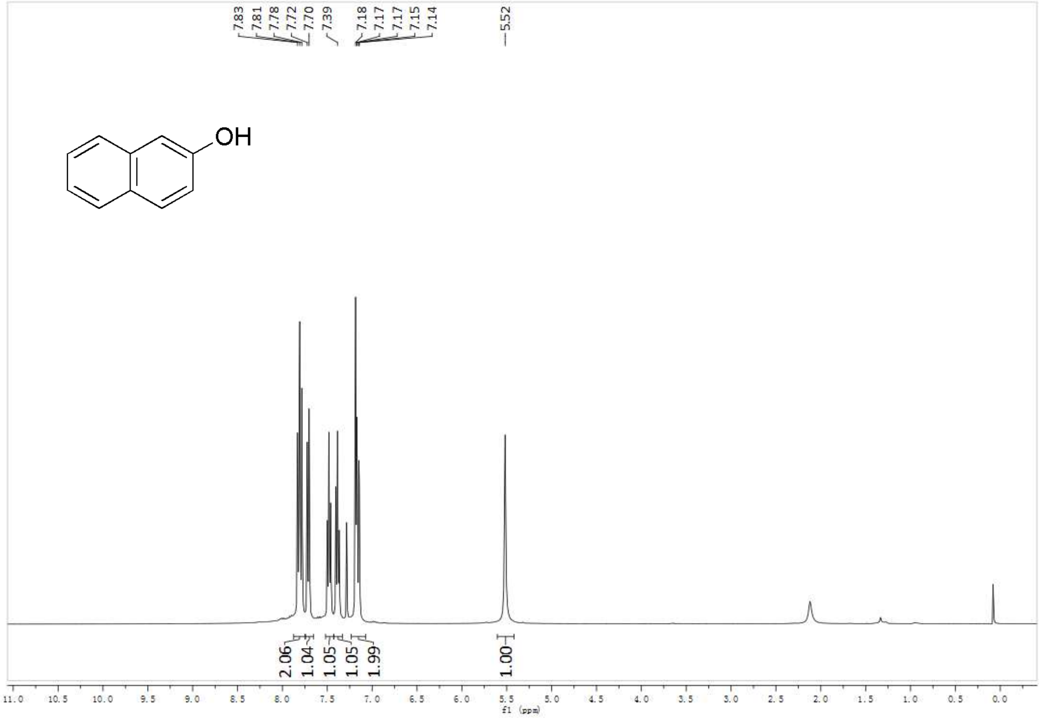
7.83
7.81
7.78
7.72
7.70
7.39
7.18
7.17
7.17
7.15
7.14
5.52
OH
2.06
1.04
1.05
1.05
1.99
1.00
f1 (ppm)

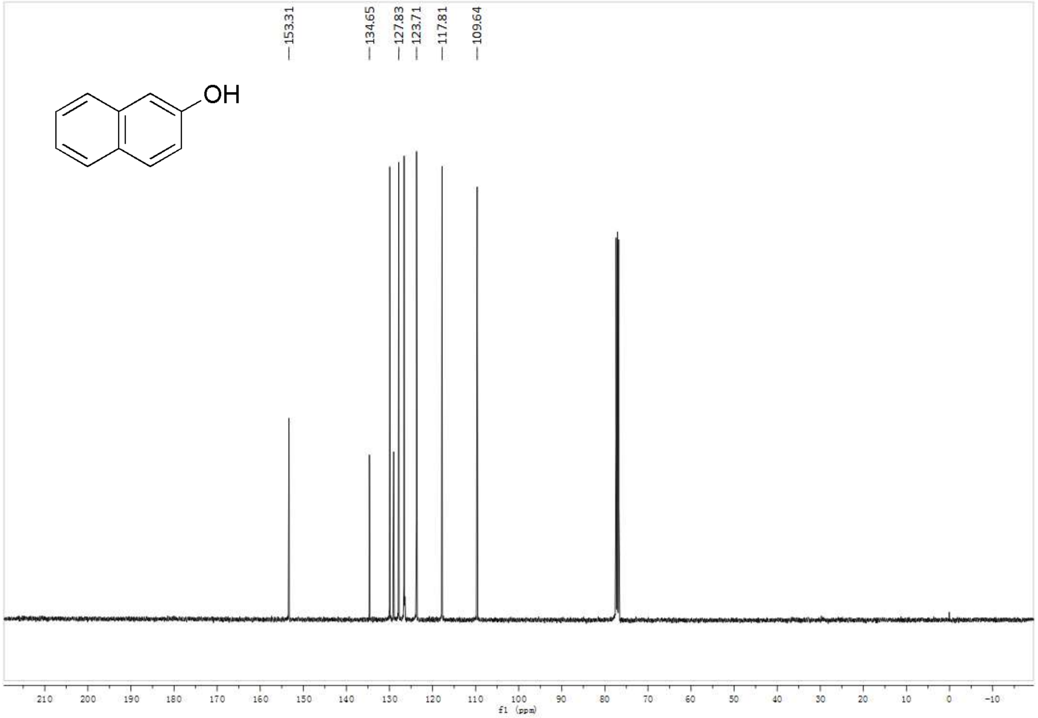
153.31
134.65
127.83
123.71
117.81
109.64
OH
f1 (ppm)

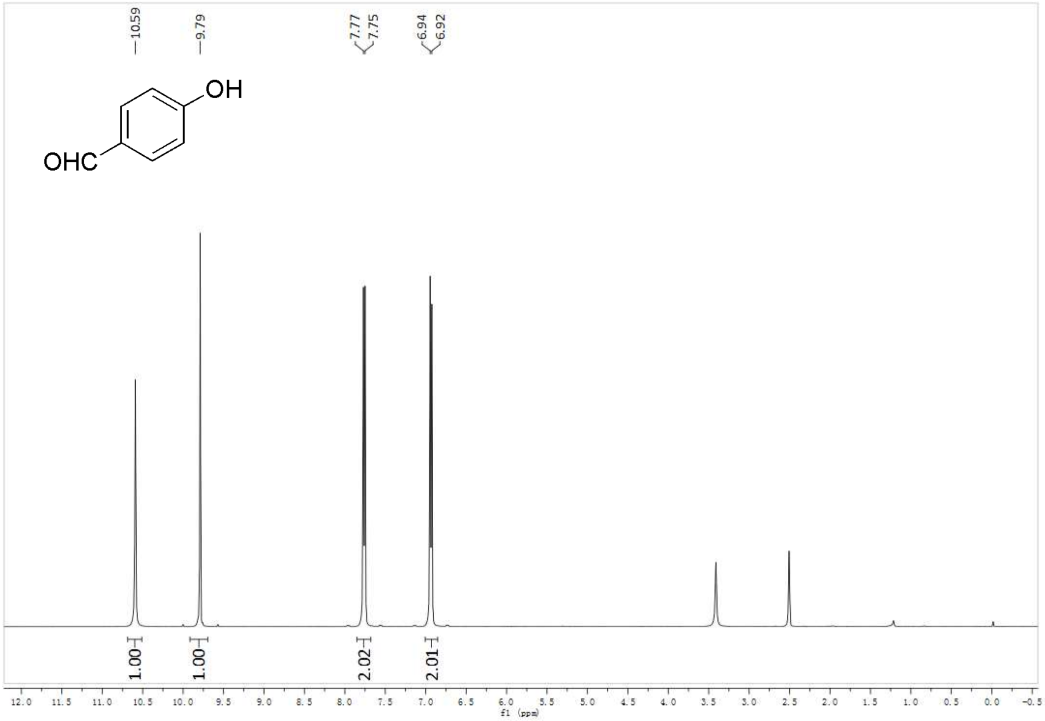
10.59
9.79
7.77
7.75
6.94
6.92
OH
OHC
1.00
1.00
2.02
2.01
f1 (ppm)

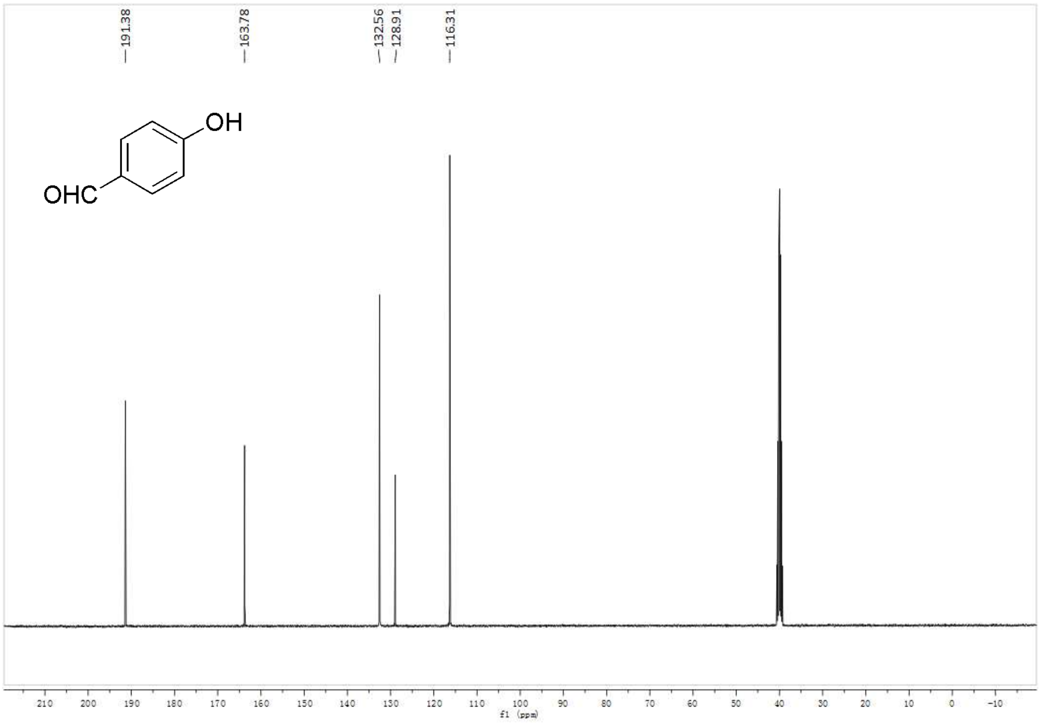
191.38
163.78
132.56
128.91
116.31
OH
OHC
f1 (ppm)

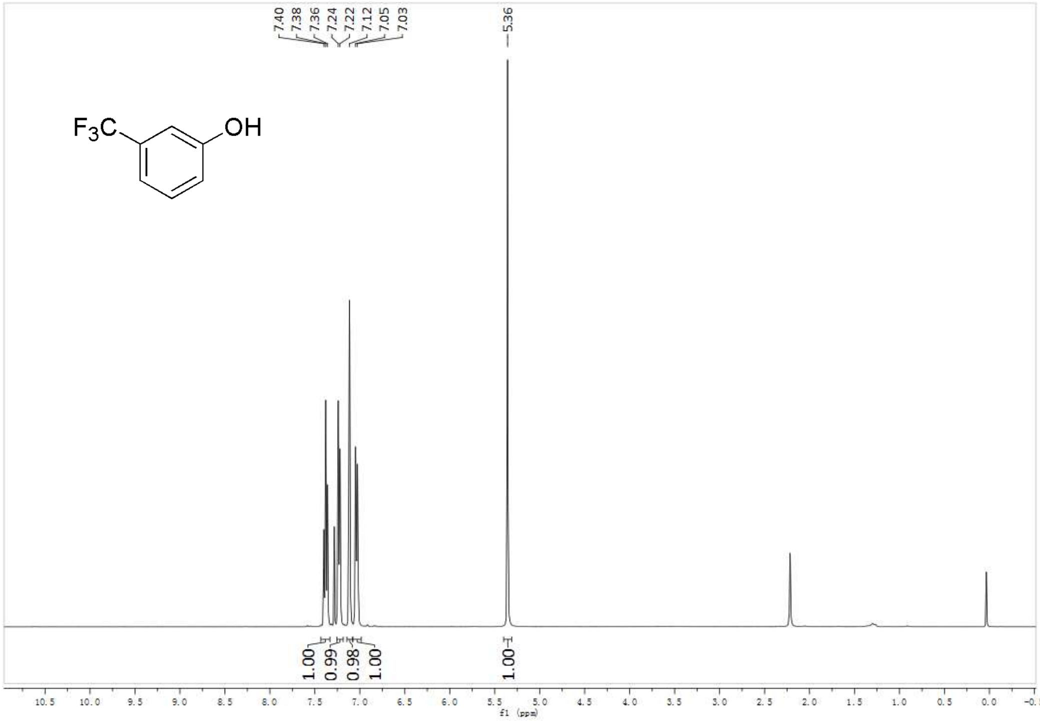
7.40
7.38
7.36
7.24
7.22
7.12
7.05
7.03
5.36
F3C
OH
1.00
0.99
0.98
1.00
1.00
10.5 10.0 9.5 9.0 8.5 8.0 7.5 7.0 6.5 6.0 5.5 5.0 4.5 4.0 3.5 3.0 2.5 2.0 1.5 1.0 0.5 0.0 -0.5
f1 (ppm)

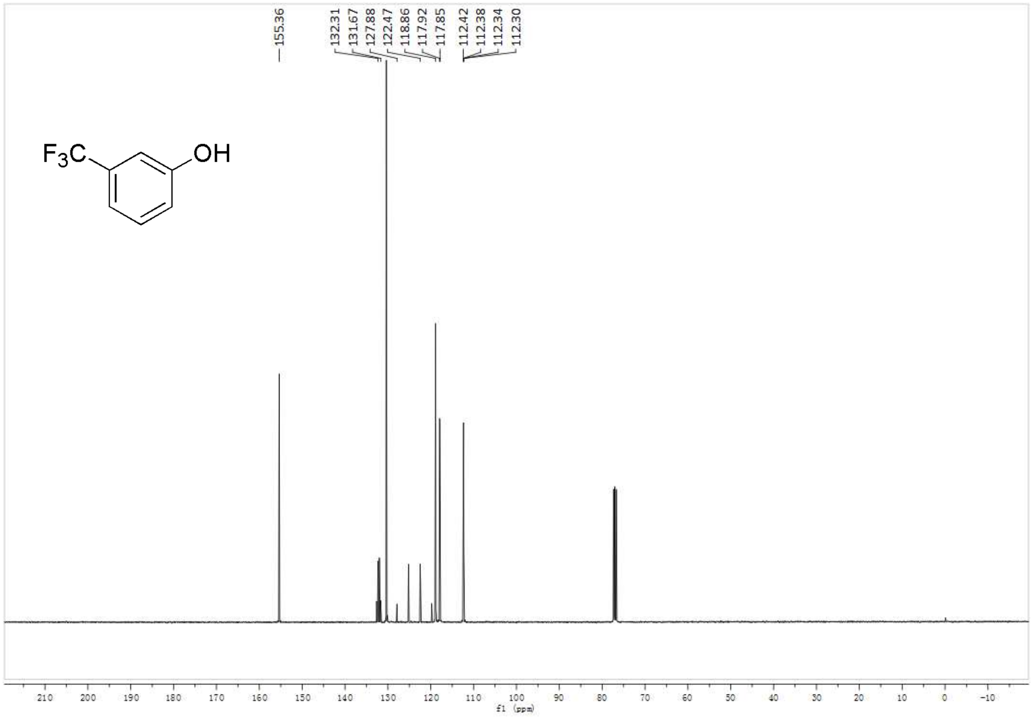
155.36
132.31
131.67
127.88
122.47
118.86
117.92
117.85
112.42
112.38
112.34
112.30
F3C
OH
210 200 190 180 170 160 150 140 130 120 110 100 90 80 70 60 50 40 30 20 10 0 -10
f1 (ppm)

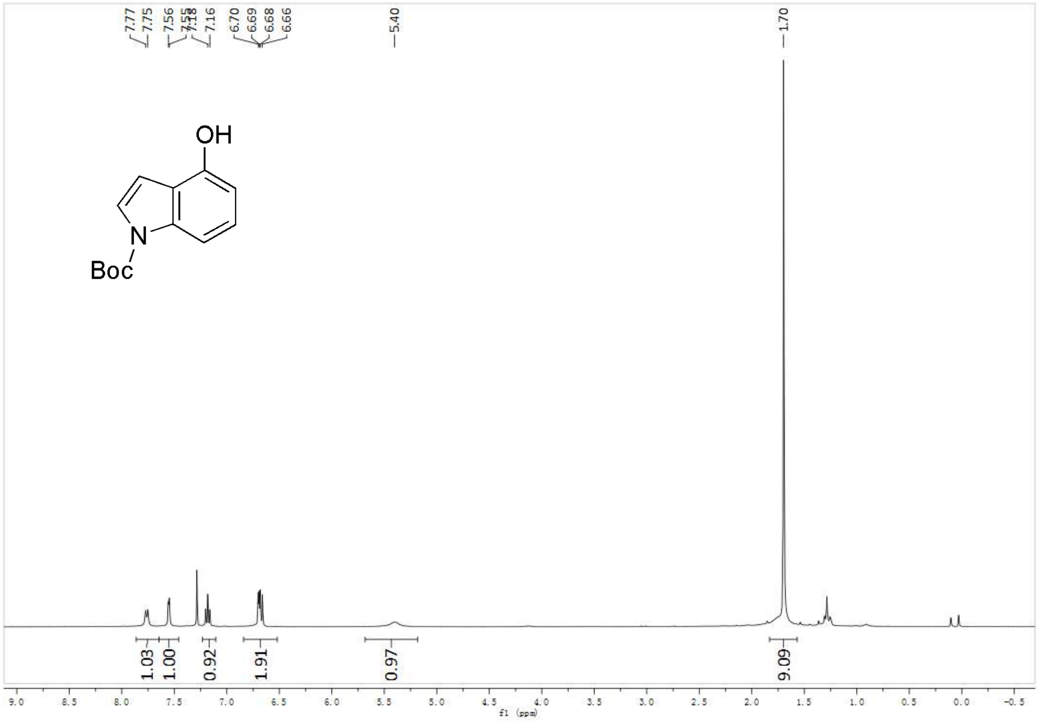
7.77
7.75
7.56
7.55
7.18
7.16
6.70
6.69
6.68
6.66
5.40
1.70
OH
N
Boc
1.03
1.00
0.92
1.91
0.97
9.09
f1 (ppm)

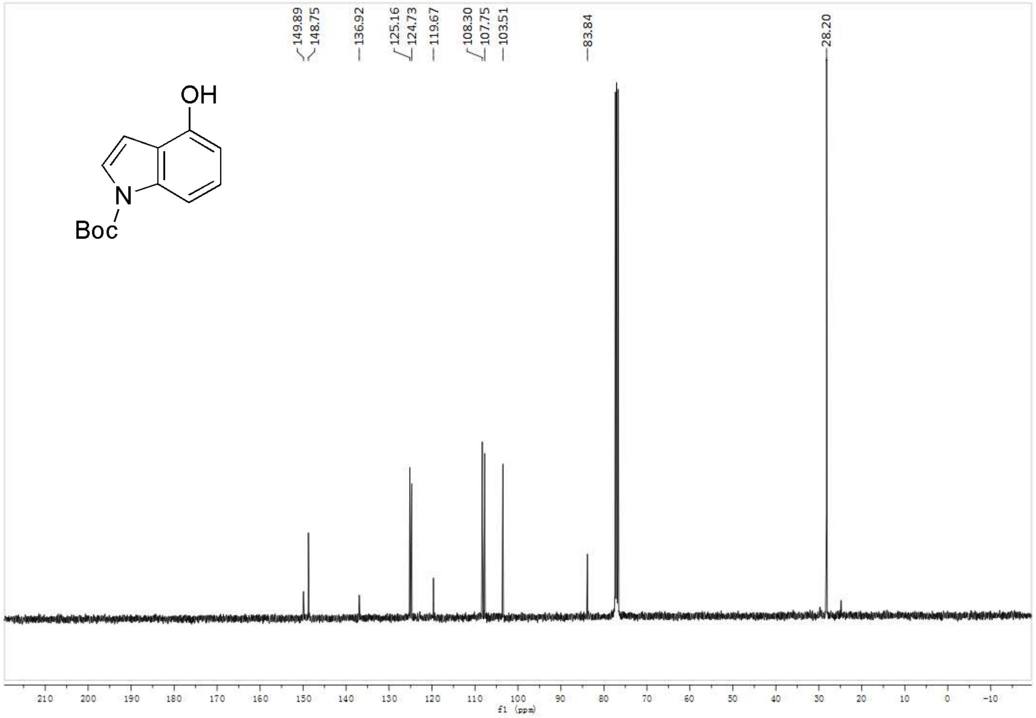
149.89
148.75
136.92
125.16
124.73
119.67
108.30
107.75
103.51
83.84
28.20
OH
N
Boc
f1 (ppm)

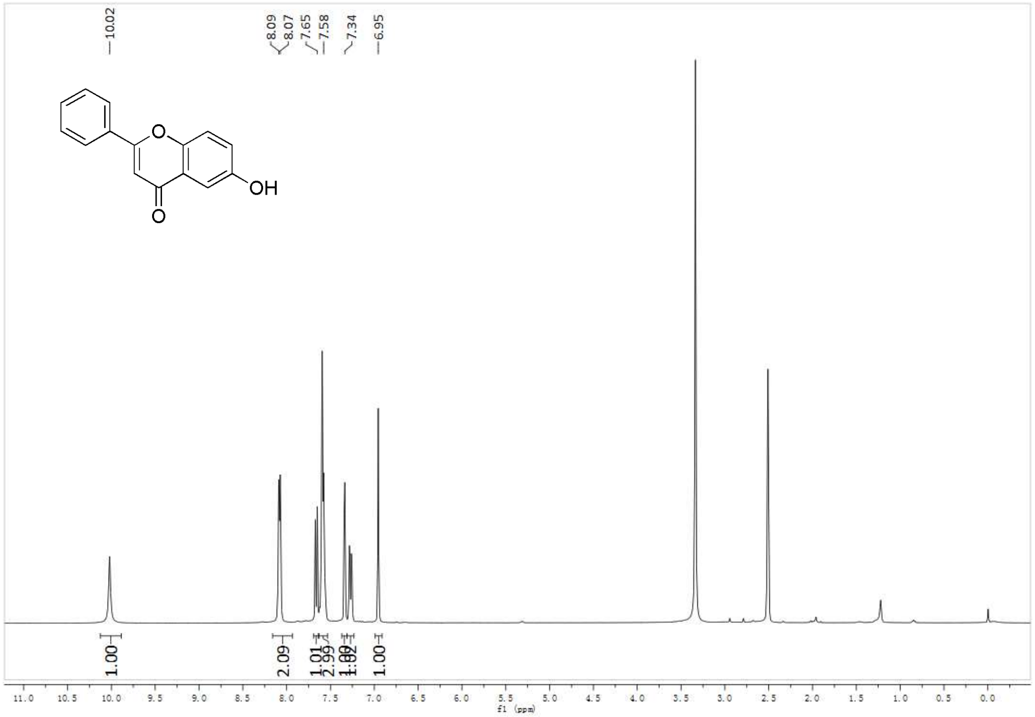

10.02
8.09
8.07
7.65
7.58
7.34
6.95
OH
O
1.00
2.09
1.01
2.99
1.00
1.02
1.00
f1 (ppm)

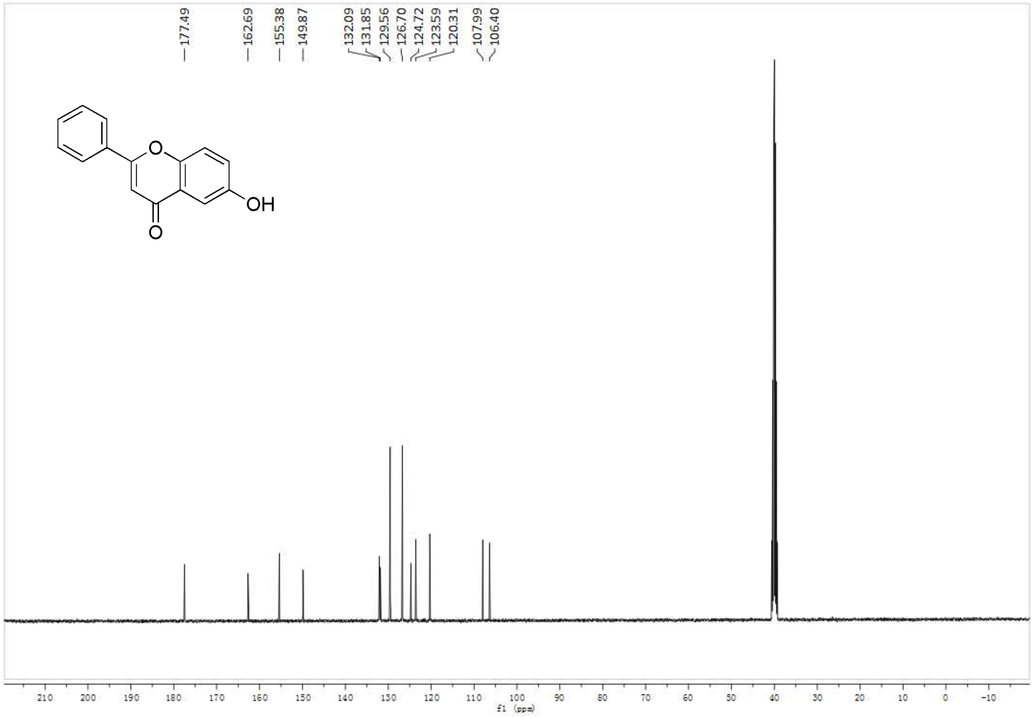

177.49
162.69
155.38
149.87
132.09
131.85
129.56
126.70
124.72
123.59
120.31
107.99
106.40
OH
O
f1 (ppm)

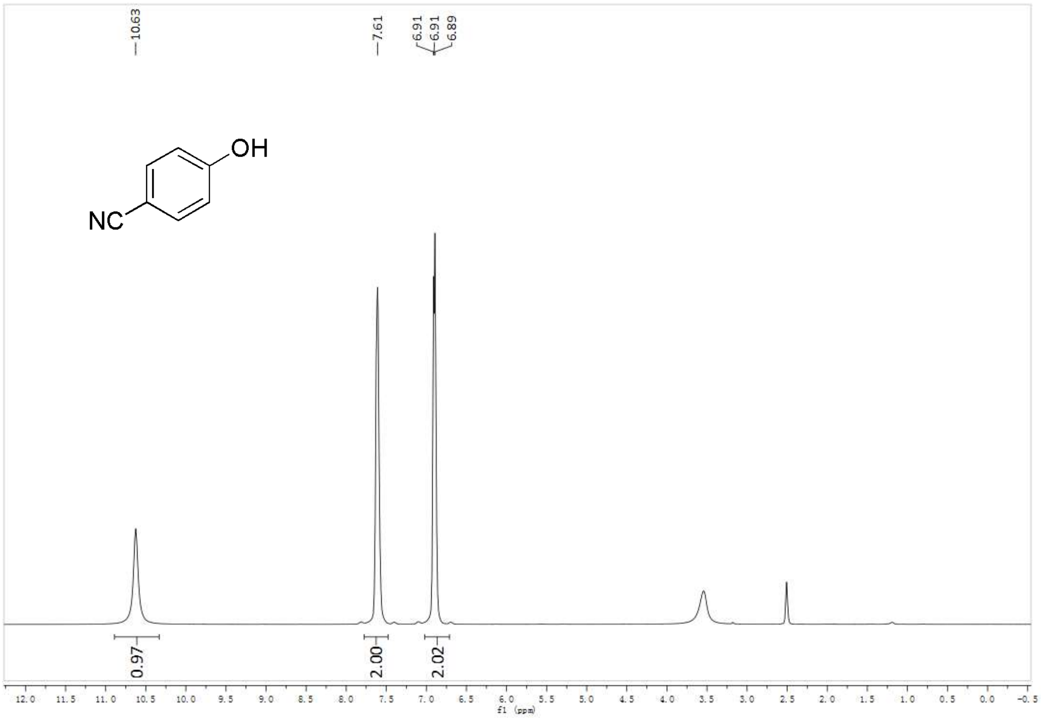

10.63
7.61
6.91
6.91
6.89
OH
NC
0.97
2.00
2.02
12.0 11.5 11.0 10.5 10.0 9.5 9.0 8.5 8.0 7.5 7.0 6.5 6.0 5.5 5.0 4.5 4.0 3.5 3.0 2.5 2.0 1.5 1.0 0.5 0.0 -0.5
f1 (ppm)

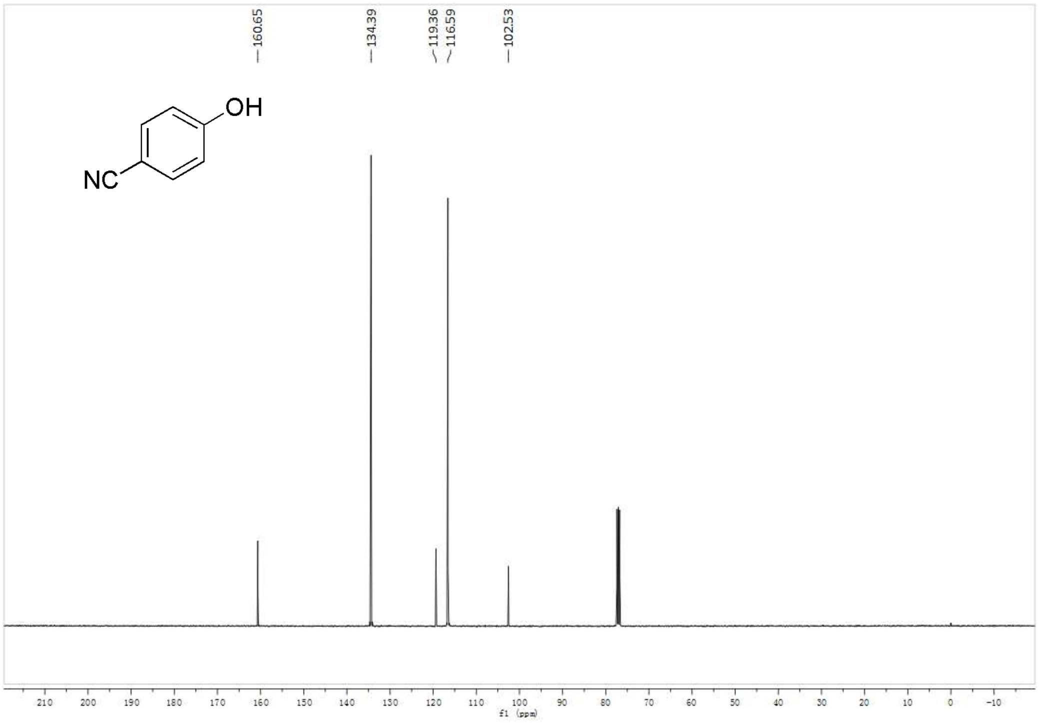

160.65
134.39
119.36
116.59
102.53
OH
NC
210 200 190 180 170 160 150 140 130 120 110 100 90 80 70 60 50 40 30 20 10 0 -10
f1 (ppm)

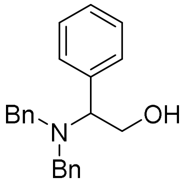

Bn
N
Bn
OH

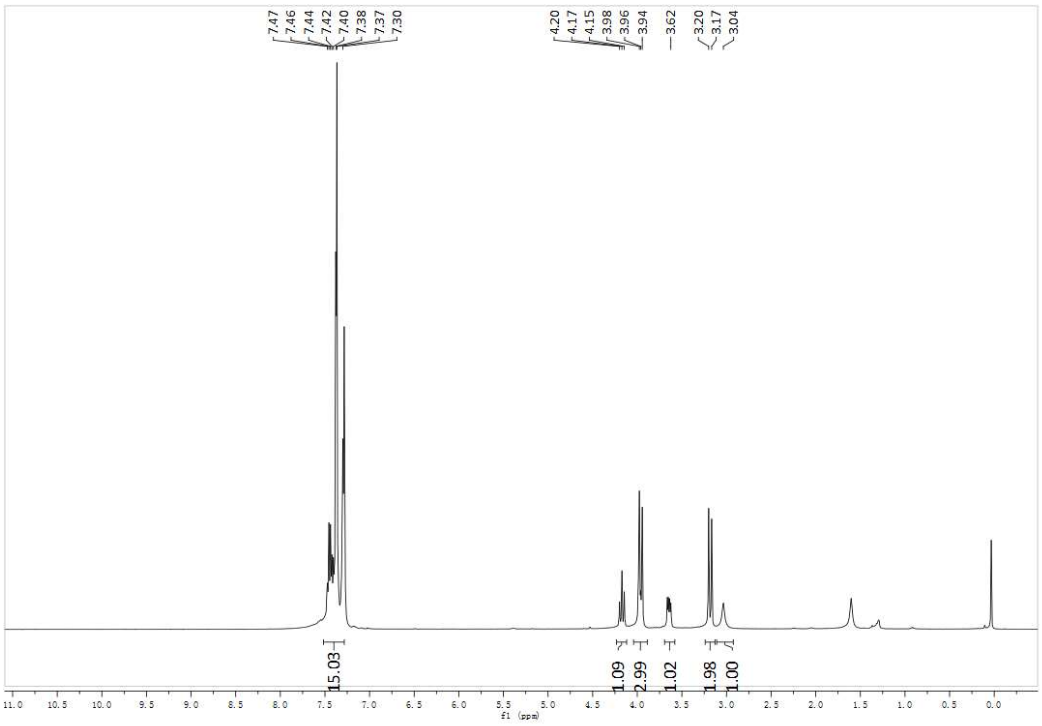

7.47
7.46
7.44
7.42
7.40
7.38
7.37
7.30
4.20
4.17
4.15
3.98
3.96
3.94
3.62
3.20
3.17
3.04
15.03
1.09
2.99
1.02
1.98
1.00
f1 (ppm)

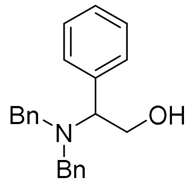

Bn
N
Bn
OH

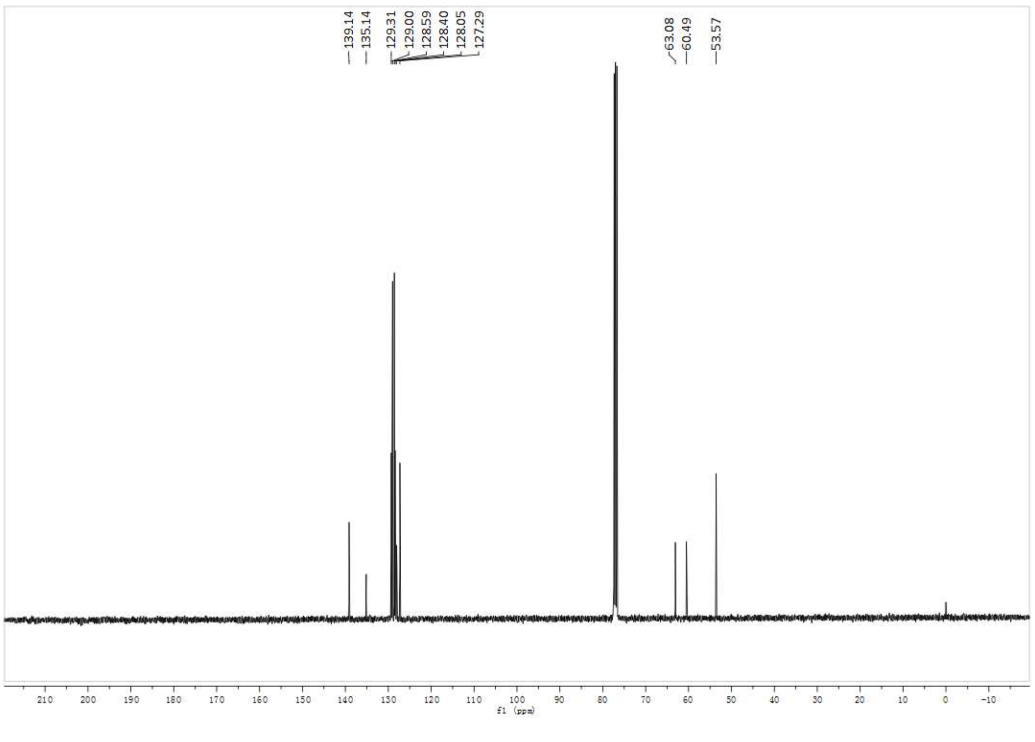

139.14
135.14
129.31
129.00
128.59
128.40
128.05
127.29
63.08
60.49
53.57
f1 (ppm)

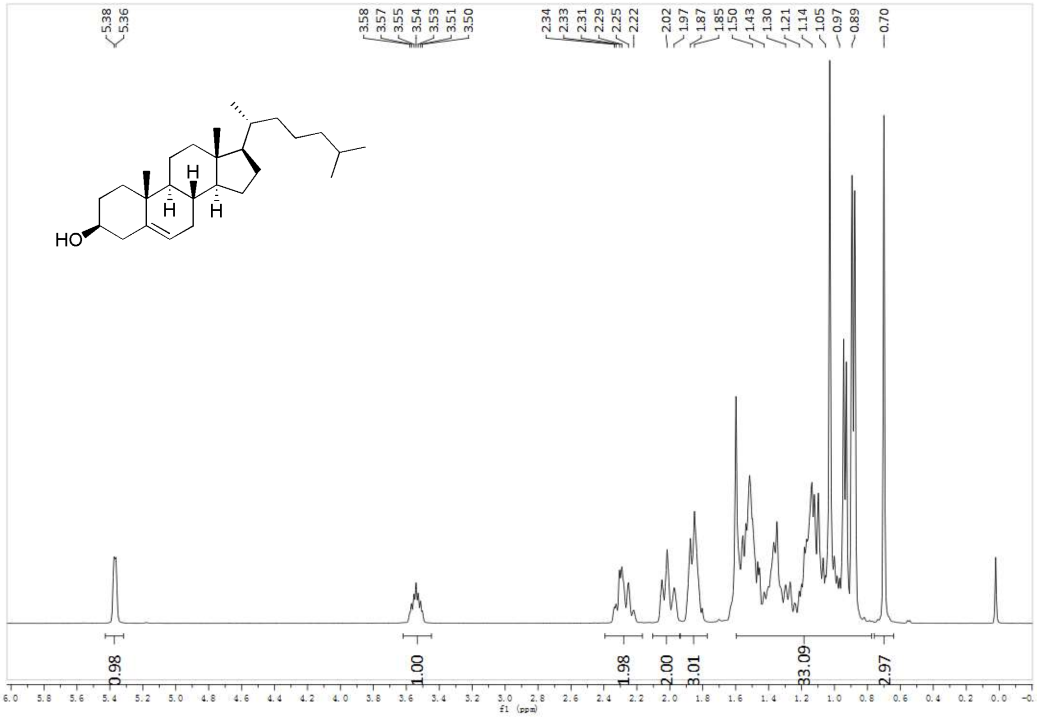

5.38
5.36
3.58
3.57
3.55
3.54
3.53
3.51
3.50
2.34
2.33
2.31
2.29
2.25
2.22
2.02
1.97
1.87
1.85
1.50
1.43
1.30
1.21
1.14
1.05
0.97
0.89
0.70
HO
H
H
H
0.98
1.00
1.98
2.00
3.01
33.09
2.97
f1 (ppm)

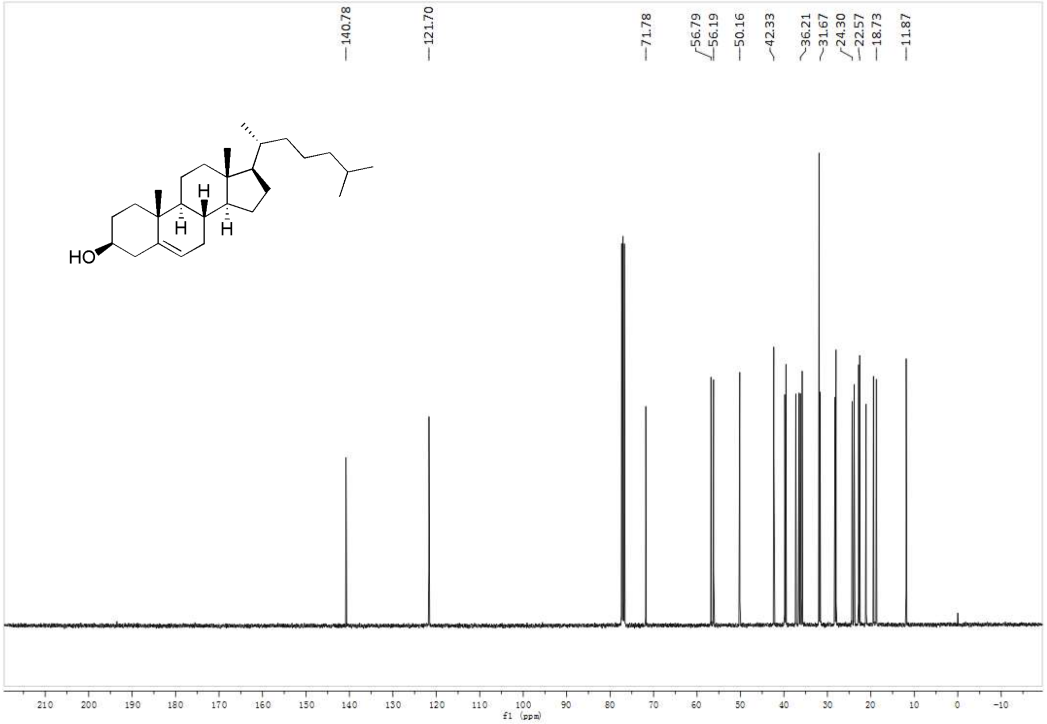

140.78
121.70
71.78
56.79
56.19
50.16
42.33
36.21
31.67
24.30
22.57
18.73
11.87
HO
H
H
H
f1 (ppm)

5. Reference

1. Lipshutz, B. H.; Ghorai, S.; Leong, W. W. Y. *J. Org. Chem.* **2009**, *74*, 2854-2857.
2. Matt, C.; Kölblin, F.; Streuff, J. *Org. Lett.* **2019**, *21*, 6983-6988.
3. Li, S.; Li, F.; Gong, J.; Yang, Z. *Org. Lett.* **2015**, *17*, 1240-1243.
4. Keukeleire, D. D.; He, S.-L. *J. Photochem. Photobid. A: Chem.* **1994**, *80*, 233-240.
5. Tang, L.; Yang, Z.; Jiao, J.; Yang, Y.; Zhao, Y.; Cui, J. *Faming Zhuanli Shenqing* **2019**, CN 109320489 A 20190212.
6. Jean. M.; van de Weghe, P. *Tetrahedron Letters* **2011**, *52*, 3509–3513.
7. Shi, L.; Narula, C. K.; Mak, K. T.; Kao, L.; Xu, Y.; Heck, R. F. *J. Org. Chem.* **1983**, *48*, 3894-3900.
8. Rochette, E. M.; Lewis, W.; Dossetter, A. G.; Stockman, R. A. *Chem. Commun.* **2013**, *49*, 9395—9397.
9. Hiroshi, H.; Isa, N. *Jpn. Kokai Tokkyo Koho* **2015**, 2015203019.
10. Davis, T. A.; Hyster, T. K.; Rovis, T. *Angew. Chem. Int. Ed.* **2013**, *52*, 14181–14185.
11. Lu, Z.; Zeng, X.; Hammond, G. B.; Xu, B. *J. Am. Chem. Soc.* **2017**, *139*, 18202-18205.
12. McKinley, N. F. O'Shea, D. F. *J. Org. Chem.* **2004**, *69*, 5087-5092.
13. Andrei V., A.; Alexander V., V.; Hideaki, F. *Bull. Chem. Soc. Jpn.* **1996**, *69*, 933-45.
14. Koura, M.; Matsuda, T.; Okuda, A.; Watanabe, Y.; Yamaguchi,Y.; Kurobuchi, S.; Matsumoto, Y.; Shibuya, K. *Bioorg. Med. Chem. Lett.* **2015**, *25*, 2668–2674.
15. Pilli, R.; Balakrishnan, V.; Chandrasekaran, R.; Rasappan, R. *Org. Biomol. Chem.* **2019**, *17*, 1749–1753.
16. Rohrbach, S.; Shah, R. S.; Tuttle, T.; Murphy, J. A. *Angew. Chem. Int. Ed.* **2019**, *58*, 11454 –11458.
17. Mao, Y.; Liu, Y.; Hu, Y.; Wang, L.; Zhang, S.; Wang, W. *ACS Catal.* **2018**, *8*, 3016−3020.
18. Liu, H.; Ge, L.; Wang, D.-X.; Chen, N.; Feng, C. *Angew. Chem. Int. Ed.* **2019**, *58*, 3918 –3922.
19. Pisanenko, D. A.; Nesterenko, S. A. *Zhurnal Organicheskoi Khimii,* **1979**, *15*, 1911-1915.
20. Oh, H.; Park, A.; Jeong K.-S.; Han, S. B.; Lee, H. *Adv. Syn. Catal.* **2019**, *361*, 2136-2140.
21. Zou, Y.-Q.; Chen, J.R.; Liu, X.-P.; Lu, L.Q.; Davis, R. L.; Jørgensen, K. A.; Xiao, W.-J. *Angew. Chem. Int. Ed.* **2012**, *51*, 784 –788.
22. Fier, P. S.; Maloney, K. M. *Org. Lett.* **2017**, *19*, 3033−3036.
23. Hua, Y.; Asgari, P.; Avullala, T.; Jeon, J. *J. Am. Chem. Soc.* **2016**, *138*, 7982−7991.
24. Xiao,Y.; Xu, Y.; Cheon, H. S.; Chae, J. *J. Org. Chem.* **2013**, *78*, 5804−5809.
25. Wei, P.-F.; Qi, M.-Z.; Wang, Z. P.; Ding, S.Y.; Yu, W.; Liu, Q.; Wang, L.K.; Wang, H.-Z.; An, W.-K.; Wang, W. *J. Am. Chem. Soc.* **2018**, *140*, 4623−4631.
26. Luo, J.; Hu, B.; Sam, A.; Liu, T. L. *Org. Lett.* **2018**, *20*, 361-364.
27. Sun, N.; Wang, C.; Wang, H.; Yang, L.; Jin, P.; Zhang, W.; Jiang, J. *Angew. Chem. Int. Ed.* **2019**, *58*, 18011 – 18016.
28. Xu, Y.-T.; Li, C.-Y.; Huang, X.-B.; Gao, W.-X.; Zhou,Y.-B.; Liu, M.-C.; Wu, H.-Y. *Green Chem.* **2019**, *21*, 4971–4975.
29. Nobuyoshi A. et al, *PCT Int. Appl.*, **2008**, WO2008047831.
30. Yang, L.; Huang, Z.; Li, G.; Zhang, W.; Cao, R.; Wang, C.; Xiao, J.; Xue, D. *Angew. Chem. Int. Ed.* **2018**, *57*, 1968 –1972.
31. Chong, H.-S.; Chen. Y. *Org. Lett.* **2013**, *15*, 5912-5915.

32. Kern, N.; Dombray, T.; Blanc,´A..; Weibel, J.-M.; Pale. P. *J. Org. Chem.* **2012**, *77*, 9227−9235.
